# JN.1-adapted vaccination is associated with readjustment of ancestral memory B cells toward neutralization within the JN.1 antigenic space

**DOI:** 10.1038/s41467-026-76035-z

**Published:** 2026-07-24

**Authors:** Metodi V. Stankov, Matthias Bruhn, Markus Hoffmann, Abdus Salam, Amy Eichmann, Inga Nehlmeier, Luis A. Manthey, Torsten Witte, Stefan Pöhlmann, Gerrit Ahrenstorf, Christine Happle, Alexandra Dopfer-Jablonka, Ulrich Kalinke, Georg M. N. Behrens

**Affiliations:** 1 Department of Rheumatology and Immunology, Hannover Medical School, Hannover, Germany; 2 Institute for Experimental Infection Research, TWINCORE, Centre for Experimental and Clinical Infection Research, a joint venture between the Helmholtz Centre for Infection Research and the Hannover Medical School, Hannover, Germany; 3 Infection Biology Unit, German Primate Center – Leibniz Institute for Primate Research, Göttingen, Germany; 4 Faculty of Biology and Psychology, Georg-August-University Göttingen, Göttingen, Germany; 5 Department of Pediatric Pulmonology, Allergology and Neonatology, Hannover Medical School, Hannover, Germany; 6 Biomedical Research in Endstage and Obstructive Lung Disease Hannover (BREATH), German Center for Lung Research (DZL), Hannover, Germany; 7 Cluster of Excellence RESIST (EXC 2155), Hannover Medical School, Hannover, Germany; 8 German Center for Infection Research (DZIF), partner site Hannover-Braunschweig, Hannover, Germany; 9 Center for Individualized Infection Medicine (CiiM), Hannover, Germany

**Keywords:** Antibodies, RNA vaccines

## Abstract

The antigenic drift of SARS-CoV-2 toward the JN.1 lineage has prompted the development of variant-adapted COVID-19 booster vaccines. However, these boosters are thought to primarily recall pre-existing memory B cells (MBC), raising concerns about their ability to realign the immune response in highly pre-exposed populations. Here we analyze antibody and B cell responses in pre-exposed individuals (*n* = 42; median 4.5 prior COVID-19 vaccinations; 90% with at least one prior SARS-CoV-2 infection) following vaccination with a JN.1-adapted mRNA vaccine. Vaccination is associated with increased IgG binding and enhanced neutralization of JN.1 and related descendant variants. Longitudinal profiling of antigen-specific MBC shows that Wu01-only and Wu01/JN.1 cross-reactive cells remain dominant, while JN.1-only cells modestly increase by day 21. Single-cell RNA-sequencing of antigen-specific MBC in a representative sub-cohort (*n* = *7*), combined with functional monoclonal antibody analyses, demonstrates that somatic hypermutation (SHM) drives intra-clonotype specialization toward improved JN.1 binding and neutralization. These findings indicate maturation of pre-existing, class-switched MBC rather than substantial de novo recruitment of naïve B cells. In conclusion, JN.1-adapted booster vaccination is associated with refinement of pre-existing MBC repertoires toward the JN.1 antigenic space and with enhanced neutralization of contemporary and antigenically proximate variants.

## Abstract (web summary)

Because of the continuous evolution of SARS-CoV-2, pre-existing immune responses may not be as effective against new viral variants. Here the authors investigate whether B cell responses to ancestral SARS-CoV-2 are stimulated by JN.1-adapted booster vaccines and propose that there is somatic hypermutation of existing B cell clones rather than de novo generation from naive B cells.

## Introduction

**Fig. 1:**
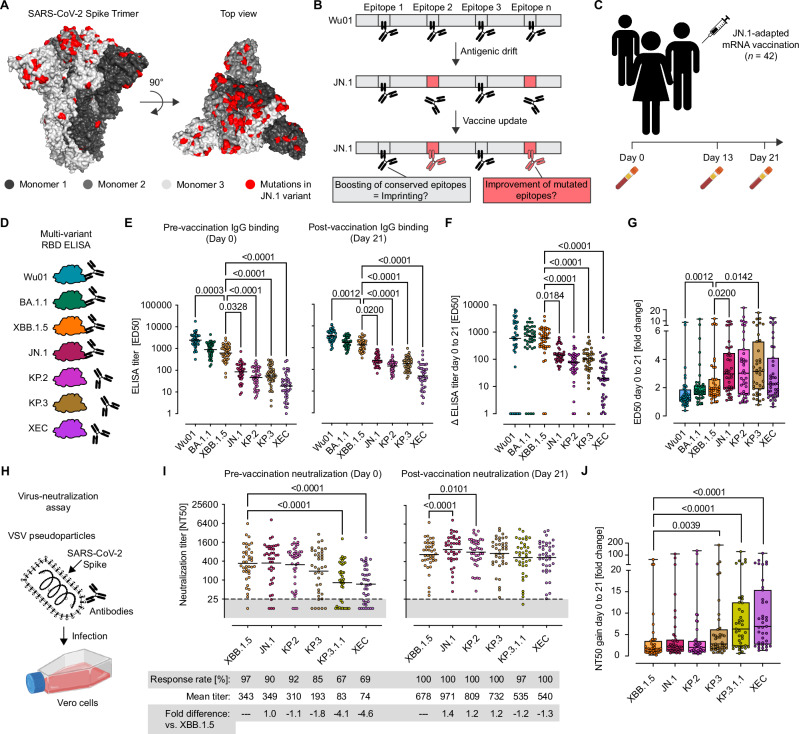
Improved serum binding and neutralization after JN.1-adapted mRNA booster vaccination. **A** Three-dimensional location of amino-acid substitutions present in the SARS-CoV-2 spike of the JN.1 variant. **B** Schematic depiction of antibody binding toward conserved versus altered epitopes under antigenic drift and variant-adapted booster vaccination. **C** Study design and sampling time points. **D** SARS-CoV-2 variants used for the multi-variant ELISA. **E** Serum IgG binding to variant RBDs quantified as ED50 (50% effective dilution) derived from 4-parameter logistic fits of titration curves (left: day 0, *n* = 42; right: day 21, *n* = 39). **F** Absolute increase and **G** fold change in ED50 between day 0 and day 21 for each variant (*n* = 39, paired). Negative values were plotted at y = 1 in the logarithmic visualization. **H** Conceptual scheme of the pseudovirus neutralization assay. **I** Serum neutralization titers (NT50) of the indicated variants (left: day 0; right: day 21; *n* = 39, paired). **J** Fold change in NT50 from day 0 to day 21 (*n* = 39, paired). Groups were compared using Friedman tests (two-sided) followed by Dunn’s multiple-comparisons tests (corrected for multiple comparisons) comparing each variant to XBB.1.5 (reference control variant); (adjusted *p*-values shown in the plots/Source Data). Each dot represents one participant; horizontal lines indicate medians (and IQR, if shown). ELISA data are averaged from 4 independent experiments; neutralization data are averaged from 2 independent experiments with 4 technical replicates per dilution step; n refers to biological replicates (individual participants), across which statistical comparisons were performed. In panels (**E**) and (**I**), lines indicate geometric means; in panels (**F**), horizontal lines indicate medians only (no whiskers shown); in panels (**G**) and (**J**), box plots show the median (center line), 25–75th percentiles (box bounds), and minimum-to-maximum values (whiskers), with individual data points overlaid. Icons in (**B**–**D**, **H**) were created in BioRender. Kalinke, U. (2026) https://BioRender.com/h12c5rj.

SARS-CoV-2 continuously evolves variants that evade the immunity elicited by prior infections and/or vaccinations^1,2^. In the context of other endemic infectious diseases such as seasonal influenza, gradual genetic changes, historically referred to as antigenic drift, allow for constant circulation in immunologically non-naïve populations^3^. Antigenic shift, defined by abrupt, large-scale changes, can give rise to antigenically distinct influenza viruses with pandemic potential^3^. For COVID-19, evidence supports the occurrence of both antigenic drift and shift^4,5^. Pathogens with high genetic plasticity pose a major challenge for vaccine development, as the trajectory of future mutations remains unpredictable. To increase circulating antibody levels and improve protection against emerging virus variants, variant-adapted booster vaccines have been developed, in which the antigenic component is updated to match currently circulating virus variants^6–10^. By late 2023, the JN.1 variant had become the dominant SARS-CoV-2 strain in various countries^11^. Consequently, the JN.1-adapted mRNA vaccine “Comirnaty JN.1” was authorized by the European Medicines Agency in July 2024. This vaccine encodes a spike (S) protein containing numerous mutations when compared with the ancestral SARS-CoV-2 strain (Fig. 1A).

When the human immune system is primed against an ancestral antigen and subsequently challenged with a modified version of the same antigen, immune imprinting may become relevant. This phenomenon describes how recall responses of B cells are predominantly driven by pre-existing MBC that were elicited earlier, e.g., in the context of an XBB.1.5-adapted COVID-19 booster vaccine^12,13^. It remains under debate to what extent re-exposure to a modified antigen induces antibodies that are directed against previously unrecognized epitopes^14,15^ (Fig. 1B). So-called type-specific B cells, which preferentially recognize the drifted epitopes of the Delta and Omicron BA.1 variants, are largely undetectable, likely reflecting the low immunogenicity of these sites^16^. In highly pre-exposed populations, this imprinting-dominated baseline complicates interpretation of variant-adapted booster responses, because increases in binding magnitude may remain modest even when neutralization improves through qualitative changes in the antibody repertoire.

One concept proposed to explain the broadening of antibody responses upon repeated vaccination is antibody feedback, in which pre-existing serum antibodies mask certain epitopes and thereby suppress the corresponding responses, ultimately biasing germinal center reactions towards epitopes that are not yet masked^17^. In parallel, MBC acquire somatic hypermutations (SHM) in the aftermath of an immunization, and some SHM were found to stochastically match virus variants that had not yet emerged^18,19^. This phenomenon likely evolved as a mechanism to suppress viral escape by reinforcing the Achilles’ heel of individual antibodies^20^. MBC that underwent such anticipatory maturation are subsequently available to undergo recall responses upon re-exposure with the same or a related antigen^21^. Together, antibody feedback and SHM-driven diversification provide a mechanistic framework by which variant-adapted booster vaccination could impose directional selection within pre-existing memory clonotypes, thereby improving recognition of antigenically evolved virus variants.

Here, we show that vaccination with a JN.1-adapted mRNA vaccine enhances antibody binding to the JN.1 S protein and neutralization of the JN.1 variant, and that these responses extend to binding and neutralization of the newly emerged variants KP.3 and XEC despite their substantial immune evasion. Using single-cell RNA-sequencing of MBC together with expression of recombinant monoclonal antibodies, we provide mechanistic insight into the ongoing B cell evolution that counteracts antigenic drift of the SARS-CoV-2 S protein, including intra-clonotype specialization consistent with SHM-driven antibody adaptation to viral immune-escape variants.

## Results

### JN.1-adapted mRNA vaccination improves neutralization of SARS-CoV-2 variants despite persistent binding gaps

In this study, we assessed immune responses (immunoglobulin G, IgG) in 42 healthcare workers participating in the COVID-19 Contact (CoCo) Study^22^ who were vaccinated with Comirnaty JN.1 (Fig. 1C). The cohort was highly pre-exposed, with a median of 4.5 previous COVID-19 vaccinations, and 90% of individuals reporting at least one prior SARS-CoV-2 infection (Supplementary Table S1). The paired analyses include only donors with data both before and after vaccination (see figure legends and Source Data). We characterized the propensity of emerging/circulating SARS-CoV-2 variants to escape from host antibody responses by testing sera with a multi-variant receptor-binding domain (RBD) ELISA that covers Wu01, BA.1.1, XBB.1.5, JN.1, KP.2, KP.3, and XEC (Fig. 1D).

At day 0 (*n* = 42), ED50 titers showed a pronounced antigenic gradient, with the highest geometric mean values for Wu01 and progressively lower titers toward antigenically evolved variants (geometric means: Wu01 2438; BA.1.1 903; XBB.1.5 621; JN.1 84.1; KP.2 46.7; KP.3 54.4; XEC 18.7). Across variants, differences were significant (*p* < 0.0001; Fig. 1E), and compared with XBB.1.5, binding was significantly lower to JN.1 (*p* = 0.0328), KP.2 (*p* < 0.0001), KP.3 (*p* < 0.0001), and XEC (*p* < 0.0001), consistent with a major step in antigenic distance between XBB.1.5 and the JN.1 lineage. At day 21, ED50 values increased across all variants, but the binding hierarchy persisted (Fig. 1E). The increase in ED50 (day 21 minus day 0) was similar for Wu01, BA.1.1, and XBB.1.5, and significantly lower for JN.1 and the descendant variants (Fig. 1F), pointing towards a certain level of immunological imprinting that favors binding of the ancestral antigen. Because baseline ED50 titers against JN.1-lineage variants were low, their relative increases (fold changes) were proportionally larger despite smaller absolute gains. Accordingly, ED50 fold changes (day 21/day 0) were greatest for JN.1 and its descendants (Fig. 1G). In addition, baseline ED50 values inversely correlated with fold change across variants (Supplementary Fig. S1A), suggesting ceiling-limited boosting in this highly pre-exposed cohort. Also, the baseline neutralization titers inversely correlated with fold changes, including for JN.1 (*ρ* = −0.668*, p* = 1.58 × 10⁻⁵*; n* = 39), KP.3.1.1 (*ρ* = −0.461*, p* = 0.0030*; n* = 39) and XEC (*ρ* = −0.357*, p* = 0.0259*; n* = 39) (Supplementary Fig. S1B). Next, we determined the neutralization titers of serum samples using vesicular stomatitis virus (VSV) pseudoparticles that were engineered to display variants of the SARS-CoV-2 S protein (Fig. 1H). Before vaccination, the sera already neutralized several virus variants such as JN.1, but immune escape was significant for KP.3.1.1 and XEC (Fig. 1I). After vaccination, neutralizing titers were strongest for JN.1, the variant encoded by the vaccine, and the sera also potently neutralized KP.3.1.1 and XEC (Fig. 1I). As observed for the IgG ED50 binding values, fold changes in NT50 were largest for the most recent variants such as KP.3.1.1 and XEC (Fig. 1J). Taking the cohort heterogeneity into account, we assessed whether post-vaccination antibody levels for selected variants (XBB.1.5, JN.1, and XEC) were associated with baseline antibody titers, number of prior vaccinations, time since last vaccination, and number of SARS-CoV-2 infections. We confirmed a strong association between baseline binding or neutralizing titers and post-vaccination titers. Beside this, only the time since last vaccination was significantly associated with post-vaccination ED50 and NT50 against XBB.1.5, and with NT50 against JN.1, suggesting that baseline antibody levels were the main determinant of post-vaccination responses (Supplementary Data 3). Taken together, serum IgG binding (ED50) and neutralization (NT50) data indicate a qualitative shift in antibody function toward JN.1-lineage variants, despite persistent and quantitatively dominating binding patterns to ancestral virus strains. We hypothesized that these differences result from MBC maturation and therefore investigated the MBC response to vaccination with the variant-adapted mRNA booster.

### Selective enhancement of JN.1-reactive MBC after vaccination

**Fig. 2:**
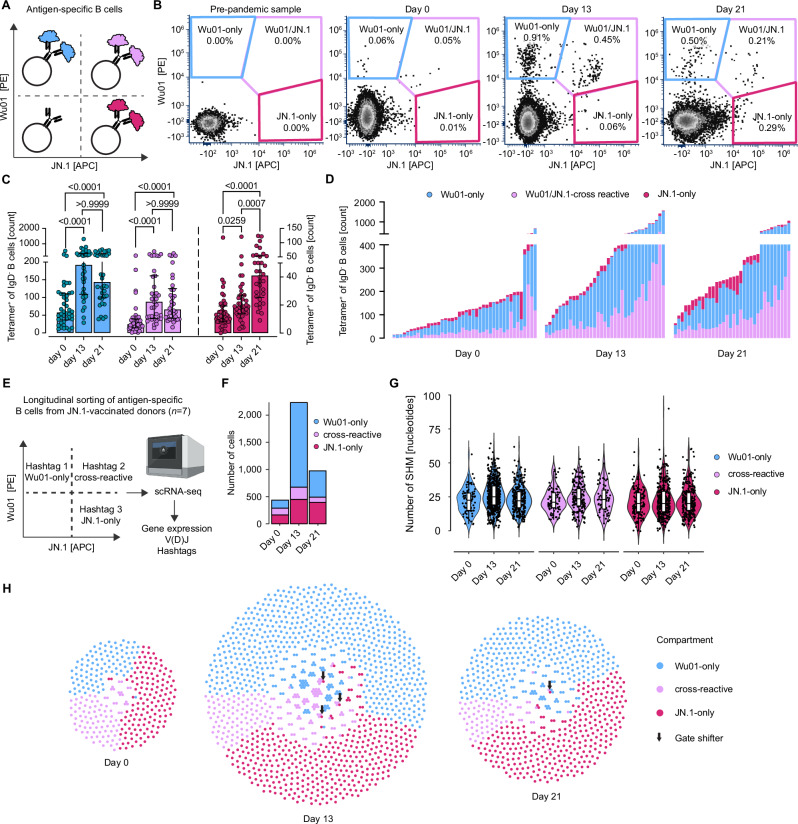
Vaccination with the JN.1-adapted mRNA booster vaccine shifts the B cell reactivity profile towards the JN.1 variant. **A** Conceptual scheme of the B cell baiting with ancestral Wu01 and JN.1 antigens labeled with two fluorescent dyes. **B** Representative gating and threshold definition for dual-bait labeling. The pre-pandemic PBMC sample (left) was used to set background-based Wu01 and JN.1 positivity thresholds. Representative dot plots from the same donor are shown. **C** Antigen-specific B cell frequencies as event counts combining class-switched MBC (CD19^+^ IgD^-^ CD20^+^) and plasmablasts (CD19^+^CD27^+^CD38^hi^ CD20^low/-^IgD^-^) within the IgD^-^ B cell gate over time, resolved for the three antigen-reactivity gates (day 0 *n* = 42, day 13 *n* = 35, day 21 *n* = 34). Data are shown as scatter dot plots; horizontal lines indicate medians, and error bars indicate interquartile range (IQR). Data derive from a single staining in which all donor samples were processed together and statistical comparisons were performed across groups; *n* indicates the number of biological replicates (individual donors), and statistical comparisons were performed across donor-level values, not across technical or experimental repeats. Statistics: Kruskal–Wallis test (two-sided) followed by Dunn’s multiple-comparisons tests (corrected for multiple comparisons) comparing all groups to each other; adjusted p-values shown in the plot. **D** Antigen-specific B cell event counts from the same pooled MBC and plasmablast compartments resolved on the donor-level (day 0 *n* = 42, day 13 *n* = 35, day 21 *n* = 34). **E** Layout of sorting and 10X Genomics scRNA-seq experiment. **F** Total number of recovered antigen-specific B cells in the scRNA-seq dataset. **G** Number of somatic hypermutations (SHM) in the heavy chain of B cells resolved for time point and the sorting gate as determined by presence of hashtag sequences. Violin plots depict the data distribution with internal boxes indicating the 25th and 75th percentiles and the central line indicating the mean. Tukey whiskers extend to the smallest and largest values within 1.5× IQR of the lower and upper quartiles, respectively. Dots represent individual cells (*n* = 82, 572, 302, 84, 182, 68, 132, 355 and 272 from left to right). No statistical test performed. **H** Honeycomb plot depicting clonotype analysis color-coded for the sorting gate. Exemplary clonotypes with mixed original sorting gates (gate shifter) are indicated with black arrows. Icons in (**A** and **E**) were created in BioRender. Kalinke, U. (2026) https://BioRender.com/h12c5rj.

To analyze the antigen-specific B cell response during vaccination, we established a B cell baiting procedure using biotinylated antigen multimerized on fluorescently labeled streptavidin. This approach allowed identification of antigen-specific B cells in samples of isolated PBMC from our cohort (Fig. 2A). B cells were classified into three reactivity gates: Wu01-only (Wu01 S bait⁺/JN.1 RBD bait⁻), JN.1-only (Wu01 S bait⁻/JN.1 RBD bait⁺), and Wu01/JN.1 cross-reactive (Wu01 S bait⁺/JN.1 RBD bait⁺) (Fig. 2B and Supplementary Fig. S2). To control for non-specific labeling, Wu01 and JN.1 positivity thresholds were defined using pre-pandemic PBMCs and held constant across all longitudinal samples (Fig. 2B). Event counts were generated under strictly standardized acquisition conditions, including identical input blood volumes, PBMC input numbers, resuspension volumes, and staining/acquisition settings across time points. Total acquired singlet event numbers were highly comparable across samples and conditions (Supplementary Fig. S1C). In the studied cohort, the number of antigen-specific B cells significantly increased with time after vaccination across all three antigen-reactivity compartments (Fig. 2C), demonstrating that the dual-bait staining procedure is sensitive to post-vaccination B cell expansion at the frequencies observed here. In addition to the quantitative increase in the frequency of antigen-specific B cells, we observed a qualitative shift in antigen-binding preference. This was primarily reflected by an increasing proportion of predominantly JN.1-only B cells, which continuously increased in proportion, particularly until day 21 post-vaccination. In contrast, the Wu01-only and Wu01/JN.1 cross-reactive populations stabilized after day 13 and did not increase further. The effect was very consistent across the analyzed cohort participants (Fig. 2D). This observation motivated our subsequent single-cell analyses to determine whether clonal maturation and SHM within pre-existing MBC clonotypes are consistent with the emergence of JN.1-only cells and the disproportionate improvement in neutralization of antigenically evolved variants.

### Pre-existing, affinity-matured MBC dominated the recall response

To address whether previously Wu01/JN.1 cross-reactive MBC transitioned into JN.1-only MBC, we selected *n* = 7 participants with serum antibody titers and neutralization capacity that were representative for the cohort (Supplementary Fig. S3). We performed a scRNA-seq experiment, in which we aimed to solve the clonal relationship of different S-reactive MBC populations. The three different antigen-reactive B cell populations (Wu01-only, JN.1-only, and Wu01/JN.1 cross-reactive) were FACS-sorted separately and labeled with three multiplex hashtags before the cell populations were unified again and sequenced together (Fig. 2E). The number of antigen-reactive B cells recovered (*n* = 3641) reflected the expected distribution with a transient increase in cells from all three gates, of which the JN.1-only population was elevated most consistently (Fig. 2F). We demultiplexed the donors based on the presence of single nucleotide polymorphisms (SNPs) and matched the derived genetic profiles to whole exome sequencing (WES) data that we generated separately for each blood donor (Supplementary Fig. S4A–C). Pseudobulk analysis revealed a transient shift in principal component (PC) 1 at day 13 (Supplementary Fig. S4D). The UMAP clustering resolved four distinct cell clusters, whereas the donors were distributed evenly, indicating the absence of donor-specific batch effects (Supplementary Fig. S4E, F). The relative contribution of each cluster did not differ between the Wu01-only, JN.1-only, and Wu01/JN.1 cross-reactive MBC, nor did it change over time (Supplementary Fig. S4G). When the differentially expressed genes (DEG) were analyzed for each cluster, only few genes were up- or downregulated (Supplementary Fig. S4H). We concluded that the MBC responded to vaccination in terms of cell numbers, while their transcriptional profiles were not strongly altered.

An increase in JN.1-only B cells after JN.1-adapted vaccination could be explained either by de novo induction of newly recruited B cell clones against JN.1 or by a qualitative shift of pre-existing B cells, e.g., via induction of SHM. We examined the SHM profile of the cells from the three sorting gates Wu01-only, JN.1-only, and Wu01/JN.1 cross-reactive and all time points. Although the JN.1-only MBC exhibited slightly lower SHM loads than Wu01-only and Wu01/JN.1 cross-reactive MBC, there was no clear enrichment of near-germline sequences over time, as would be expected if large numbers of newly recruited, low-mutated clones entered the response. Instead, the MBC slightly increased in SHM (Fig. 2G). Furthermore, testing of pooled sera did not show a noticeable induction of IgM during JN.1-adapted booster vaccination, while IgG was strongly enhanced (Supplementary Fig. S3D). Taken together, these data support a recall-dominated response and argue against substantial recruitment of near-germline, naïve-derived B cell clones in this cohort. The observed shift of certain MBC towards JN.1 may be a function of SHM in a subset of clones, consistent with progressive adaptation of their specificity towards the JN.1 variant. To visualize such an effect, if present, we identified expanded B cell clonotypes and plotted their antigen-reactivity assignment based on sample hashtags. Multiple instances of clonotypes were found, in which at least one cell mapped to the JN.1-only gate, while the remainder originated from the Wu01-only or Wu01/JN.1 cross-reactive gates. We termed such MBC clones “gate shifter”, since they appeared to have shifted from the Wu01/JN.1 cross-reactive into the JN.1-only gate as determined by flow cytometry, effectively adapting towards the JN.1 antigen by SHM (Fig. 2H). Reconstruction of pseudotime from spliced and unspliced transcripts revealed that these gate-shifting clones were in a progressed state of latent time, consistent with their occurrence through a differentiation process (Supplementary Fig. S4I).

### MBC exhibit intra-clonotype differences in binding breadth

**Fig. 3:**
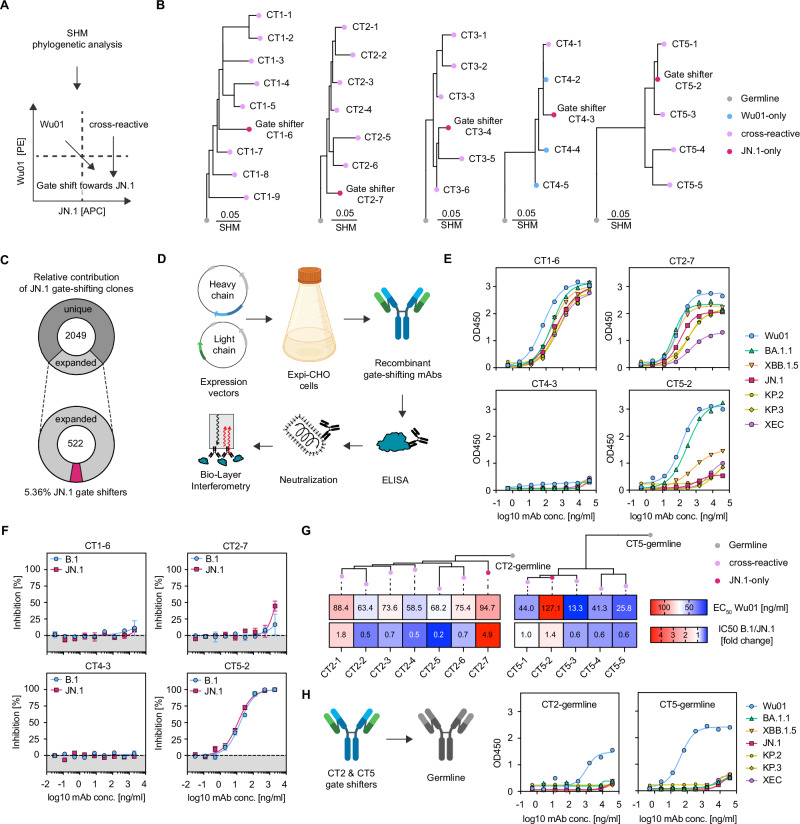
Somatic hypermutation drives memory B cell gate shift towards reactivity against the JN.1 variant. **A** Schematic depiction of the hypothesis that SHM contributes to B cell baiting gate shift. **B** Phylogenetic trees of the five largest clonotypes in the scRNA-seq dataset that contain a gate-shifting clone (indicated with black arrow). **C** Quantification of expanded clonotypes among all clonotypes in the V(D)J dataset (upper chart) and contribution of gate shifters among all expanded clonotypes (lower chart). **D** Experimental workflow for expression of recombinant monoclonal antibodies (mAbs). **E** Dose–response curves of four selected gate-shifting mAbs in multi-variant RBD ELISA. Mean values of *n* = 2 independent experiments. No statistical test performed. **F** Comparison of virus-neutralization curves between ancestral (B.1) and JN.1 variant using VSV-pseudoparticles. Mean values of *n* = 3 independent experiments. Error bars indicate +/− SEM. **G** Intra-clonotype comparison of Wu01 RBD ELISA EC50 and fold change in neutralization IC50 (B.1/JN.1) for CT2 and CT5. Shown are mean values of 2 independent experiments. No statistical test performed. **H** Depiction of germline testing strategy. SHM in VH and VL were reverted and the derived germline mAbs tested by ELISA dose–response curves. Mean of *n* = 2 independent experiments. No statistical test performed. Icons in (**D**, **H**) were created in BioRender. Kalinke, U. (2026) https://BioRender.com/h12c5rj.

To demonstrate the suspected gate-shifting behavior and link the respective MBC genotype with the affinity phenotype, we performed a phylogenetic analysis of MBC from all time points and sorting gates and filtered for the presence of gate-shifting MBC (Fig. 3A). The five largest clonotypes containing at least one gate-shifting clone were designated clonotype (CT) 1 to CT5 (Fig. 3B). Only a minority of cells from expanded clonotypes exhibited gate-shifting behavior, although the actual number in the human body may be higher due to the limited sampling depth in peripheral blood (Fig. 3C, per-donor numbers in Supplementary Fig. S4J). We expressed monoclonal antibodies (mAbs) of all 32 clones from the five largest gate-shifting clonotypes and investigated their ELISA binding, neutralization potency as well as their affinity using bio-layer interferometry (BLI) (Fig. 3D). All derived mAbs, except for the clones of CT3, bound the SARS-CoV-2 spike and S1 protein in an ELISA-based epitope screening assay (Supplementary Fig. S5A). When the more sensitive BLI method was applied to CT3, several clones such as the gate-shifting mAb CT3-4 exhibited low-affinity binding to the JN.1 RBD, validating the specificity of this ELISA-negative clonotype (Supplementary Fig. S5B). For the RBD, which constitutes a subset of the S1 domain, CT4 did not exhibit any binding, presumably because it was unable to bind to RBD alone in the absence of other non-RBD domains (Supplementary Fig. S5A). We applied the multi-variant RBD ELISA (Fig. 1D) for all mAbs (except CT3) to establish dose–responsecurves. The first gate-shifting mAb CT1-6 broadly bound all virus variants tested, the second gate-shifting mAb, CT2-7, exhibited a gradual loss of binding towards the spike of newer virus variants, the fourth gate-shifting mAb CT4-3 did not bind the RBD at all (as expected from Supplementary Fig. S5A), and the fifth gate-shifting mAb CT5-2 bound the Wu01 and BA.1.1 RBD very strongly, with a noticeable loss of binding to XBB.1.5 and JN.1, and only residual binding to KP.2, KP.3 and XEC (Fig. 3E).

Analysis of all 32 mAbs in a virus-variant neutralization assay revealed that although they bound strongly in the ELISA, the mAbs in CT1 and CT4 were non-neutralizing. Nevertheless, mAbs of CT2 neutralized weakly and CT5 neutralized SARS-CoV-2 very efficiently (Fig. 3F). Importantly, both gate-shifting mAbs from CT2 and CT5 neutralized the JN.1 variant stronger than the ancestral B.1 pseudovirus (Fig. 3F). We compared the ELISA binding strength to the Wu01 RBD as well as the change in neutralization (ratio of IC50 of B.1/JN.1; reflecting the degree of JN.1-biased neutralization for a given mAb) between the gate-shifting and their non-gate-shifting clonotype members. Both layers of evidence indicated that the gate-shifting mAbs showed weaker ELISA binding towards the Wu01 RBD than the non-gate-shifting clonotype members, while simultaneously the gate-shifting mAbs neutralized B.1 less efficiently than JN.1 (Fig. 3G). Although individual pairwise differences within clonotypes were modest, the directional pattern was consistent across orthogonal readouts, with reduced ancestral (Wu01) binding accompanied by JN.1-biased neutralization, supporting SHM-driven functional specialization of gate-shifting clones. Importantly, when germline versions of CT2 and CT5 were tested, those reacted exclusively with the Wu01 RBD in ELISA, confirming that these clonotypes were originally selected by immunization against the ancestral antigen and gained breadth only through SHM (Fig. 3H). Thus, they had effectively become adapted towards the JN.1 variant, explaining why only the gate-shifting MBC clones were baited in the JN.1-only gate. Although the loss of affinity for the Wu01 RBD was modest, the gate shifter CT5-2 exhibited an association constant kₐ that was faster towards JN.1 than to Wu01, and also had the fastest association to JN.1 in comparison to the other clones within CT5, supporting the hypothesis that the corresponding MBC was labeled brighter with the JN.1 tetramer than with the Wu01 tetramer (Supplementary Fig. S5C and Supplementary Data 1).

### SHM updated the neutralization profile of IGHV1-58 public clonotype

**Fig. 4:**
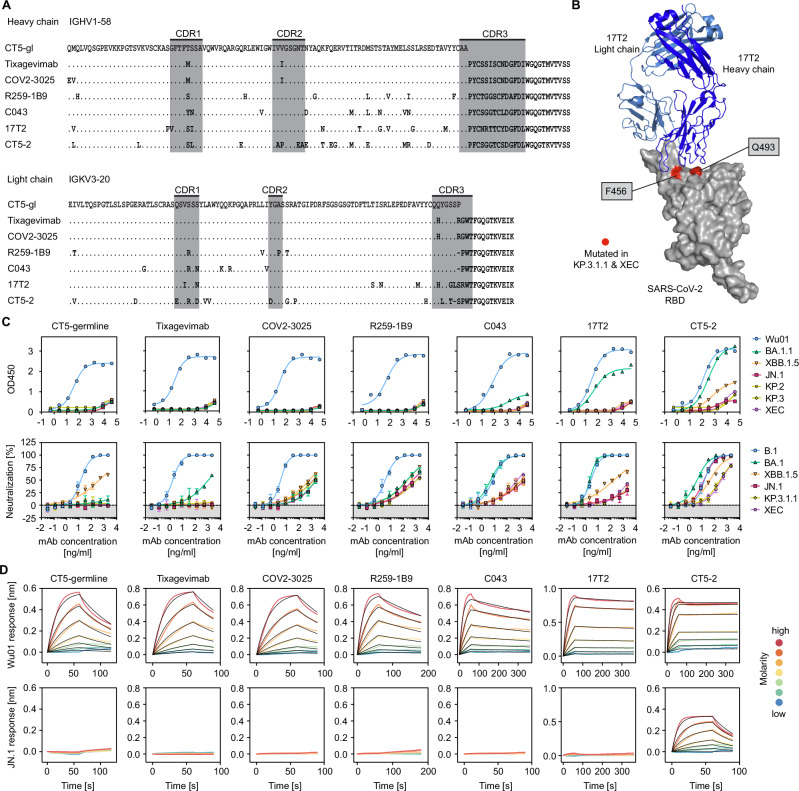
Affinity maturation of the IGHV1-58 gene segment results in progressive co-evolution between SARS-CoV-2 and human memory B cells. **A** Alignment of the gate-shifting mAb CT5-2 V(D)J sequences with related sequences of the public IGHV1-58 clonotype described in earlier publications. The sequences were ordered from top to bottom ascendingly according to the number of SHM in the heavy chain. **B** Three-dimensional visualization of the IGHV1-58 clonotype binding mode to the SARS-CoV-2 RBD (PDB 8C89). The spike residues F456 and Q493 are highlighted in red and carry mutations in the variants KP.3.1.1 and XEC. **C** ELISA binding (upper panel) and VSV pseudoparticle neutralization (lower panel) dose–response curves of seven mAbs, including the CT5-2 gate-shifting mAb described in this study. Mean values of *n* = 2 (ELISA) and *n* = 3 (virus neutralization) independent experiments. No statistical test performed for the ELISA panel (*n* = 2). Error bars for the neutralization panel indicate +/− SEM. **D** BLI affinity determination against the Wu01 RBD (upper panel) and the JN.1 RBD (lower panel). Association time: 60 seconds, dissociation time 300 seconds, shortened for optimal fit. One of *n* = 2 independent experiments shown. The binding constants are shown in Supplementary Data 1.

Interestingly, an IGHV1-58-based clonotype that was similar to CT5 was recently reported to have gained affinity for the BA.1 variant by SHM, whereas upon reversion of SHM to germline selected mAbs showed reduced neutralization breadth^23^. We expressed a germline version of CT5-2 and other related antibodies, including Tixagevimab, which was in clinical use^24^^,^ COV2-3025^25^, R259-1B9^26^, C043^27^ and the recently described pan-sarbecovirus neutralizing mAb 17T2^28^. Our gate-shifting mAb, CT5-2 carried the most SHM in both its heavy and light chains compared with the other mAbs (Fig. 4A). The molecular structure of 17T2 enabled us to visualize the presumed epitope of this public IGHV1-58 clonotype and we discovered that the positions F456 and Q493, at which mutations were newly gained in KP.3.1.1 and XEC, were located centrally beneath the heavy chain (Fig. 4B). When comparing all mAbs regarding their number of SHM, starting with the mAb CT5-germline and ending with CT5-2, it became apparent that, as previously reported, binding and neutralization of BA.1 increases with progressive SHM. Notably, CT5-2 is the only IGHV1-58 mAb with measurable ELISA binding to XBB.1.5, JN.1, KP.2, KP.3, and XEC. Simultaneously, CT5-2 neutralized JN.1 and XBB.1.5, KP.3.1.1 and XEC with high potency (Fig. 4C). To validate our findings, we compared the same set of mAbs using BLI for their affinity to the Wu01 RBD and the JN.1 RBD. The mAbs were immobilized, while the monomeric antigen was used as analyte, thus effectively ruling out avidity effects. The more SHM were included into the clonotype, the higher the affinity against Wu01 became, with CT5-2 having the highest affinity and the lowest off-rate. While CT5-germline and all mAbs until 17T2 showed no binding to JN.1, CT5-2 bound JN.1 strongly and with enhanced on-rate, although its off-rate was faster for JN.1 than for Wu01 (Fig. 4D and Supplementary Data 1). In conclusion, the SHM of the IGHV1-58 clonotype CT5 identified in this study sheds light on an ongoing B cell evolution occurring in parallel with viral evolution during the pandemic.

## Discussion

The profound loss of serum antibody binding to the SARS-CoV-2 variants JN.1, KP.3 and XEC aligns with their rapid global spread throughout 2024^29,30^. Emerging virus variants have increasingly exhibited the ability to evade host antibody responses induced by vaccination and prior infection with earlier virus variants^23^. To sustain protective immunity against COVID-19, strategies to enhance vaccine-induced SARS-CoV-2 antibody responses, particularly against such immune escape variants, are urgently needed. One proposed approach is to update the formulation of the COVID-19 vaccine by incorporating the key point mutations found in circulating virus variants. However, it remains poorly understood to what extent this strategy promotes the generation of antibody responses targeting previously unrecognized epitopes that are different from those of the ancestral virus^15^. While variant-adapted vaccines are widely assumed to predominantly recall pre-existing memory B cells, it has remained unclear to what extent and through which mechanisms such recall responses can be qualitatively reshaped to recognize newly emerged epitopes distinct from those of ancestral strains.

We observed an effective boosting in serum antibody levels after administration of the JN.1-adapted mRNA booster vaccine Comirnaty JN.1. When binding boosts were expressed as absolute increases in ED50 titers, the largest gains were observed for the earlier variants Wu01, BA.1.1, and XBB.1.5, whereas absolute increases were significantly smaller for JN.1 and JN.1-lineage descendant variants. This pattern is consistent with imprinting-dominated recall of abundant pre-existing memory specificities. Together, these data indicate that in highly pre-exposed populations, ELISA-based binding titers alone may underestimate functionally relevant refocusing within an antigenically evolved space. Importantly, vaccination was associated with serum antibodies that specifically neutralized the JN.1 variant as well as subsequently emerging variants, including KP.3.1.1 and XEC. Consistent with prior observations for the XBB.1.5-adapted mRNA booster vaccination, neutralization was highest against the variant used for the vaccine adaptation^6^. In line with our observation reported here, exposure to JN.1, which evolved from BA.2.86 under immune pressure, has recently been described to elicit particularly strong antibody responses against its subvariants^7,8,29^.

To investigate the qualitative changes in antibody response upon JN.1-adapted vaccination, we performed scRNA-seq of MBC, followed by mAb expression and multi-variant ELISA and neutralization measurements of these mAbs. This approach provided an additional layer of evidence that SHM in pre-existing MBC is, at least in part, associated with the improved neutralization observed following JN.1-adapted booster vaccination. Our “gate-shifting” clone identification approach reliably pinpoints the SHM responsible for this effect and represents an analytical tool for future studies. Within persistent clonotypes, we observed intra-clonotype specialization in which clonally related antibodies diverged functionally, and gate-shifting mAbs exhibited improved JN.1 binding and neutralization with reciprocal loss of ancestral Wu01 reactivity. Consistent with our findings, the emergence of variant-skewed and ancestral virus-skewed B cell clones within the same clonotype has recently been reported in individuals immunized with ancestral-strain-based mRNA vaccines and subsequently infected with Omicron BA.5^31^. Although contributions from prior breakthrough infections cannot be excluded, our observational dataset was acquired during vaccination and is therefore consistent with directional refinement of pre-existing MBC clonotypes following JN.1-adapted booster vaccination. Together with previous reports, our results indicate that imprinting of MBC can be overcome by SHM in a stepwise process. This co-evolution between MBC and an evolving virus may require multiple re-exposures rather than being induced by a single variant-adapted vaccination^29^. Based on our previous studies and our findings presented here, we propose that anticipatory maturation in the aftermath of an immunization can re-diversify the oligoclonal pool of immunodominant MBC clonotypes^18,19^. Upon re-encounter with a mutated antigen that evolved to escape from immunodominant antibodies, those MBC that stochastically developed SHM compatible with binding such immune escape mutants^19^ are positively selected, and either secrete their improved antibody as plasmablasts and/or undergo additional affinity maturation in secondary germinal centers. These observations support iterative vaccine updating in step with viral evolution, as successive variant-adapted boosters may cumulatively redirect pre-existing MBC toward currently circulating variants, although formal assessment of this strategy would require integration of T cell responses, clinical endpoints, and correlates of protection that were not determined in the present study. Importantly, SHM-associated repertoire refinement remained detectable even in highly pre-exposed individuals with multiple rounds of ancestral-strain vaccination, suggesting that some degree of clonotype plasticity may be retained at the B cell level following exposure to variant-updated immunogens. Consistent with a recall-dominated response, SHM loads were high and stable across time points without enrichment of near-germline sequences, and IgM responses to JN.1 and recent variants were minimal and unchanged after vaccination, arguing against substantial recruitment of naïve B cells. However, we cannot exclude contributions from newly recruited clones that rapidly class-switch, and the absence of detectable serum IgM induction is not a sensitive measure to rule out any naïve B cell participation.

Monoclonal antibodies were among the first antiviral tools deployed against SARS-CoV-2, but the emergence of resistant variants rendered most of them ineffective^32^. Tixagevimab was one such mAb used clinically and, in combination with Cilgavimab, reduced the risk of COVID-19 infection by 76.7%^24^. However, Tixagevimab failed to neutralize the Omicron BA.1 lineage, prompting efforts to adapt it to BA.1 by introducing SHM found in BA.1-neutralizing antibodies from the same IGHV1-58 clonal family^23^. In our study, we confirmed Tixagevimab’s inability to bind or neutralize any of the tested Omicron sublineages. Other IGHV1-58-based mAbs tested in this study also performed poorly against JN.1, KP.3.1.1, and XEC. In contrast, SHM identified in the mAb CT5-2 effectively adapted the antibody to JN.1 and enhanced cross-neutralization of KP.3.1.1 and XEC. These findings highlight the ongoing co-evolution of MBC initially primed by the ancestral SARS-CoV-2 strain, which can only neutralize emerging virus variants once they acquire the appropriate SHM, likely through a process of anticipatory maturation^18,19^. Our data may therefore inform future design of monoclonal antibody therapeutics to accommodate antigenic drift. The gate-shifting SHM identified here conferred neutralization breadth across multiple JN.1-lineage variants and may serve as a template for engineering next-generation broad-spectrum antibodies with resilience to viral escape.

### Limitations

We cannot definitively determine whether the observed gate shifting was induced by the JN.1-adapted booster vaccination or reflects pre-existing anticipatory maturation generated by prior exposures. Notably, gate-shifting clones were detected only after vaccination, consistent with positive selection during or after the JN.1-specific recall response, although we cannot exclude that some of the underlying SHM diversity was generated before boosting. Moreover, most participants in our study had received previous variant-adapted mRNA vaccines and/or experienced at least one breakthrough infection with Omicron subvariants. Due to this heterogeneous cohort, we cannot exclude the influence of such exposures on MBC maturation. Furthermore, as participants were healthcare workers who volunteered to enroll in the parent CoCo Study, our cohort may be subject to self-selection bias and is not necessarily representative of the general population; healthcare workers may, for example, have higher vaccine uptake, greater occupational exposure to SARS-CoV-2, and different health-seeking behavior than the broader population, which could limit the generalizability of our findings.

Although the cohort heterogeneity could be considered as design constraint, this was largely unavoidable given the aim of our study: to assess immune responses after Omicron variant-adapted vaccination. By the time Comirnaty JN.1 was authorized by the EMA in July 2024, more than four years into the pandemic, individuals had highly diverse immune histories shaped by repeated infections, exposure to different viral variants, and vaccination. Thus, establishing a comparator cohort with minimal prior exposure was practically impossible. Moreover, an earlier study time point would not have addressed our research question. Therefore, our central question, whether a JN.1-adapted booster refocuses memory B cell responses within the JN.1 antigenic space, only became biologically meaningful once this antigenically distinct vaccine was available. Prospective studies in antigen-naïve individuals and/or controlled prime-boost schedules with defined infection status and variant exposures will be needed to disentangle the relative contributions of vaccination and infection to gate-shifting and SHM-driven repertoire refinement. Nevertheless, all gate-shifting mAbs we characterized were absent prior to vaccination and emerged only after administration of the JN.1-adapted booster vaccine. Absolute B cell counts per blood volume were not obtained; instead, we relied on normalized event counts under standardized acquisition conditions, and we furthermore analyzed the frequency of antigen-specific B cells as percentage of all B cells (Supplementary Fig. S1D, E), yielding very consistent results.

Some mAbs identified in our screen showed no reactivity in ELISA assays and failed to neutralize the virus in vitro, despite being isolated from antigen-reactive MBC. In our experience, such outcomes are common and likely reflect either low-affinity interactions or reliance on conformational epitopes that are not fully preserved under in vitro conditions, and we used bio-layer interferometry (BLI) to verify such low-affinity interactions. In these cases, MBC may still be labeled by recombinant baits due to avidity effects, where multimerized biotinylated antigen facilitates cross-linking of multiple BCR on a single cell. One apparent contradiction in our data is that CT5-2 binds JN.1 considerably weaker than it does bind the Wu01 RBD, with lower saturation levels in ELISA and faster off-rate in BLI, while its neutralization potency is not affected. We speculate that while the fast off-rate reduces wash-resistance during ELISA, the fast on-rate is responsible for its strong neutralization. Future studies need to address whether binding or neutralization is the more informative readout to assess vaccine efficacy and correlates of protection. T cell responses, which also contribute to protection against SARS-CoV-2, were not assessed in this study. Future work integrating paired B cell and T cell analyses following JN.1-adapted booster vaccination would provide a more comprehensive characterization of the immune response to variant-adapted immunogens.

In conclusion, our results add another layer of information to recent findings suggesting the absence of type-specific B cells directed against Delta and BA.1^16^. In the context of JN.1, we provide evidence that such variant-specific B cells can arise from pre-existing MBC and be reshaped through SHM to recognize and neutralize emerging SARS-CoV-2 variants. Overall, our findings indicate that JN.1-adapted booster vaccination is associated with directional refinement of pre-existing memory B cell repertoires within the JN.1 antigenic space and, consequently, an improved neutralization of JN.1 by single “gate-shifting” antibody clones that utilize SHM to recognize virus variants.

## Methods

### Study design

This study was approved by the Institutional Review Board (Ethics Committee) of Hannover Medical School (approval no. 8973_BO_K_2020, last amendment August 2024, and approval no. 11475_4-BO_S_2024) and was conducted in accordance with the Declaration of Helsinki. The study is registered with the German Clinical Trial Registry (DRKS00021152). Written informed consent was obtained from all participants, who received no compensation for participation. Individuals (*n* = 46) from the COVID-19 Contact (CoCo) Study vaccinated with Comirnaty JN.1 were recruited for the study. We analyzed individuals for whom day 0 (*n* = 42), day 13 (*n* = 42) and day 21 (*n* = 39) follow-up data were available and who reported no SARS-CoV-2 infection between vaccination and day 21. The CoCo Study represents an ongoing, prospective, observational study monitoring anti-SARS-CoV-2 IgG and immune responses in healthcare professionals at Hannover Medical School^22^. While the CoCo Study cohort is not expected to present specific pre-existing conditions, twenty-seven percent of the vaccinated participants reported underlying conditions (e.g. asthma), and three of them reported treatment with methotrexate, ixekizumab plus sulfasalazine, or upadacitinib. No individual developed positive anti-NCP IgG after vaccination. Demographics (sex and age), infection, and vaccination history, respectively, are depicted in Supplementary Table S1. Sex was considered in the design of the parent CoCo Study, of which this sub-study forms part. Sex at birth was self-reported by participants. Given the near-even sex distribution within this cohort (Supplementary Table S1) and the limited sample size, no formal sex-disaggregated analysis was performed. This is an observational cohort study nested within the parent CoCo Study; participants were healthcare workers who voluntarily enrolled and received vaccination according to institutional/national recommendations, not by experimental allocation. There were no experimental groups requiring randomization. Investigators were blinded to individual participant data, but not to group allocation; baseline group-level measurements, including ELISA analyses, were performed early and in a single run to minimize variability, determine appropriate sample dilutions for subsequent time points, and confirm that the seven donors selected for longitudinal B cell analysis were representative of the entire cohort.

CoCo Study participants undergoing COVID-19 vaccination with Comirnaty JN.1 as part of the German vaccination campaign in August 2024, were invited to donate blood before (day 0) and after vaccination (days 13 and 21), which are time points at which robust antibody responses are expected^6^. At that time, KP.2, KP.3 KP.3.1.1, XEC and JN.1 were reported to be among the most detected variants according to the German wastewater-based surveillance on SARS-CoV-2 (as referred to in refs. ^7,8^).

According to our estimation, the sample size of *n* = 42 is an adequate number to detect a clinically meaningful difference within the group, assuming that S protein-reactive IgG levels double after vaccination (before: mean 822 RU/mL [SD 747]; after: mean 1644 RU/mL [SD 1494]). The estimation is grounded on anti-S IgG measurements in a convenience sample of 24 persons from the CoCo cohort in August 2023, which is our best estimate of pre-vaccination levels, a correlation between groups of 0.5 and on a one-tailed paired *t*-test of differences between means, with 95% power and 1% level of significance. Based on our prior experiences, we expected a loss-to follow-up rate of around 10%. Hence, a sample size of 46 vaccinated persons was intended. The power calculation was performed using G*Power, Version 3.1.9.6. For the present analyses, samples were available from 42 participants at day 0 and day 13, and from 39 participants at day 21. Not all available samples could be processed for every assay owing to sample availability (e.g., PBMC yield/viability), assay-specific quality control, and logistical constraints. In particular, PBMC samples for flow cytometric B cell analyses were available from *n* = 42*, n* = 35, and *n* = 34 donors at day 0, day 13, and day 21, respectively, compared with *n* = 42*, n* = 42, and *n* = 39 donors with serum availability for antibody measurements. Therefore, sample sizes vary between assays and panels, and paired analyses were restricted to participants with measurements available at both relevant time points; the exact *n* for each analysis is provided in the corresponding figure legends and Source Data.

### Serology

To quantify SARS-CoV-2-specific antibody responses, plasma was separated from EDTA or lithium heparin blood (S-Monovette, Sarstedt, Germany) after blood collection and stored at 4 °C for immediate use or at −80 °C until further use^6,23^. SARS-CoV-2 IgG was measured using quantitative ELISA according to manufacturer’s instructions with dilutions up to 1:4000 (anti-SARS-CoV-2 S1 IgG, EI 2606-9601-10G, and S1 IgG SARS-CoV-2 of Omicron, EI 2606-9601-30G, EUROIMMUN, Lübeck, Germany). Anti-NCP IgG assessment (EI 2606-9601-2G) was performed according to the manufacturer’s instructions.

For multi-variant anti-SARS-CoV-2 RBD ELISA, recombinant RBD protein of Wu01, BA.1.1, XBB.1.5, JN.1, KP.2, KP.3 and XEC (Supplementary Table S2) were dissolved in coating buffer (BioLegend, Cat # 421701) at 1 µg/ml, 100 µl per well were added to Nunc MaxiSorp ELISA plates (BioLegend, Cat # 423501), and incubated overnight at 4 °C. Plates were blocked with SuperBlock blocking buffer (Thermo Fisher, Cat# 37515) for 60 min at room temperature and washed three times with PBST (PBS supplemented with 0.25% Tween-20). Serum titration curves were generated using serial 1:5 dilutions over at least eight dilution steps in SuperBlock blocking buffer (Thermo Fisher, Cat# 37515). Variant-specific starting dilutions were empirically pre-titrated to ensure saturation and adequate dynamic range. Starting dilutions: Wu01 (1:3125), BA.1.1 (1:625), XBB.1.5 (1:125), and JN.1, KP.2, KP.3, XEC (1:5). Samples were incubated for 60 min at 37 °C, followed by three washing steps with PBST. Bound antibodies were detected with goat anti-human IgG (gamma chain) cross-adsorbed secondary antibody, HRP (Thermo Fisher, Cat# 62-8420) or goat anti-human IgM (μ-chain-specific) HRP-conjugated secondary antibody (Thermo Fisher, Cat# A18841), each diluted 1:2000 in SuperBlock (Thermo Fisher, Cat# 37515) and incubated for 60 min at room temperature followed by three washing steps. ELISAs were developed using TMB Substrate Set (BioLegend, Cat # 421101) for 30 min, reaction stopped with Stop Solution (BioLegend, Cat # 423001) and absorbance measured at 450 nm and 630 nm using a Varioskan LUX plate reader (Thermo Scientific, Germany) with SkanIt RE 6.1.1 software. Controls included blank wells, an internal reference serum, and pre-pandemic negative control sera to monitor non-specific background signal. Binding titers were quantified as ED50 values (serum dilution yielding 50% of maximal signal) by four-parameter logistic (4PL) nonlinear regression. The ELISA measurements were performed in at least four independent experiments performed on different days (and/or by different operators) starting from independently prepared antigen-coated plates and fresh reagent dilutions.

### B cell analysis

Tetramer preparation was performed using recombinant, biotinylated SARS-CoV-2 antigens (Wu01 full-length spike (S) and JN.1 RBD; Supplementary Table S3) to detect SARS-CoV-2 antigen-reactive B cells. Tetramerization was performed by incubating recombinant Wu01 S with fluorescently labeled streptavidin/R-phycoerythrin conjugate (Cat# S21388, ThermoFisher) and recombinant JN.1 RBD with fluorescently labeled streptavidin/allophycocyanin (Cat# S868; ThermoFisher)^6^. Fresh PBMCs samples were isolated, washed and resuspended in FACS buffer (PBS, 1 mg/mL BSA, 1 mmol/L EDTA). Cells were labeled with a panel of antibodies (Supplementary Table S3) and tetramerized recombinant proteins against the Wu01 S and JN.1 RBD (Supplementary Table S3) for 20 min at room temperature. Following two more washing steps, samples were acquired on a spectral flow cytometer (Cytek Northern Lights) and data analyzed using SpectroFlo and/or FCS Express software according to the gating strategy (Supplementary Fig. S2). A broad FSC-A/SSC-H gate was used to include resting as well as activated/blastoid lymphocytes (Supplementary Fig. S2). Doublets were excluded by FSC-A versus FSC-H gating, and live cells were identified using a viability dye. Myeloid cells were excluded by gating out CD14⁺ and CD16⁺ events, followed by exclusion of T cells (CD3⁺) and selection of B cells (CD19⁺). To define antigen-positivity thresholds and control for non-specific tetramer staining, pre-pandemic PBMCs were used to set background-based Wu01 and JN.1 positivity gates, which were then held constant across all longitudinal samples (Fig. 2B and Supplementary Fig. S2). To capture activated B cell subsets, antigen-specific staining was assessed in two compartments: class-switched memory B cells (CD19⁺ IgD⁻ CD20⁺) and plasmablasts (CD19⁺ CD27⁺ CD38^hi^ CD20^low/−^ IgD⁻). Tetramer binding was evaluated within each compartment using the same fixed Wu01/JN.1 tetramer gates (Supplementary Fig. S2). For quantification, antigen-binding event counts were calculated as the sum of tetramer-positive events from class-switched MBC and plasmablasts. Samples were acquired with comparable numbers of singlet events across donors and time points under standardized acquisition settings (Supplementary Fig. S1C), such that singlets served as an effective acquisition denominator without additional normalization.

### Single-cell RNA sequencing

A subset of *n* = 7 of the cohort described above was selected for single-cell RNA sequencing. The seven selected donors did not differ significantly from the remaining participants in their number of prior vaccinations (median 4 vs. 5, *p* = 0.906), number of prior infections (median 1 vs. 1, *p* = 0.358), months since last vaccination (median 10.8 vs. 10.8, *p* = 0.905) and exhibited a similar proportion of Omicron infection (85.7% vs. 86.7%, Supplementary Data 3). Fresh PBMCs samples were isolated, washed and re-suspended in FACS buffer (PBS, 1 mg/mL BSA, 1 mmol/L EDTA). MBC were isolated using Memory B Cell Isolation Kit, human (Cat # 130-093-546; Miltenyi Biotec) according to manufacturer’s protocol. Isolated MBC were then stained with antibodies and tetramerized recombinant proteins against the Wu01 S and JN.1 RBD (Supplementary Table S3) for 20 min at room temperature. Markers such as CD19 (+) and IgD (−) were incorporated in the staining panels for sorting the class-switched MBC. Following two more wash steps, samples were isolated using fluorescence-activated cell sorting (FACS) on a Becton-Dickinson FACSAria IIu (“Aria”). The Wu01-only, Wu01/JN.1 cross-reactive and JN.1-only populations were sorted into separate containers, whereas the blood donors were pooled during FACS sorting. The three antigen-reactive B cell populations were subsequently labeled for 20 min with 1 µg TotalSeq-C hashtag 1–3 antibodies (Biolegend 394661/394663/394665) in 100 µl PBS with 0.5% BSA. The labeled cells were washed 3 times before processing with 10X Genomics scRNA-seq using the Next GEM Single Cell 5’ Reagent Kit v2 (Protocol CG000330 Rev A) and sequencing on an Illumina NovaSeq X, following the manufacturer’s instructions^19^.

### Whole exome sequencing

DNA was isolated from EDTA-treated whole blood samples using the NucleoMag Blood kit (Macherey-Nagel, Düren, Germany). DNA quality was then assessed with the Qubit Fluorometer and the Qubit dsDNA BR Assay Kit (Life Technologies, Darmstadt, Germany). Whole exome sequencing (WES) was performed using the IDT Exome library kit (xGen, IDT, Leuven, Belgium). Sequencing was carried out on an Illumina NovaSeq X Plus sequencer (Illumina, San Diego, California, USA) to generate 150-bp paired-end reads from captured DNA, following the manufacturer’s protocols. An average coverage of 150 reads was achieved, with approximately 98% on-target reads.

### Generation of monoclonal antibodies

Of selected V(D)J sequences, we synthesized the variable heavy and light chain sequences and cloned them into human IgG1 heavy and kappa/lambda2 light chain expression vectors (TWIST Biosciences). These expression vectors were transfected in an equimolar ratio at a combined concentration of 1 µg/ml using the Expi-CHO Expression System (Thermo Fisher A29129) in 6-well plates with 3 ml culture volume per well, according to the manufacturer’s instructions^19^. After 10 days, the supernatants were centrifuged at 4200 × *g* for 30 min and sterile filtered. The IgG1 concentration was determined via an IgG1 ELISA (Invitrogen BMS2092) and the concentrations were normalized to a stock concentration of 20 µg/ml in DMEM (Capricorn DMEM-HXA) for neutralization testing or of 40 µg/ml in SuperBlock buffer (Thermo Scientific 37515) for ELISA measurements.

### Monoclonal antibody binding analysis

For epitope identification, the trimeric spike IgG ELISA (Invitrogen #BMS2325) and the S1 IgG ELISA (EUROIMMUN #EI 2606-9601-10G) were performed as indicated in the manufacturer’s instructions with 100 µl of mAb dilution at a fixed concentration of 2 µg/ml in the respective sample buffer of the kit.

For variant-resolved RBD binding, mAbs were tested using the same procedure as used for serum samples described above, starting the serial 1:5 dilution at a normalized mAb supernatant concentration of 40 µg/ml. Binding curves were fitted by 4PL nonlinear regression and reported as EC50 values (antibody concentration yielding 50% of maximal signal). The ELISA titrations were performed in two independent experiments, and average values were calculated before plotting.

In addition, the affinity of selected monoclonal antibodies was determined using an Octet R8e 8-channel bio-layer interferometry (BLI) system (Sartorius). The antibodies as ligands were immobilized on AHC2 biosensors (Sartorius 18-5142), while the SARS-CoV-2 RBD was used in solution as analyte. Optimal association and dissociation times were determined with loading scouts. For final comparison, similar loading of all mAbs was controlled by threshold loading of >1 nm and <2 nm loading response for all sensor tips. Analyte concentrations were titrated by 2-fold serial dilutions with highest concentration of 90.2 nM for all kinetic measurements except the low-affinity interaction of CT3-4, for which 902.3 nM was utilized. Analysis of the BLI measurements was performed using the Octet Analysis Studio 13.1.0.38. A reference sensor with the same ligand, but no analyte, was used for background signal subtraction. Data were aligned to baseline levels. Curve fitting was performed using global fit parameters, and dissociation fit was adjusted to a maximum of 300 s for stable interactions and shortened to 120, 60 or 30 s, where needed for faster dissociations. Sensorgrams were plotted using GraphPad Prism 10.6.1. The corresponding interaction constants are shown in Supplementary Data 1. All BLI measurements were performed at least twice in independent experiments, with data from one representative measurement being shown.

### Virus-neutralization assay

A pseudovirus neutralization assay (pVNT) based on a replication-restricted vesicular stomatitis virus, VSV*ΔG-FLuc (kindly provided by Gert Zimmer), was employed. Pseudovirus particles were produced in HEK-293T cells expressing codon-optimized and c-terminally truncated (18 aa residues) S proteins of SARS-CoV-2 lineages B.1 (GISAID Identifier: EPI_ISL_425259), BA.1 (GISAID Identifier: EPI_ISL_6640919), XBB.1.5 (GISAID Identifier: EPI_ISL_16239158), JN.1 (GISAID Identifier: EPI_ISL_18530042), KP.2 (GISAID Identifier: EPI_ISL_19197864), KP.3 (GISAID Identifier: EPI_ISL_19203001), KP.3.1.1 (GISAID Identifier: EPI_ISL_19455032), or XEC (GISAID Identifier: EPI_ISL_19454087). For this, cells were transfected with the respective expression plasmids by calcium-phosphate transfection and inoculated with VSV*ΔG-FLuc (multiplicity of infection = 3) at 24 h post transfection. Following 1 h of incubation, cells were washed and incubated for 24 h with medium containing VSV-G-neutralizing antibody (1:1000, I1-hybridoma cell supernatant; ATCC, cat# CRL-2700), before pseudoviruses were harvested.

pVNTs were conducted as follows. Pseudovirus particles were incubated with different dilutions/concentrations of plasma/mAb (30 min, 37 °C) before the mixtures were added to Vero cells. At 16–18 h postinoculation, efficiency of pseudovirus entry was analyzed by luciferase assay. For this, cells were lysed with PBS containing 0.5% Tergitol (Sigma-Aldrich) for 30 min at room temperature, before cell lysates were added into white 96-well plates, mixed with firefly luciferase substrate (Beetle-Juice, PJK) and luminescence was measured on a Hidex Sense plate luminometer (Hidex). For normalization, pseudovirus entry in the absence of plasma/mAb was set as reference (0% neutralization) and the relative inhibition was calculated.

### Cell lines

HEK-293T (human, female, kidney; DSMZ, cat# ACC-635, RRID:CVCL_0063) and Vero cells (African green monkey, female, kidney; ATCC, cat# CRL-1586, RRID:CVCL_0574; kindly provided by Andrea Maisner) were maintained in Dulbecco’s modified Eagle medium (PAN-Biotech), containing 10% fetal bovine serum (Biochrom) and 1% of penicillin (final concentration 100 U/ml)/streptomycin (final concentration 0.1 mg/ml of) solution (PAN-Biotech). All cell lines were cultivated at 37 °C in a humidified atmosphere enriched with 5% CO2. Cell lines were authenticated by STR analysis, partial sequencing of the cytochrome c oxidase gene and microscopic examination. Further, cell lines were regularly tested for the absence of contamination by mycoplasma using an in-house PCR assay.

### Bioinformatic analysis of single-cell RNA sequencing data

The fastq files derived from the Illumina sequencing were processed using Cell Ranger v7.1.0 using the multi command. The samples for the three different time points were processed separately, while the three B cell populations were demultiplexed based on the presence of the hashtag oligos in the feature barcode libraries. Reference sequences GRCh38-2020-A and GRCh38-alts-ensembl-7.1.0 were used for the transcriptomic and V(D)J sequence analysis, respectively. The transcriptomic data were analyzed via the Seurat package in R. Quality filters were applied for transcripts that were expressed in <5 cells, cells in which <200 genes and cells with mitochondrial gene expression >5%. Variable genes were selected via the “vst” method, and principal components analysis was performed with a default number (*n* = 50) of principal components (PC) in Seurat. The first 15 PC were selected for clustering. Unsupervised clustering of the cells was performed by the FindNeighbors and FindClusters functions of Seurat using the default Louvain algorithm and a resolution value of 0.7. The dataset was processed using the Harmony algorithm for reduction of batch effects. Non-B cells (T cells, NK cells, and monocytes) were removed. The data were reclustered with the first 10 PC and a resolution value of 0.2. Plots were generated via the unique manifold approximation and projection method. Pseudobulk analysis was performed by aggregation of the samples and PC analysis in R and per-cluster differentially expressed genes were calculated via DESeq2. The V(D)J dataset was first analyzed via the Cell Ranger “vdj” command, and the clonal expansion and isotype diversity were visualized via the 10X Genomics enclone tool (v0.5.116). Clonal lineage trees were constructed and plotted using the Dowser package (v1.1.1) and data were visualized using ggplot2 (v3.4.0), with branch length being proportional to the SHM load. The individual donors present in the dataset were identified using Souporcell^33^ and subsequently matched to vcf files derived from whole-exome sequencing of the blood donors using the Assign_Indiv_by_Geno.R script within Demuxafy^34^. To perform pseudotime analysis, aligned BAM files were processed with Velocyto (v0.17.17) to generate loom files containing spliced, unspliced, and ambiguous transcript counts for each gene in each cell. scVelo (v0.3.1) was used with the dynamical model to compute RNA velocity and predict latent time from RNA velocity dynamics. Furthermore, the single cells in the UMAP embeddings were colored by latent time and annotated by the cell barcodes of CT1, CT2, CT3, CT4 and CT5.

### Neutralizing titer calculation

Dose–response curves for experiments addressing the relative inhibition of pseudovirus entry by blood plasma or mAbs compared to samples without plasma/mAb was performed with the GraphPad Prism software (version 8.3.0 and 9.1.2, GraphPad Software) using a non-linear regression model with variable slope. Further, plasma dilutions or mAb concentrations causing half-maximal inhibition of pseudovirus entry (mAb: inhibitory concentration 50, IC50; plasma: neutralizing titer 50, NT50) were determined with thresholds of NT50 ≥ 6.25 (25% of lowest plasma dilution tested) and IC50 ≤ 5 µg/ml (2.5-times the highest mAb concentration tested) for positive reactivity, respectively.

### Antibody sequence alignments

The amino acid sequences of heavy and light chains of mAbs were aligned using Clustal Omega multiple sequence alignment on the EMBL-EBI Website (https://www.ebi.ac.uk). The visualization was subsequently prepared via manual text editing according to the alignment result in Adobe Illustrator 2024. V gene and CDR annotations were derived by alignment of nucleotide sequences using IgBlast.

### Visualization of antibody binding

The 17T2 antibody structure binding to the SARS-CoV-2 RBD antigen was fetched from the PDB database (accession number 8C89). The three-dimensional visualization was prepared using PyMOL 3.0.3 and labeled in Adobe Illustrator.

### Statistical analysis

Statistical analyses were performed using GraphPad Prism (v8.3.0, v9.1.2, v10.6.1; GraphPad Software, USA) and IBM SPSS Statistics (v20.0.0; IBM, USA). Analyses were two-sided unless stated otherwise. Unless explicitly indicated, all available biological replicates were included and no outliers were removed. Missing values were handled by analysis-specific exclusion (e.g., paired tests were restricted to donors with both time points available). Exact *p* values, multiple-comparisons-adjusted *p* values (where applicable), and the numerical values underlying all graphs are provided in the Source Data file. Biological replicates correspond to individual study participants (serology and flow cytometry) unless noted otherwise. Technical replicates refer to repeated measurements within the same assay run (e.g., replicate wells within a plate, or replicate readouts per dilution step in a neutralization run) and were combined before statistical testing. Independent experiments refer to assays performed in separate runs (e.g., different days and/or different operators), starting from independently prepared plates and fresh reagent dilutions (ELISA) or independently prepared pseudovirus/assay runs (neutralization), as specified in the figure legends. Serum binding titers were quantified as ED50 values from full titration curves using four-parameter logistic (4PL) nonlinear regression (GraphPad Prism). Monoclonal antibody binding titers were quantified analogously as EC50 values using 4PL regression. Neutralization titers are reported as NT50 values. Where geometric mean titers (GMT) are reported, titers were log-transformed and summarized on the geometric scale. Because most immunological readouts were non-normally distributed and/or paired within donors, nonparametric tests were used unless stated otherwise. Multiple comparisons were corrected using Dunn’s procedure when Friedman or Kruskal–Wallis tests were applied. Associations between baseline titers (ED50 or NT50 at day 0) and corresponding fold changes (day 21/day 0) were assessed using Spearman’s rank correlation (two-tailed). Each dot represents one participant/donor (biological replicate). Paired samples are shown as matched observations where applicable. Summary statistics (e.g., medians with IQR, or GMT where indicated) and the exact test used are stated in the corresponding figure legends, together with biological n, number of independent experiments, and technical replicate counts. Post-vaccination antibody titers (ED50 and NT50) at day 21, along with baseline titers at day 0, were log-transformed and analyzed using multivariable linear regression (ANCOVA) to assess the independent contributions of baseline titers, number of prior vaccinations, months since last vaccination, and number of prior SARS-CoV-2 infections on post-vaccination antibody levels; analyses were performed in R (version 4.4.1, R Foundation for Statistical Computing).

### Reporting summary

Further information on research design is available in the Nature Portfolio Reporting Summary linked to this article.

## Supplementary information

Supplementary Information

Description of Additional Supplementary Files

Supplementary Data 1

Supplementary Data 2

Supplementary Data 3

Reporting Summary

Transparent Peer Review file

## Source data

Source Data

## Data Availability

The raw data for the scRNA-seq experiment V(D)J and feature barcode libraries were deposited in the NCBI SRA database (project accession number PRJNA1431958). Monoclonal antibody sequences from this study were deposited in GenBank (accession numbers PZ121777–PZ121840), and are also compiled with annotated SHM in Supplementary Data 2. The de-identified numerical data underlying all figures in this study, including serological, flow cytometric, and clinical cohort measurements presented graphically, are provided in the Source Data file accompanying this manuscript. Key resources used in this study are compiled in Supplementary Table S2. Antibody expression vectors and/or proteins are available from the corresponding author upon request, subject to a material transfer agreement with Hannover Medical School. All other data are available in the article and its Supplementary files or from the corresponding author upon request. Source data are provided with this paper.
