# Supplementary material for: JN.1-adapted vaccination is associated with readjustment of ancestral memory B cells toward neutralization within the JN.1 antigenic space
Source: Nat Commun. 2026 Jul 24;17:7266. doi: 10.1038/s41467-026-76035-z (PMC13400639; doi:10.1038/s41467-026-76035-z)
Supplement: Supplementary file 1 — Supplementary Information [file 41467_2026_76035_MOESM1_ESM.pdf]

Supplementary Information

JN.1-adapted vaccination is associated with readjustment of ancestral memory B cells toward neutralization within the JN.1 antigenic space

Metodi Stankov[1,*], Matthias Bruhn[2,*], Markus Hoffmann[3], Abdus Salam[2], Amy Eichmann[3], Inga Nehlmeier[3], Luis A. Manthey[1], Torsten Witte[1], Stefan Pöhlmann[3,4], Gerrit Ahrenstorf[1], Christine Happle[1,5,6], Alexandra Dopfer-Jablonka[1], Ulrich Kalinke[2,7,8], Georg M.N. Behrens[1,7,8,9#]

[1]Department of Rheumatology and Immunology, Hannover Medical School, Hannover, Germany

[2]Institute for Experimental Infection Research, TWINCORE, Centre for Experimental and Clinical Infection Research, a joint venture between the Helmholtz Centre for Infection Research and the Hannover Medical School, Hannover, Germany

[3]Infection Biology Unit, German Primate Center – Leibniz Institute for Primate Research, Göttingen, Germany

[4]Faculty of Biology and Psychology, Georg-August-University Göttingen, Göttingen, Germany

[5]Department of Pediatric Pulmonology, Allergology and Neonatology, Hannover Medical School, Hannover, Germany

[6]Biomedical Research in Endstage and Obstructive Lung Disease Hannover (BREATH), Member of the German Center for Lung Research (DZL), Hannover, Germany.

[7]Cluster of Excellence RESIST (EXC 2155), Hannover Medical School, 30625 Hannover, Germany

[8]German Center for Infection Research (DZIF), partner site Hannover-Braunschweig, Hannover, Germany

[9]Center for Individualized Infection Medicine (CiiM), Hannover, Germany

*These authors contributed equally

[#]Corresponding author: Georg M.N. Behrens, Department of Rheumatology and Immunology, Hannover Medical School, Carl-Neuberg-Straße 1, D - 30625 Hannover, Germany, Tel: +49 511 532 5337, Fax: +49 511 532 5324, Email: Behrens.georg@mh-hannover.de

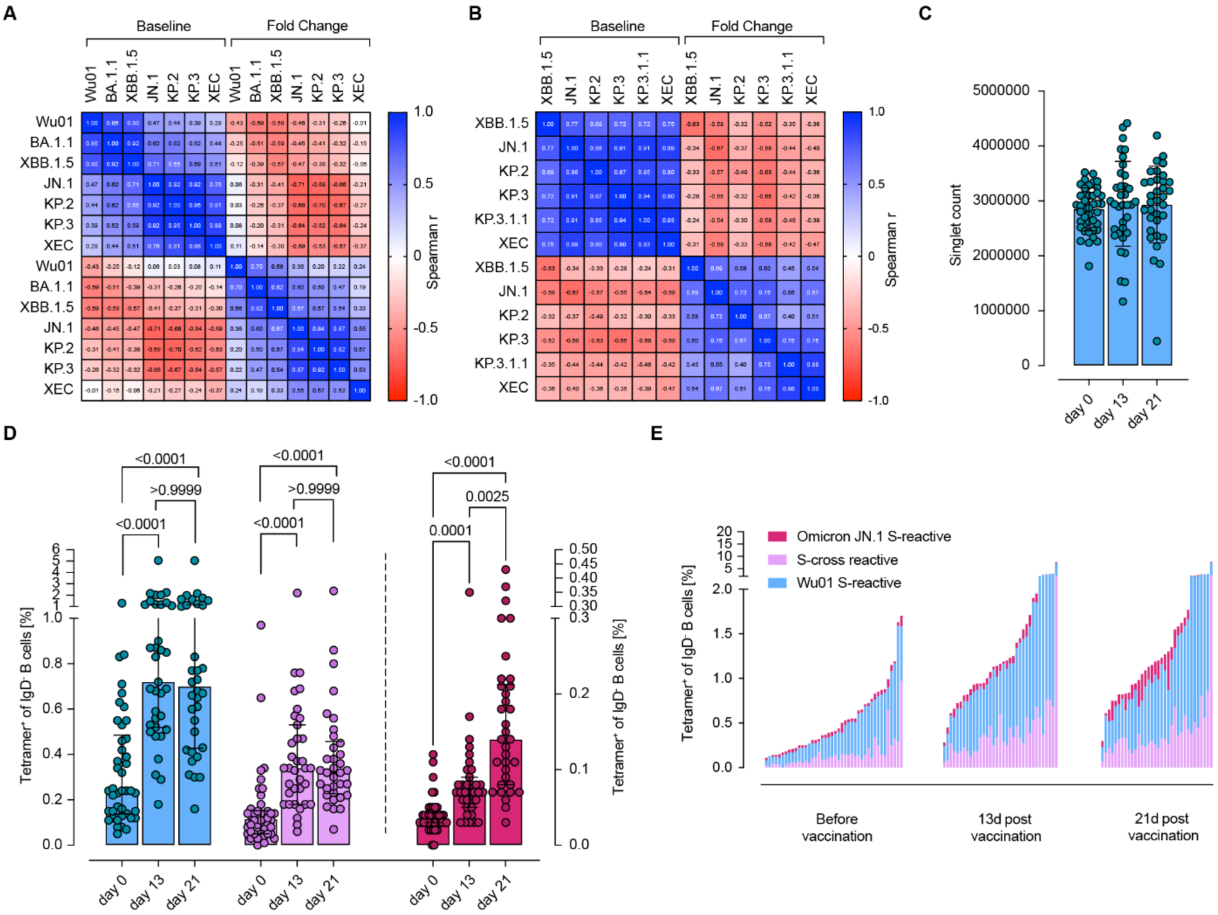

Figure S1: Baseline-boost relationships for binding and neutralization, and flow-cytometry acquisition/QC metrics. (A) Correlation matrix relating baseline serum binding (ED50 at day 0) to fold change in binding from day 0 to day 21 for each RBD variant shown in Fig. 1E–G. ELISA data were generated from 4 independent experiments (day 0 *n*=42, day 21 *n*=39). (B) Correlation matrix relating baseline neutralization (NT50 at day 0) to fold change in neutralization from day 0 to day 21 for each spike variant shown in Fig. 1I-J. Neutralization data were generated from 2 independent experiments, each performed with 4 technical replicates per dilution step (day 0 *n*=39, day 21 *n*=39). (C) Flow-cytometry acquisition/QC summary showing total singlet event counts at day 0, day 13, and day 21 for samples used in Fig. 2B-D (day 0 *n*=42, day 13 *n*=35, day 21 *n*=34). (D) Antigen-specific B cell frequencies shown as percent of the parent IgD⁻ B cell gate over time during JN.1-adapted mRNA booster vaccination, resolved for the three antigen-reactivity gates (Wu01-only, Wu01/JN.1 cross-reactive, JN.1-only) (day 0 *n*=42, day 13 *n*=35, day 21 *n*=34). (E) Donor-level distribution of antigen-specific B cell frequencies shown as percent of the parent IgD⁻ B cell gate at day 0, day 13, and day 21 (day 0 *n*=42, day 13 *n*=35, day 21 *n*=34). Flow-cytometry data in panels C-E derive from 1 independent staining/acquisition experiment with the indicated number of biological replicates. Correlation matrices in panels A-B are based on participant-level values; the corresponding ED50/NT50 and fold change data are shown in Fig. 1 and provided in the Source Data. Statistics (D): Kruskal-Wallis test with Dunn's multiple-comparisons correction. Heatmaps show pairwise correlations calculated using Spearman's rank correlation (two-tailed) across participants.

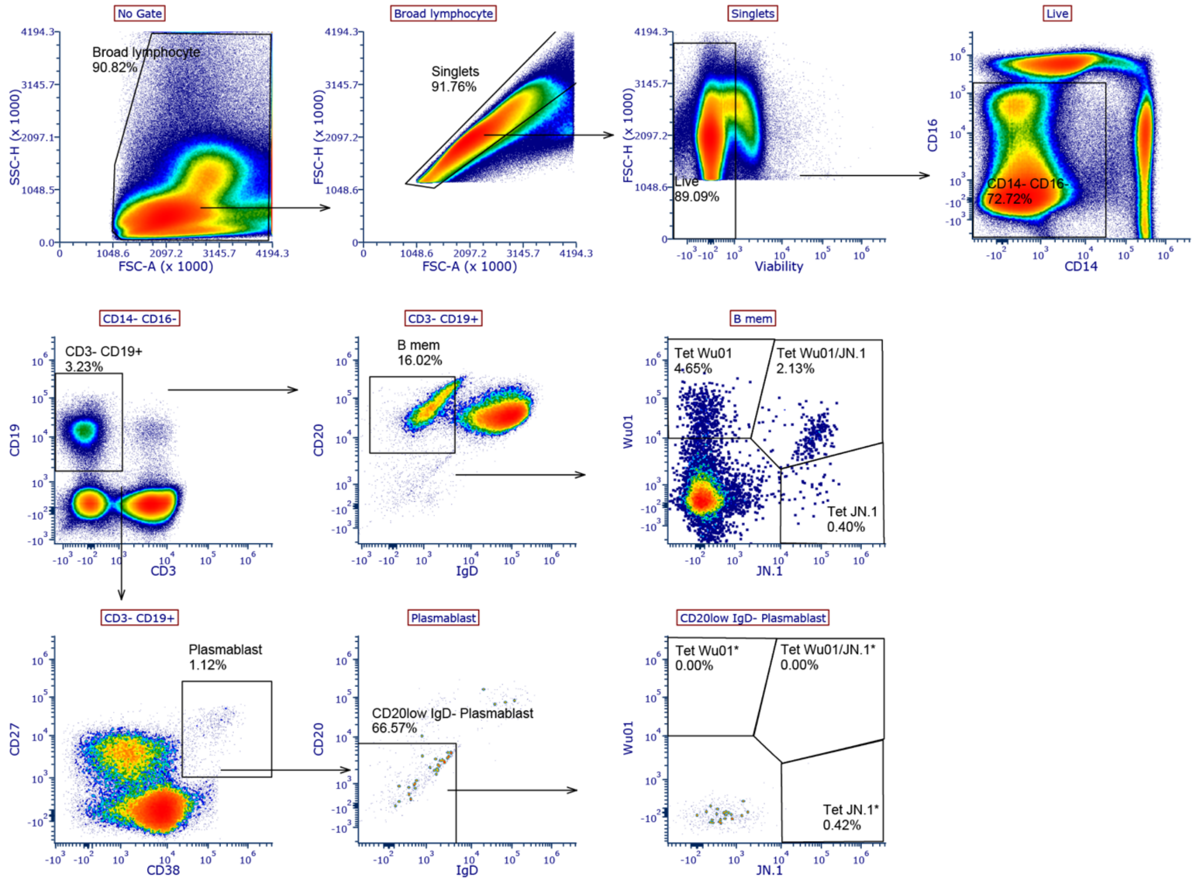

Figure S2: Gating strategy for flow cytometric identification of Wu01- and JN.1-bait-binding B cells. PBMC were isolated, immunolabeled and analyzed by flow cytometry. A broad FSC-A/SSC-H gate was used to include resting as well as activated/blastoid lymphocytes. Doublets were excluded by FSC-A versus FSC-H gating, and live cells were identified using a viability dye. Myeloid cells were excluded by gating out $CD14^{+}$ and $CD16^{+}$ events, followed by exclusion of T cells ($CD3^{+}$) and selection of B cells ($CD19^{+}$). Antigen-specific B cells were subsequently quantified by dual-tetramer staining with Wu01 (ancestral) and JN.1 baits and resolved into three reactivity gates (Wu01-only, JN.1-only, and Wu01/JN.1 cross-reactive). Tetramer thresholds were defined using pre-pandemic PBMCs and then held constant across all samples. To capture activated B cell subsets, antigen-specific staining was assessed in two compartments. Class-switched memory B cells were defined as $CD19^{+}$ IgD^{-} $CD20^{+}$ (memory B cells) and analyzed for tetramer binding (upper row), which by definition excludes $CD20^{low/-}$ plasmablasts. Plasmablasts were explicitly included and defined as $CD19^{+}$ $CD27^{+}$ CD38hi $CD20^{low/-}$ IgD^{-}, and tetramer binding was assessed within this compartment using the same fixed tetramer gates (lower row). For quantitative analyses in Fig. 2, antigen-binding cells were evaluated as the pooled population of class-switched memory B cells plus plasmablasts by summing tetramer-positive events from both branches (i.e., all $CD19^{+}$ IgD^{-} antigen-binding events captured by this gating strategy). Samples were acquired with comparable numbers of singlet events across donors and time points, such that singlets served as an effective acquisition denominator without additional normalization. Representative plots illustrate the full gating hierarchy and the final tetramer-based assignment of antigen-reactive populations. The labels in the red boxes above each plot identify the parental gate from which the displayed events were derived; the gate boundary and percentage shown within each plot indicate the population selected for the next level of analysis.

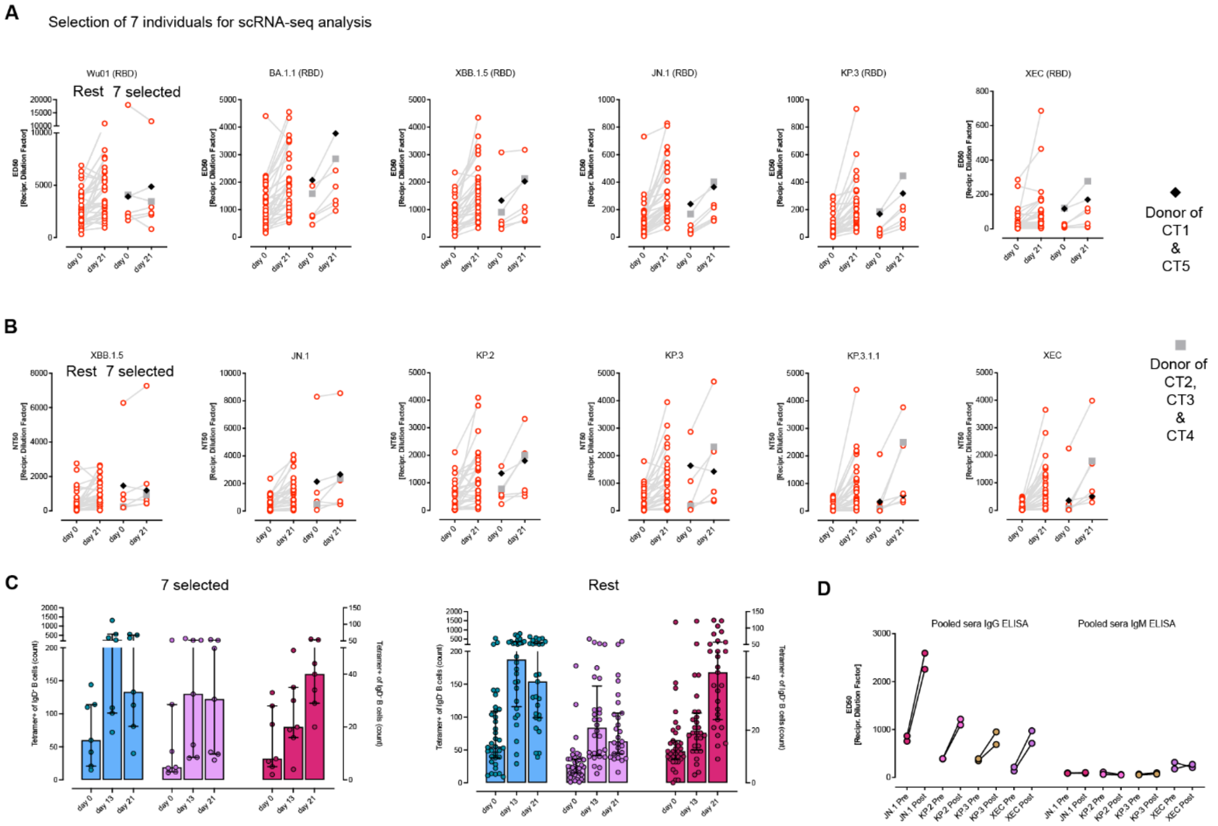

Figure S3: Selection of cohort subset (*n*=7) for B cell scRNA-seq analysis and pooled IgG/IgM ELISA. (A) Serum IgG binding to variant RBDs quantified as ED50 (50% effective dilution) derived from 4-parameter logistic fits of full titration curves, as in Fig. 1E, but shown separately for the cohort excluding the seven scRNA-seq donors (left panels; day 0 *n*=35, day 21 *n*=32, paired) and the seven scRNA-seq donors (right panels; day 0 *n*=7, day 21 *n*=7, paired) at day 0 and day 21 post booster vaccination. (B) Serum neutralization titers (NT50) against VSV pseudoviruses bearing spike proteins of the indicated variants, as in Fig. 1I, but shown separately for the cohort excluding the seven scRNA-seq donors (left panels; day 0 *n*=32, day 21 *n*=32, paired) and the seven scRNA-seq donors (right panels; day 0 *n*=7, day 21 *n*=7, paired) at day 0 and day 21 post booster vaccination. Whole exome sequencing (WES) was performed to match scRNA-seq data to donor identity. The black square indicates the donor from which monoclonal antibody (mAb) clonotypes CT1 and CT5 are derived; the grey square indicates the donor from which CT2, CT3 and CT4 are derived. (C) Antigen-specific B cell quantification of the seven donors selected for the scRNA-seq experiment vs. the remaining cohort. The left three bars correspond to Wu01-only, the middle three bars to cross-reactive and the right three bars to JN.1-only reactive B cells at three time points, respectively. (D) Pooled sera from the full cohort (day 0: *n*=42; day 21: *n*=39) were serially diluted and tested by multi-variant SARS-CoV-2 RBD ELISA for the indicated variants using anti-IgG or anti-IgM secondary antibodies. ED50 titers were determined by 4-parameter logistic curve fitting. Data in (C) are from 2 independent experiments per ELISA. Statistics: Panels (A-B) are descriptive subset displays of the participant-level data shown in Fig. 1 and are provided to illustrate representativeness of the scRNA-seq donor subset. Exact n per subgroup and all numerical values are provided in the Source Data.

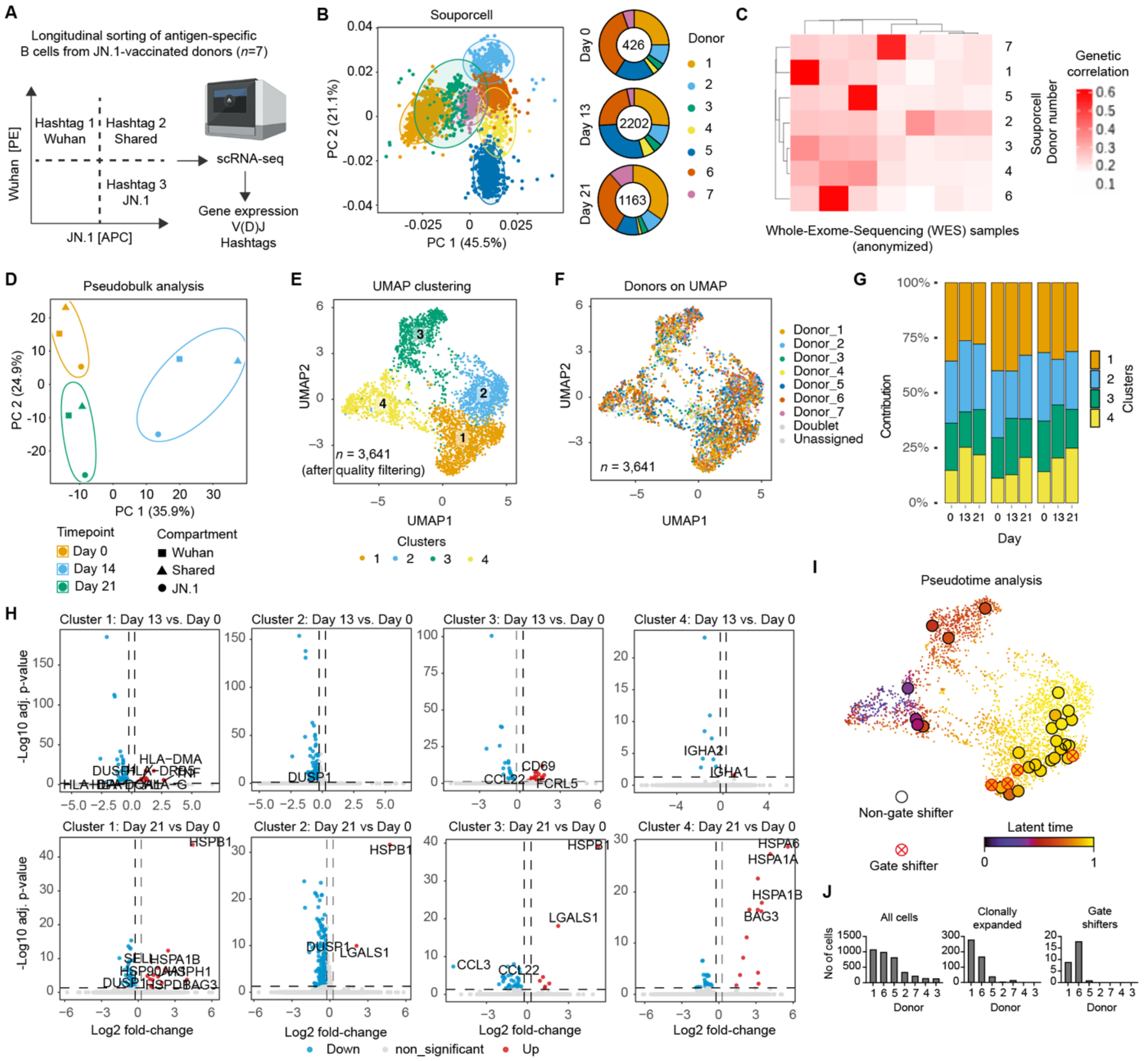

Figure S4: Bioinformatic analysis of the scRNA-seq transcriptome dataset. (A) Experimental overview. (B) Souporcell was used to demultiplex the seven donors based on the presence of SNPs. (C) The donor demultiplexing was verified using WES of the seven individuals, which were matched to the Souporcell clusters. (D) Pseudobulk analysis of the three different sorting compartments at three time points each. (E) UMAP clustering of the transcriptomes. (F) Donor identity plotted over the UMAP clusters. (G) Relative contribution of the four UMAP clusters to the different sorting compartments and time points. (H) DEG analysis of each cluster. Day 0 was utilized as baseline and the DEG of Day 13 (upper panel) and Day 21 (lower panel) are indicated. (I) Pseudotime analysis using scVelo, highlighting all cells from CT1 to CT5 that passed the transcriptomic data quality filters. (J) The number of all cells, clonally expanded B cells and gate shifters resolved for donor numbers as determined by Souporcell. Icons in Fig. S4A were created in BioRender. Kalinke, U. (2026) https://BioRender.com/h12c5rj.

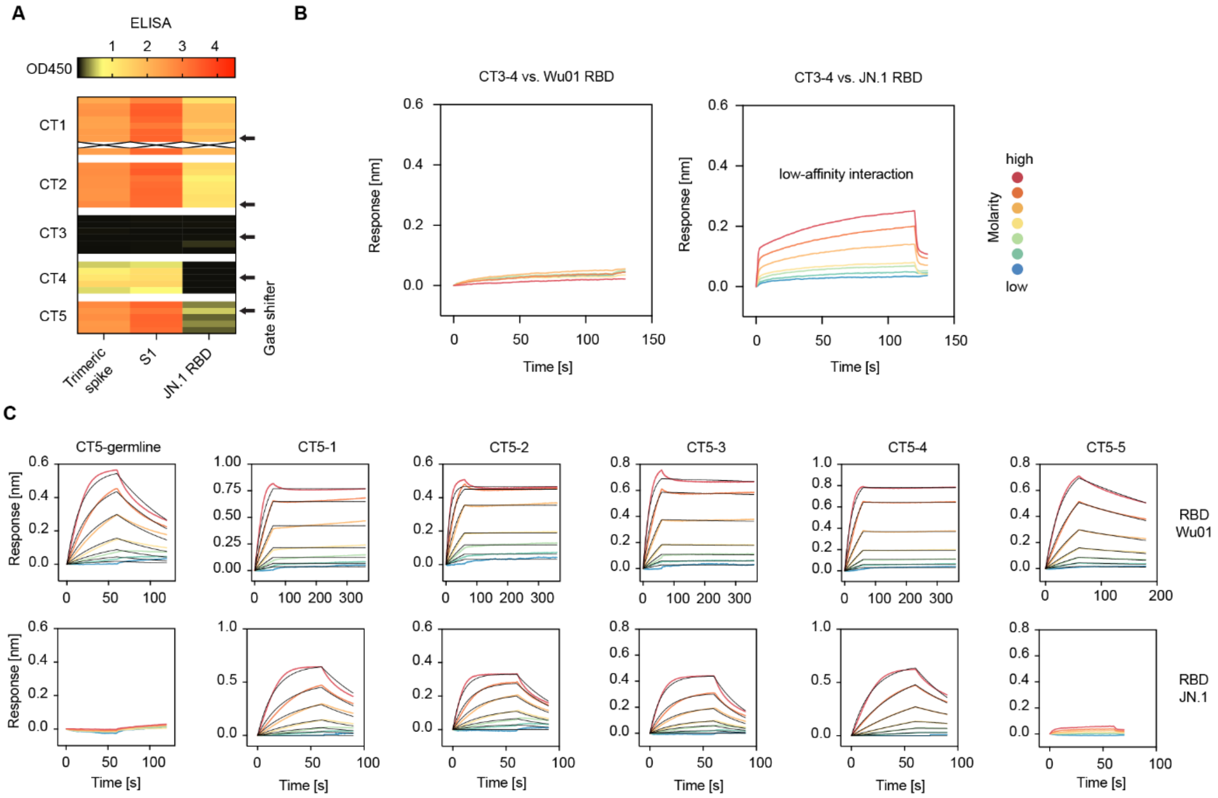

Figure S5: Specificity and affinity of selected gate-shifting mAbs. (A) ELISA epitope binding screen at mAb concentration of 2 µg/ml. (B) Low-affinity BLI measurement of ELISA-negative CT3-4 against Wu01 RBD (left side) and JN.1 RBD (right side). No curve fitting was applied due to low affinity. (C) Intra-clonotype comparison between Wu01 and JN.1 RBD using BLI for CT5 clones. No curve fitting was applied for CT5-germline (vs. JN.1) and CT5-5 (vs. JN.1) due to low affinity. The full set of corresponding binding constants is shown in Supplementary Data 1. One representative of n=2 independent experiments shown.

Table S1: Cohort description.

Variable	Vaccinees
Probands (*n*=)	42
Age, Median, [IQR] (years)	47 [20]
Sex, male %	48
Median time post last vaccination [IQR] (months)	11 [9.5]
Median number of prior vaccinations [IQR]	4.5 [1]
Prior SARS-CoV-2 Omicron vaccination (%)	36/42 (85.7)
Prior SARS-CoV-2 infection (%)*	36/40 (90.0)
Prior SARS-CoV-2 Omicron infection (%)*	34/39 (87.2)
Prior SARS-CoV-2 Omicron antigen contact (%)	41/42 (97.6)
Anti-NCP IgG positive (%)	11/42 (26.2)
Study vaccine antigen	Omicron JN.1 spike (monovalent) Bretovameran (BioNTech-Pfizer), mRNA, 30 µg

*For some study participants this information was not available

Table S2: Overview of key resources used in this study

Reagent	Source	Identifier
**Antibodies**		
Alexa Fluor 488 anti-human CD16 Antibody	Biolegend Cat# 302019	RRID: AB_492974
PE/Cyanine5 anti-human CD14	Biolegend Cat# 301864	RRID: AB_2860767
Brilliant Violet 570 anti-human CD20	BioLegend Cat# 302332	RRID: AB_2563805
APC/Fire 810 anti-human CD38	BioLegend Cat# 303550	RRID: AB_2860784
CD3 Monoclonal Antibody (UCHT1), Alexa Fluor 532, eBioscience	eBioscience Cat# 58-0038-42	RRID: AB_11218675
CD27 Monoclonal Antibody (O323), Alexa Fluor 700, eBioscience	eBioscience Cat# 56-0279-42	RRID: AB_11044789
Pacific Blue anti-human CD19	BioLegend Cat# 363036	RRID: AB_2632787
PE/Cyanine7 anti-human CD19	BioLegend Cat# 363012	RRID: AB_2564203
Brilliant Violet 480 anti-human IgD	BD Biosciences Cat# 566138	RRID: AB_2739536
Goat anti-human IgM (μ-chain chain) cross-adsorbed secondary antibody, HRP	Thermo Fisher, Cat# A18841	RRID: AB_2535606
Goat anti-Human IgG (Gamma chain) Cross-Adsorbed Secondary Antibody, HRP	Thermo Fisher, Cat# 62-8420	RRID: AB_2533962
Human IgG1 monoclonal antibodies	This study	N/A
**Bacterial and virus strains**		
VSV*ΔG-FLuc	Lab of Gert Zimmer	N/A
**Biological samples**		
Serum samples of vaccinated individuals	This study	N/A
**Chemicals, peptides, and recombinant proteins**		
Zombie NIR Fixable Viability Kit	BioLegend	#423106
Biotinylated SARS-CoV-2 Spike Trimer (Wuhan)	Acrobiosystems	#SPN-C82E9
Biotinylated SARS-CoV-2 Spike RBD Protein (JN.1/Omicron)	Acrobiosystems	#0BB1D-F001
PE-Streptavidin	BioLegend	#405203
APC-Streptavidin	BioLegend	#405207
SARS-CoV-2 (COVID-19) S protein RBD, His Tag	Acrobiosystems	#SPD-C52H3
SARS-CoV-2 Spike RBD Protein, His Tag (BA.1.1/Omicron)	Acrobiosystems	#SPD-C522j
SARS-CoV-2 Spike RBD Protein, His Tag (XBB.1.5/Omicron)	Acrobiosystems	#SPD-C5242
SARS-CoV-2 Spike RBD Protein, His Tag (JN.1/Omicron)	Acrobiosystems	#SPD-C5249
SARS-COV-2 KP.2 (Omicron) Spike RBD Protein (His Tag)	Sino Biological	#40592-V08H156
SARS-COV-2 KP.3 (Omicron) Spike RBD Protein (His Tag)	Sino Biological	#40592-V08H157
SARS-CoV-2 RBD of Spike protein - XEC	Proteogenix	#PX-COV-P096-50
**Critical commercial assays**		

Anti-SARS-CoV-2 S1 Spike protein domain/receptor binding domain IgG SARS-CoV-2-QuantiVac	EUROIMMUN	EI 2606-9601-10G
Anti-SARS-CoV-2 Omicron ELISA (IgG)	EUROIMMUN	EI 2606-9601-30 G
Anti-NCP IgG ELISA	EUROIMMUN	EI 2606-9601-2 G
Next GEM Single Cell 5' Reagent Kit v2	10X Genomics	Protocol CG000330 Rev A
Human IgG1 ELISA	Invitrogen	BMS2092
Human SARS-CoV-2 Spike (Trimer) IgG ELISA	Invitrogen	BMS2325
**Deposited data**		
Monoclonal antibody sequences	This study	GenBank PZ121777-PZ121840
V(D)J Sequencing data	This study	SRA PRJNA1431958
Hashtag Sequencing data	This study	SRA PRJNA1431958
**Cell lines**		
Vero	Lab of Andrea Maisner	ATCC cat# CRL-1586, RRID:CVCL_0574
HEK-293T	DSMZ	DSMZ cat# ACC-635, RRID:CVCL_0063
**Oligonucleotides**		
TotalSeq-C anti-human hashtag 1	BioLegend	Cat# 394661
TotalSeq-C anti-human hashtag 2	BioLegend	Cat# 394663
TotalSeq-C anti-human hashtag 3	BioLegend	Cat# 394665
**Recombinant DNA**		
Expression Vectors for recombinant mAbs	TWIST Biosciences	This study
**Software and algorithms**		
G*Power		Version 3.1.9.6
GraphPad PRISM		Version 8.3.0, 9.1.2, 10.6.1
Gen5		Version 2.01
Microsoft Excel		Version 16.96.1
Adobe Illustrator		Version 2024
Cellranger		Version 7.1.0
enclone		Version 0.5.116
Souporcell		Version 2.5
Demuxafy		3.0.0
R Studio		Version 2024.12.1+563
PyMOL	Schrodinger, LLC	Version 3.0.3
Octet Analysis Studio	Sartorius	Version 13.1.0.38
Clustal Omega	EMBL-EBI	Version 1.2.4
IgBLAST	NCBI	Version 1.22.0
R	R Foundation for Statistical Computing	Version 4.4.1
IBM SPSS Statistics	IBM Corp.	Version 20.0.0
Seurat		Version 5.3.0
Harmony		Version 1.2.4
Dowser		Version 1.1.1
ggplot2		Version 3.4.0
Velocyto		Version 0.17.17

scVelo		Version 0.3.1
DESeq2		Version 1.48.0

Table S3: Immunolabeling for Flow cytometry.

Antigen	Conjugate	Clone	Order no.	Company	Dilution
CD16	Alexa Fluor 488	3G8	302019	BioLegend	100
CD14	PE/Cy5	M5E2	301864	BioLegend	100
CD20	Brilliant Violet 570	2H7	302332	BioLegend	100
CD38	APC-Fire810	HIT2	303550	BioLegend	100
CD3	Alexa Fluor 532	UCHT1	58-0038-42	eBioscience	100
CD27	Alexa Fluor 700	O323	56-0279-42	eBioscience	100
CD19	Pacific Blue	SJ25C1	363036	BioLegend	100
CD19	PE/Cy7	SJ25C1	363012	BioLegend	100
IgD	BV480	1A6-2	566138	BD Biosciences	100
Viability	Zombie NIR	NA	423106	BioLegend	1000
Wu01 Tetramer	PE			This study	100
JN.1 Tetramer	APC			This study	400
