# Supplementary material for: JN.1-adapted vaccination is associated with readjustment of ancestral memory B cells toward neutralization within the JN.1 antigenic space
Source: Nat Commun. 2026 Jul 24;17:7266. doi: 10.1038/s41467-026-76035-z (PMC13400639; doi:10.1038/s41467-026-76035-z)
Supplement: Supplementary file 2 — Description of Additional Supplementary Files [file 41467_2026_76035_MOESM2_ESM.pdf]

Description of Additional Supplementary Files

Supplementary Data 1: BLI-derived binding constants for CT5 monoclonal antibody clones (cited in Methods and Figure 4 legend and Supp. Fig. S5 legend).

Supplementary Data 2: Annotated monoclonal antibody sequences with somatic hypermutation (SHM) annotations (cited in Data Availability statement).

Supplementary Data 3: Statistical comparison data: association between baseline and post-vaccination antibody titers, and comparison of scRNA-seq-selected donor subgroup vs. remaining cohort (cited in Results and Methods).
