# Supplementary material for: JN.1-adapted vaccination is associated with readjustment of ancestral memory B cells toward neutralization within the JN.1 antigenic space
Source: Nat Commun. 2026 Jul 24;17:7266. doi: 10.1038/s41467-026-76035-z (PMC13400639; doi:10.1038/s41467-026-76035-z)
Supplement: Supplementary file 6 — Reporting Summary [file 41467_2026_76035_MOESM6_ESM.pdf]

nature portfolio

Corresponding author(s): Georg M.N. Behrens

Last updated by author(s): Jul 6, 2026

Reporting Summary

Nature Portfolio wishes to improve the reproducibility of the work that we publish. This form provides structure for consistency and transparency in reporting. For further information on Nature Portfolio policies, see our Editorial Policies and the Editorial Policy Checklist.

Statistics

For all statistical analyses, confirm that the following items are present in the figure legend, table legend, main text, or Methods section.

n/a	Confirmed	
☐	☒	The exact sample size (*n*) for each experimental group/condition, given as a discrete number and unit of measurement
☐	☒	A statement on whether measurements were taken from distinct samples or whether the same sample was measured repeatedly
☐	☒	The statistical test(s) used AND whether they are one- or two-sided<br>*Only common tests should be described solely by name; describe more complex techniques in the Methods section.*
☐	☒	A description of all covariates tested
☐	☒	A description of any assumptions or corrections, such as tests of normality and adjustment for multiple comparisons
☐	☒	A full description of the statistical parameters including central tendency (e.g. means) or other basic estimates (e.g. regression coefficient) AND variation (e.g. standard deviation) or associated estimates of uncertainty (e.g. confidence intervals)
☐	☒	For null hypothesis testing, the test statistic (e.g. *F*, *t*, *r*) with confidence intervals, effect sizes, degrees of freedom and *P* value noted<br>*Give P values as exact values whenever suitable.*
☒	☐	For Bayesian analysis, information on the choice of priors and Markov chain Monte Carlo settings
☒	☐	For hierarchical and complex designs, identification of the appropriate level for tests and full reporting of outcomes
☐	☒	Estimates of effect sizes (e.g. Cohen's *d*, Pearson's *r*), indicating how they were calculated

Our web collection on statistics for biologists contains articles on many of the points above.

Software and code

Policy information about availability of computer code

Data collection	Flow cytometry acquisition: Cytek Northern Lights; acquisition software SpectroFlo (Methods, B cell analysis). Plate reader acquisition (ELISA): Varioskan LUX plate reader (Thermo Scientific) with SkanIt RE 6.1.1 software. Neutralization readout: Hidex Sense plate luminometer (Hidex).
Data analysis	GraphPad Prism v8.3.0, v9.1.2, v10.6.1 (GraphPad Software, USA); IBM SPSS Statistics v20.0.0 (IBM Corp.); RRID: SCR_002865; Cell Ranger v7.1.0; Seurat (R package) v5.3.0; Harmony v1.2.4; DESeq2 v1.48.0; Dowser v1.1.1; ggplot2 v3.4.0; Velocyto v0.17.17; scVelo v0.3.1; enclone v0.5.116; Souporcell v2.5; Demuxafy v3.0.0; R v4.4.1 (R Foundation for Statistical Computing); RRID: SCR_001905; R Studio v2024.12.1+563; G*Power v3.1.9.6; Gen5 v2.01; Microsoft Excel v16.96.1; Adobe Illustrator 2024; PyMOL v3.0.3 (Schrodinger, LLC); RRID: SCR_000305; Octet Analysis Studio v13.1.0.38 (Sartorius); Clustal Omega v1.2.4 (EMBL-EBI); RRID: SCR_001591; IgBLAST v1.22.0 (NCBI); RRID: SCR_002873.

For manuscripts utilizing custom algorithms or software that are central to the research but not yet described in published literature, software must be made available to editors and reviewers. We strongly encourage code deposition in a community repository (e.g. GitHub). See the Nature Portfolio guidelines for submitting code & software for further information.

Data

Policy information about availability of data

All manuscripts must include a data availability statement. This statement should provide the following information, where applicable:

- Accession codes, unique identifiers, or web links for publicly available datasets
- A description of any restrictions on data availability
- For clinical datasets or third party data, please ensure that the statement adheres to our policy

Data availability:
The raw data for the scRNA-seq experiment V(D)J and feature barcode libraries were deposited in the NCBI SRA database (project accession number PRJNA1431958) and are available without restriction at https://www.ncbi.nlm.nih.gov/bioproject/PRJNA1431958. Monoclonal antibody sequences from this study were deposited in GenBank (accession numbers PZ121777-PZ121840) and are also compiled with annotated SHM in Supplementary Data 2. The de-identified numerical data underlying all figures in this study, including serological, flow cytometric, and clinical cohort measurements presented graphically, are provided in the Source Data file accompanying this manuscript. Antibody expression vectors and/or proteins are available from the corresponding author upon request, subject to a material transfer agreement with Hannover Medical School.

Code availability:
This paper does not report original code.

Research involving human participants, their data, or biological material

Policy information about studies with human participants or human data. See also policy information about sex, gender (identity/presentation), and sexual orientation and race, ethnicity and racism.

Reporting on sex and gender	Sex was considered in the design of the parent CoCo Study, of which this sub-study forms part. Sex at birth was self-reported by participants. Given the near-even sex distribution within this cohort (48% male; Table S1) and the limited sample size, no formal sex-disaggregated analysis was performed.
Reporting on race, ethnicity, or other socially relevant groupings	Not applicable - race, ethnicity, or other socially relevant groupings were not collected or assessed in this study.
Population characteristics	n=42 participants (COVID-19 Contact [CoCo] Study, Comirnaty JN.1 vaccinees); median age 47 years [IQR 20]; 48% male; median 4.5 prior vaccinations [IQR 1]; median 11 months since last vaccination [IQR 9.5]; 90.0% prior SARS-CoV-2 infection (36/40, where available); 87.2% prior Omicron infection (34/39); 97.6% prior Omicron antigen contact (41/42); 26.2% anti-NCP IgG positive at baseline (11/42); 27% reported underlying conditions (e.g., asthma; three participants on methotrexate, ixekizumab+sulfasalazine, or upadacitinib). (Source: Table S1 + Methods)
Recruitment	Participants were recruited from the ongoing, prospective, observational COVID-19 Contact (CoCo) Study monitoring anti-SARS-CoV-2 IgG and immune responses in healthcare professionals at Hannover Medical School. Individuals vaccinated with Comirnaty JN.1 as part of the German vaccination campaign in August 2024 were invited to donate blood at day 0, 13, and 21 post-vaccination. Self-selection bias: as participants were healthcare workers who volunteered to enroll in the parent CoCo study, the cohort may be subject to self-selection bias and is not necessarily representative of the general population; healthcare workers may have higher vaccine uptake, greater occupational exposure to SARS-CoV-2, and different health-seeking behavior than the broader population, which could limit generalizability.
Ethics oversight	This study was approved by the Institutional Review Board (Ethics Committee) of Hannover Medical School (approval no. 8973_BO_K_2020, last amendment August 2024, and approval no. 11475_4-BO_S_2024) and was conducted in accordance with the Declaration of Helsinki. The study is registered with the German Clinical Trial Registry (DRKS00021152). Written informed consent was obtained from all participants, who received no compensation for participation.

Note that full information on the approval of the study protocol must also be provided in the manuscript.

Field-specific reporting

Please select the one below that is the best fit for your research. If you are not sure, read the appropriate sections before making your selection.

[x] Life sciences [ ] Behavioural & social sciences [ ] Ecological, evolutionary & environmental sciences

For a reference copy of the document with all sections, see nature.com/documents/nr-reporting-summary-flat.pdf

Life sciences study design

All studies must disclose on these points even when the disclosure is negative.

Sample size	The sample size of n=42 was estimated to be adequate to detect a clinically meaningful difference within the group, assuming S-protein-reactive IgG levels double after vaccination (before: mean 822 RU/mL [SD 747]; after: mean 1644 RU/mL [SD 1,494]), based on a convenience sample of 24 persons from the CoCo cohort measured in August 2023.
Data exclusions	Participants who reported SARS-CoV-2 infection between vaccination and day 21 were excluded from analysis. Otherwise, unless explicitly

	indicated, all available biological replicates were included and no outliers were removed; missing values were handled by analysis-specific (pairwise) exclusion - e.g., paired tests were restricted to donors with both time points available.
Replication	All attempts at replication were successful. ELISA: >=4 independent experiments. Neutralization assays: 2 independent experiments with 4 technical replicates per dilution step. BLI affinity measurements: >=2 independent experiments (one representative shown). scRNA-seq: single experiment (n=7 donors, three time points, three sorting gates). No failed replications to report.
Randomization	Not applicable. This is an observational cohort study nested within the parent CoCo Study; participants were healthcare workers who voluntarily enrolled and received vaccination according to institutional/national recommendations, not by experimental allocation. There were no experimental groups requiring randomization.
Blinding	Investigators were blinded to individual participant data, but not to group allocation. Baseline group-level measurements, including ELISA analyses, were performed early and in a single run to minimize variability, determine appropriate sample dilutions for subsequent time points, and confirm that the seven donors selected for longitudinal B-cell analysis were representative of the entire cohort.

Reporting for specific materials, systems and methods

We require information from authors about some types of materials, experimental systems and methods used in many studies. Here, indicate whether each material, system or method listed is relevant to your study. If you are not sure if a list item applies to your research, read the appropriate section before selecting a response.

Materials & experimental systems

n/a	Involved in the study	
☐	☒	Antibodies
☐	☒	Eukaryotic cell lines
☒	☐	Palaeontology and archaeology
☒	☐	Animals and other organisms
☒	☐	Clinical data
☒	☐	Dual use research of concern
☒	☐	Plants

Methods

n/a	Involved in the study	
☒	☐	ChIP-seq
☐	☒	Flow cytometry
☒	☐	MRI-based neuroimaging

Antibodies

Antibodies used

- Alexa Fluor 488 anti-human CD16, clone 3G8, BioLegend cat# 302019, RRID: AB_492974, 1:100
- PE/Cyanine5 anti-human CD14, clone M5E2, BioLegend cat# 301864, RRID: AB_2860767, 1:100
- Brilliant Violet 570 anti-human CD20, clone 2H7, BioLegend cat# 302332, RRID: AB_2563805, 1:100
- APC/Fire810 anti-human CD38, clone HIT2, BioLegend cat# 303550, RRID: AB_2860784, 1:100
- CD3 Monoclonal Antibody (UCHT1), Alexa Fluor532, eBioscience cat# 58-0038-42, RRID: AB_11218675, 1:100
- CD27 Monoclonal Antibody (O323), Alexa Fluor700, eBioscience cat# 56-0279-42, RRID: AB_11044789, 1:100
- Pacific Blue anti-human CD19, clone SJ25C1, BioLegend cat# 363036, RRID: AB_2632787, 1:100
- PE/Cyanine7 anti-human CD19, clone SJ25C1, BioLegend cat# 363012, RRID: AB_2564203, 1:100
- Brilliant Violet 480 anti-human IgD, clone 1A6-2, BD Biosciences cat# 566138, RRID: AB_2739536, 1:100
- Zombie NIR viability dye, BioLegend cat# 423106, 1:1000
- Goat anti-human IgM (mu-chain) HRP secondary 1:2000, Thermo Fisher cat# A18841, RRID: AB_2535606
- Goat anti-human IgG (gamma-chain) HRP secondary 1:2000, Thermo Fisher cat# 62-8420, RRID: AB_2533962
- Wu01 tetramer-PE 1:100 and JN.1 tetramer-APC 1:400 ("This study" - see Validation note below)

Validation

Commercial antibody validation is documented via the RRIDs listed above (cross-referenceable at the Antibody Registry / manufacturer websites, per catalogue number). The Wu01 and JN.1 tetramers were generated in-house and have no commercial RRID. Their specificity was validated using cryopreserved pre-pandemic PBMCs from a donor subset, which defined background staining and fixed positivity thresholds for the Wu01-only, JN.1-only, and double-positive gates; background was minimal. These thresholds were held constant across all samples (Fig. 2B, Fig. S2).

Eukaryotic cell lines

Policy information about [cell lines and Sex and Gender in Research]

Cell line source(s)	HEK-293T (human, female, kidney; DSMZ, cat# ACC-635, RRID:CVCL_0063). Vero (African green monkey, female, kidney; ATCC, cat# CRL-1586, RRID:CVCL_0574; kindly provided by Andrea Maisner).
Authentication	"Cell lines were authenticated by STR analysis, partial sequencing of the cytochrome c oxidase gene and microscopic examination."
Mycoplasma contamination	"Cell lines were regularly tested for the absence of contamination by mycoplasma using an in-house PCR assay." (negative/no contamination detected)

Commonly misidentified lines (See ICLAC register)	N/A.

Plants

Seed stocks	*Report on the source of all seed stocks or other plant material used. If applicable, state the seed stock centre and catalogue number. If plant specimens were collected from the field, describe the collection location, date and sampling procedures.*
Novel plant genotypes	*Describe the methods by which all novel plant genotypes were produced. This includes those generated by transgenic approaches, gene editing, chemical/radiation-based mutagenesis and hybridization. For transgenic lines, describe the transformation method, the number of independent lines analyzed and the generation upon which experiments were performed. For gene-edited lines, describe the editor used, the endogenous sequence targeted for editing, the targeting guide RNA sequence (if applicable) and how the editor was applied.*
Authentication	*Describe any authentication procedures for each seed stock used or novel genotype generated. Describe any experiments used to assess the effect of a mutation and, where applicable, how potential secondary effects (e.g. second site T-DNA insertions, mosiacism, off-target gene editing) were examined.*

Flow Cytometry

Plots

Confirm that:

- [x] The axis labels state the marker and fluorochrome used (e.g. CD4-FITC).
- [x] The axis scales are clearly visible. Include numbers along axes only for bottom left plot of group (a 'group' is an analysis of identical markers).
- [x] All plots are contour plots with outliers or pseudocolor plots.
- [x] A numerical value for number of cells or percentage (with statistics) is provided.

Methodology

Sample preparation	Cells were labelled with a flow cytometry antibody panel (Table S2) and analyzed on a Cytek Northern Lights cytometer. Doublets were excluded by FSC-A versus FSC-H gating, and live cells were identified using a viability dye (Zombie NIR). Myeloid cells were excluded by gating out CD14+ and CD16+ events.
Instrument	Cytek Northern Lights.
Software	SpectroFlo and/or FCS Express.
Cell population abundance	Antigen-specific memory B cells were quantified both as event counts (Fig. 2C) and as a percentage of total memory B cells (Fig. S1D) at day 0, 13, and 21 post-vaccination.Wu01-only memory B cells: median count 57.5 -> 192.0 -> 143.5 (n=42, 35, 34); median percentage 0.240% [IQR 0.1375-0.4850%] -> 0.720% [IQR 0.5200-1.170%] -> 0.700% [IQR 0.4275-1.148%].Wu01-JN.1 cross-reactive memory B cells: median count 24.5 -> 88.0 -> 66.0; median percentage 0.115% [IQR 0.0500-0.1525%] -> 0.340% [IQR 0.1800-0.5300%] -> 0.325% [IQR 0.2275-0.4575%].JN.1-only memory B cells: median count 11.5 -> 19.0 -> 41.0; median percentage 0.035% [IQR 0.0300-0.0425%] -> 0.070% [IQR 0.0500-0.0900%] -> 0.140% [IQR 0.0800-0.2125%].
Gating strategy	Full gating hierarchy - doublet exclusion (FSC-A/FSC-H), viability gate, CD14+/CD16+ myeloid exclusion, CD19+ B cell gate, then Wu01/JN.1 tetramer double-positive gating - is described in Methods and shown in Fig. S2.

- [x] Tick this box to confirm that a figure exemplifying the gating strategy is provided in the Supplementary Information.
