# Supplementary material for: JN.1-adapted vaccination is associated with readjustment of ancestral memory B cells toward neutralization within the JN.1 antigenic space
Source: Nat Commun. 2026 Jul 24;17:7266. doi: 10.1038/s41467-026-76035-z (PMC13400639; doi:10.1038/s41467-026-76035-z)
Supplement: Supplementary file 7 — Transparent Peer Review file [file 41467_2026_76035_MOESM7_ESM.pdf]

nature portfolio

Peer Review File

JN.1-adapted vaccination is associated with readjustment of ancestral memory B cells toward neutralization within the JN.1 antigenic space

Corresponding Author: Professor Georg Behrens

This file contains all reviewer reports in order by version, followed by all author rebuttals in order by version.

Version 0:

Reviewer comments:

Reviewer #1

(Remarks to the Author)
The manuscript titled “Somatic hypermutation after JN.1-adapted vaccination repurposes ancestral memory B cells for future virus variant neutralization” presents crucial insights into the role of somatic hypermutation (SHM) in recalibrating memory B cells (MBCs) to improve neutralization against emerging SARS-CoV-2 variants following vaccination with a JN.1-adapted mRNA vaccine. The manuscript is well-structured and methodologically robust, providing interesting implications for vaccine development in the face of rapidly evolving pathogens like SARS-CoV-2. Below, I provide major and minor critiques to improve the quality, clarity, and accessibility of the work.

Major Critiques
1. Clarity on "Gate Shifter" Mechanism: While the concept of "gate shifters" is intriguing, the mechanisms underlying their SHM-driven specificity changes require more explicit elaboration. The manuscript provides strong evidence supporting this phenomenon and lists current limitations, but it does not delve sufficiently into whether vaccination, previous exposures, or stochastic SHM primarily induce the observed changes. Greater emphasis on experimental findings or context addressing anticipatory maturation or vaccination-induced SHM would strengthen the discussion.
2. Donor Heterogeneity and Potential Confounders: The cohort’s heterogeneity introduces potential confounding factors (prior infections, vaccination history). While this variability is acknowledged as a limitation, the manuscript could benefit from an in-depth discussion regarding its potential impact on the reproducibility of findings and implications for broader generalizability.
3. Visualization of SHM Effects: Figures illustrating "gate shifting profiles" and SHM contributions to antigen-binding affinity (e.g., Figure 3) could be further refined. For example, highlight significant differences and functional outcomes of SHM.
4. Role of Antigenic Breadth vs Neutralization Potency: The manuscript discusses binding breadth versus neutralization potency (e.g., Figure 1H). While it is noted that loss of binding does not imply loss of neutralization, this distinction could be expanded further, especially regarding the implications for vaccine design and evaluations of antibody efficacy.

Minor Critiques
1. Terminology Standardization: Some terminology (e.g., "gate shifters," "type-specific," and "exclusively JN.1-reactive") could benefit from standardization throughout the manuscript to maintain consistency and avoid ambiguity.
2. Elaboration on Neutralizing mAbs: The manuscript highlights interesting neutralization findings, particularly for mAbs like CT5-2. However, the implications for monoclonal antibody (mAb) development and clinical applications are touched upon briefly but warrant further elaboration.
3. Additional Statistical Insights: While the manuscript includes robust statistical analyses, additional information on key metrics (e.g., effect sizes, statistical power of observed changes) in some sections could bolster quantitative understanding. Figure 1K (neutralization efficiency) is shown for the post-vaccination time point, but a comparison to the pre-vaccination time point would be critical for a complete assessment. Show an example for a relative binding units (RU) standard curve in Figure S1.

Conclusion
The manuscript delivers significant contributions to our understanding of memory B cell adaptation and vaccine-induced immunity in response to SARS-CoV-2 evolution. By addressing the critiques outlined above, the authors could further strengthen the impact and clarity of their findings. Specifically, providing greater mechanistic depth on SHM-driven adaptability, improving visual representations, and integrating a more comprehensive discussion of implications will enhance this already impressive study.
I suggest major revisions before reconsideration for publication.

Reviewer #2

(Remarks to the Author)
Overall Summary:
This manuscript by Stankov et al. presents an investigation into the B-cell responses following JN.1 mRNA vaccination. The study demonstrates that vaccination can repurpose pre-existing memory B cells (MBCs) through somatic hypermutation (SHM), leading to enhanced neutralization of JN.1 and subsequent variants. The identification of the "gate shifter" is a novel finding. The manuscript could be improved by incorporating more rigorous quantitative analyses, clearer justification of the statistical methods, and more cautious language when discussing causal inferences.

Major Points for Revision:
1. The concept of "neutralization efficiency " (NT50/OD450) in Figure 1K is potentially misleading. The OD450 from a single serum dilution is not a quantitative measure of antibody concentration or titer. To make this metric more interpretable, it is recommended to use quantitative binding data, such as the relative units (RU) derived from standard curves (mentioned in Figure S1) or endpoint titer from titration curves. Similarly, the data presentation in Figures 1E-G, 3D-F, and 4C should be improved.
2. Figure 1J indicates high pre-existing neutralization titers (NT50~10^3) at Day 0, consistent with the cohort's high rate of prior infection (87.8%). The authors should discuss how this robust baseline immunity might influence the observed fold-increases post-vaccination. A subgroup analysis (e.g., comparing individuals with recent vs. remote infections) or a discussion on whether the fold-change might be underestimated due to high starting titers would provide a more nuanced interpretation of the serum antibody boosting data.
3. As the "gate shifter" concept is fundamental to the manuscript's conclusions, quantitative evidence would strengthen it. Quantitative assessments are needed to demonstrate its prevalence in Figure 2J and 3B. The authors may report the proportion of expanded clonotypes that exhibit gate-shifting behavior and the relative contribution of these clonotypes to the total antigen-reactive MBC pool post-vaccination. This would solidify the claim that this is a representative response pattern.
4. The functional data for the recombinant mAbs in Figures 3D-F and 4C lack statistical analysis. When comparing gate-shifters to their non-shifter clonotype members (Figure 3G), appropriate statistical tests (e.g., paired t-test or Mann-Whitney U test) should be applied to confirm the significance of the observed differences in Wu01 binding and JN.1 neutralization. The figure legends should explicitly state the statistical tests used, the number of experimental replicates (technical/biological), and exact p-values.
5. The nature of CT3 (non-Spike binding) and CT4 (non-RBD binding) mAbs, which were isolated via antigen baiting, requires discussion. Are these genuine, low-affinity MBCs, or do they target non-RBD epitopes (e.g., NTD)? A brief analysis of their heavy-chain sequences and a discussion of their potential targets or classification as background noise would improve the transparency of the screening process and the interpretation of the MBC repertoire.
6. While the "Limitations" section appropriately notes the potential influence of prior infections, the language in the abstract, results, and discussion often implies a direct causal effect of the JN.1 vaccine on inducing gate-shifting (e.g., "repurposes" in the title). The language should be moderated throughout to reflect the observational nature of the study, using phrases like "associated with JN.1-adapted vaccination" rather than definitive causal statements.
7. The discussion is strong on mechanistic insights but could be enhanced by directly addressing the implications for vaccine strategy. For example, how do the findings on "forward-recognition" and SHM-driven "pre-adaptation" inform the timing of future vaccine updates, the design of broad-spectrum vaccines, or booster strategies for high-risk populations?
8. Relevant background information of the study subjects should be included in the abstract to enable readers to quickly assess the reliability of the study design. To more concisely highlight the central mechanistic discovery in the abstract, the authors could state: "Single-cell RNA-sequencing revealed that this refinement was driven by somatic hypermutation in pre-existing cross-reactive memory B cells, causing a shift in their specificity towards the novel variant ('gate-shifting'), rather than the expansion of naive-derived, JN.1-specific clones."
9. Since T-cell responses play a crucial role in defense against viral infection, it is advisable to include corresponding evidence or discussion regarding this aspect.
10. Improve the annotation of the phylogenetic trees to indicate what the branch lengths represent in Figure 3B. Corresponding methodological descriptions should be included.
11. Several minor issues should be corrected:
• line 111, superscript "8" in the caption should be verified.
• “The cohort received a median of 4.5 previous COVID-19 vaccinations, and 87.8% of the included individuals reported at least one prior SARS-CoV-2 infection (Table S2).” Table S2 should be Table S1.
• vacccination in Table S1should be corrected to vaccination.

Reviewer #3

(Remarks to the Author)
The manuscript by Stankov et al., entitled "Somatic hypermutation after JN.1-adapted vaccination repurposes ancestral memory B cells for future virus variant neutralization" characterized the antibody specificity of circulating neutralizing antibodies and memory B cells followed by JN.1-adapted mRNA vaccine. The authors aimed to dissect how immunological imprinting by ancestral mRNA vaccines is overridden by the JN.1 booster. Serological analysis confirmed the robust induction of antibody responses to JN.1 and its descendent strains, such as KP.2, KP.3, KP.3.1.1, and XEC, in the analyzed cohort. Longitudinal tracking of memory B cell specificity demonstrated the gain of JN.-1-reactivity over time. In-depth analysis by scRNA-seq failed to show the phenotypic changes of memory B cells, but revealed the several BCR clonotypes showing the specificity shift from Wuhan to JN.1 by introducing SHMs. As cited, SHM-driven modification of memory B cell specificity is shown to override immunological imprinting by ancestral mRNA vaccines after repeated Omicron BA.5 exposure, but not by more recent JN.1 vaccine. The detailed analysis of JN.1-boosted memory B cell responses at monoclonal levels remains limited in my view, making the topics interesting and timely for the readership of this journal. The followings are the list of my suggestions.

Comments:
1) The phrase “future virus variant neutralization” in the title seems overreaching to me based on the data presented in the current manuscript. Whereas JN.1-adapted vaccination shifted the antibody and memory B cell specificity to JN.1 and its descendent variants, the data do not demonstrate the reactivity to unrelated variants which may emerge in the future. I suggest the revision of the title.

2) There is a mismatch in the virus strains used in Figure 1. The authors used KP.2, KP.3, and XEC variants for detecting RBD-binding antibody titers (Figure 1E-G), and KP.3, KP.3.1.1, and XEC (not KP.2) for NT titers (Figure 1J). However, the ratios (NT/binding titers) in Figure 1K were somehow calculated using KP.2, KP.3, and XEC. I do not understand how this is possible.

3) KP.3.1.1 appears to be less immune evasive than KP.3, XEC, and its parent JN.1 strain based on NT titers in Figure 1J. Please explain why.

4) BCR clonotype analysis in Figure 3 is one of core data in this manuscript for supporting the presence of “gate shifter” at BCR gene levels. However, there are not sufficient information in sequence data of the analyzed BCR in the current format, making it hard to interpretate the results as claimed by the authors. The authors need to present the BCR sequence data of all clones belonging to CT1, CT2, CT3, CT4, and CT5 (Figure 3B) and clarify the positions of SHM, the names of VDJ/VJ gene template, and CDR3 sequences. These BCR sequencing data need to be deposited in public database with the specific clone names as well.

5) Related to comment 4, I cannot understand which clones are CT2-1/-2/-3/-4/-5/-6/-7 in clonal tree of Figure 3B. The same is true for CT5 clones. I would like to see the aligned sequence data with information on SHM, V gene templates, and CDR3 sequences.

6) CT3 clones failed to bind spike, S1 and RBD. I assume that they are not neutralizers. CT4 clones bound to spike and S1, but not RBD, and they were non-neutralizers (Figure 3). Because the authors cannot exclude the possibility that the CT3 and CT4 are non-specific clones which are contaminated in FACS-sorting B cells at certain levels, I strongly suggest that these clones should be excluded from the analysis. I also suggest to reconsider the following description in line 178 “For the RBD, CT4 did not exhibit any binding, presumably because it was unable to bind to RBD alone in the absence of other non-RBD domains”, since CT4-3 clone was isolated as JN.1 RBD binder in flow cytometry. Without showing the RBD binding affinity above the detectable levels using more sensitive methods, such as biolayer interferometry, the authors cannot conclude that CT3 and CT4 clones are indeed vaccine-induced clones (they are more likely non-specific clones).

7) The authors did not prepare the germline-reverted mAbs from CT2 and CT5. However, an additional importance piece of data supporting the gate shifter phenomenon is to show Wuhan-specific binding and/or NT activity in the ancestral antibody clones encoded by germline VH/VL genes.

Reviewer #4

(Remarks to the Author)
The manuscript by Stankov et. al. investigated how JN.1-adapted COVID-19 vaccination modulates antibody and memory B cell immunity in response to ongoing SARS-CoV-2 antigenic drift. The authors showed that the adapted vaccine boosts IgG binding and neutralization against JN.1 and related variants. Longitudinal analyses seemed to reveal a progressive shift in memory B cell specificity, with an increase in exclusively JN.1-reactive cells and a decline in Wu01-cross-reactive populations. The authors also used scRNAseq data to support their observations.

The experiments appear carefully performed and the results were mostly clear. Understanding how variant-adapted vaccines repurpose pre-existing memory B cells has significant implications for developing next-generation COVID-19 vaccines capable of countering ongoing SARS-CoV-2 evolution. However, there are numerous points that impact the data analysis and presentation.

Specific points
1. The presented data is only for IgG responses. Were IgM responses to JN.1 and other recent variants assessed? If IgM

responses are weak, that would further support the interpretation that the B cells reacting exclusively to JN.1 originate from pre-existing MBC.

2. In Figure 1E, the authors compare raw OD450 values across ELISAs with different antigens (and/or plates). This is problematic, as OD450 is assay-, plate-, and antigen-dependent, and small differences in coating, incubation, or substrate development can substantially affect readings. It is unclear what methods were used to account for these confounders, such as standard curves or duplicate wells or if there was a normalization procedure. The data in Figure S1 showing calculated relative binding units based on standard curves appear more reasonable. Although the results show a trend, it is not as pronounced as presented in Figures 1E–G. This discrepancy suggests selective data presentation that does not seem justified. Full curve ELISA results that give ED50 or endpoint titers, with methods to normalize / standardize across plates would be appropriate; alternatively, comparisons can be restricted to the same assay/plate. Consider moving Figure S1 data to the main figures and relocating Figure 1E–G to the supplement. Do all figures focus exclusively on comparisons between JN.1 and other variants? For example, in Figure 1G, the differences between Wu01 and the other recent variants also appear substantial. The authors should clarify the statistical methods used for each comparison, ideally in the figure legends.

3. In Figure 1K, this metric seems contrived, as NT50 and ELISA OD450 represent distinct and non-comparable units. The biological interpretation of the ratio is of unclear value, and the index does not appear to be standardized across assays. And as mentioned above, presenting the data solely as raw OD450 values is problematic, let alone deriving a ratio based on those measurements.

4. In figure S2, there are two concerns regarding the gating strategy.:
A) In the first gating step, it is unclear why not all cells are included. After B-cell proliferation, cells become larger and more granular (FSC/SSC increase), so excluding cells at this stage may omit relevant populations.
B) It is unclear why antibody-secreting cells (ASCs) are excluded prior to gating B cells. If ASCs refer to CD27+CD38high cells, the commonly used term would be plasmablasts. The gating shown for CD27 and CD38 plot appears inappropriate, excluding CD27+CD38high cells before gating B cells. Plasmablasts express surface BCR capable of recognizing the baits and represent an important component of antigen-specific B cells that should be included in the analysis. The current gating strategy raises concerns about the reliability of the results. I recommend revisiting the gating strategy to ensure that all relevant B cell populations, including plasmablasts, are properly captured.

5. Page 6, lines 129-132: Overall, Wu01-specific B cells still constitute the majority, while single JN.1-reactive and cross-reactive populations remain much smaller. This does not appear to represent a qualitative shift in preferred antigen specificity. I also have a concern regarding the tetramer staining: have the authors tested negative control samples (e.g., PBMCs from healthy donors prior to the COVID-19 pandemic) to assess background staining? Non-specific binding is an important consideration, especially since cross-reactivity between SARS-CoV-2 and common human coronaviruses has been reported.

6. Lines 132-134: This may represent an overstatement of the data. It is also possible that some of the observed responses reflect de novo induction of new B-cell clones. Notably, the percentage of Wu01 single-reactive cells on day 12 shows a declining trend, and the absolute numbers of these cells are much higher than the other two populations. The frequencies of JN.1-reactive cells, whether single-antigen or cross-reactive, are not constant over time. Moreover, the lack of absolute B-cell counts limits the ability to draw definitive conclusions about shifts in population size or antigen specificity.

7. Lines 154-155: Have the authors performed a pseudotime/cell trajectory analysis for these B-cell populations? Such an analysis could provide insights into the developmental relationships between Wu01-specific, JN.1-specific, and cross-reactive B cells, and clarify whether observed shifts in frequency reflect maturation, clonal expansion, or recruitment of new clones over time.

8. Lines 159-163: If both processes, maturation of pre-existing clones and de novo induction of new B cell clones, occur simultaneously and at similar levels, could this also produce the observed patterns?

9. In Figure 3D, as noted earlier, OD450 values can be influenced by many factors. I recommend presenting these data using EC50 or endpoint titers. And just to clarify, does ‘WT’ in Figures 3 and 4 refer to Wu01? The labeling should be kept consistent throughout the figures.

10. In Figure 3, it is possible that the JN.1-reactive clones captured in this gate have higher avidity, which could make them more likely to be recognized by the bait. This is an interesting possibility, and measuring BCR avidity could provide valuable insight to support your observations.

11. Figure 3G, how was the cutoff for this gate shifting zone determined? These cutoffs appear to be arbitrary to suit the story. If it is a real metric, it should be consistent across different clones, or at least have a rationale for the shaded areas.

Minor comments:
1. In Table S2, the authors should specify the types of vaccines received by the participants to provide clarity and context for the reported responses.

2. The authors should include a dedicated Statistical Analysis section in the Methods to describe all analyses performed. Additionally, the statistical tests used for each figure should be clearly indicated in the corresponding figure legends.

3. Page 5, lines 96-97: “Consequently, a pronounced…the COVID-19 pandemic was observed.” was repeated, which also mentioned in lines 91-93.

4. Line 109: The statement ‘all tested variants increased’ is inaccurate, as XBB.1.5 did not show a statistically significant increase. Additionally, in Figure 1J, it is unclear whether the titers and fold changes shown above the figure represent mean/median values; the authors should clarify this and ensure precise reporting.

5. In Figure 1J, I noticed that the fold changes for XEC in the cited study do not match what is reported here. I’m just curious why this is the case, especially since it appears to be from the same CoCo cohort.

6. The results sections lack concise summaries, and the logical transitions between experiments are not fully supported. For example, at the end of the first results section (lines 115–120), the text is wordy and could be more clearly and succinctly presented.

7. Lines 125-126: This may leave readers uncertain whether the first two populations are exclusive to a single antigen or could include cross-reactive cells. I recommend explicitly stating that the first two populations are reactive only to a single antigen, for example: We classified B cells as Wu01 S-specific or JN.1 RBD-specific, representing cells reactive exclusively to a single antigen, and also identified cross-reactive B cells that were labeled by both antigens.

8. Page 6, lines 126-128: The current wording implies that B-cell frequency inevitably increases after vaccination. This seems overly absolute.

9. Line 148: It should be day 13 instead of 14. The same in Figure S4B.

Reviewer #5

(Remarks to the Author)
I co-reviewed this manuscript with one of the reviewers who provided the listed reports. This is part of the Nature Communications initiative to facilitate training in peer review and to provide appropriate recognition for Early Career Researchers who co-review manuscripts.

Version 1:

Reviewer comments:

Reviewer #1

(Remarks to the Author)
The authors have satisfactorily addressed all of my concerns. I recommend that the manuscript be accepted for publication.

Reviewer #2

(Remarks to the Author)
The revised manuscript is significantly improved and addresses most prior concerns raised in the previous round. The authors also appropriately softened some of the causal language and now acknowledge several limitations more explicitly. However, the study still has substantial design and interpretation issues that limit the strength and generalizability of its conclusions.

Major Comments
1. Cohort heterogeneity is a fundamental design limitation that directly affects the main conclusion. The study cohort is highly pre-exposed, with a median of 4.5 prior COVID-19 vaccinations and 87.8% of participants reporting at least one prior SARS-CoV-2 infection. In such a cohort, the observed B-cell repertoire features, including the proposed gate-shifting phenomenon, are difficult to attribute specifically to the current JN.1-adapted booster rather than to prior infection, prior variant-adapted vaccination, or cumulative antigenic exposure. The manuscript reports inverse correlations between baseline binding or neutralization titers and post-vaccination fold changes, which the authors interpret as ceiling-limited boosting in this highly pre-exposed cohort. This is a reasonable interpretation, but it also means that pooled fold-change analyses are difficult to interpret biologically unless the cohort is stratified by baseline immunity, recent infection, or last exposure type. Without such analyses, it remains unclear whether observed boosting patterns reflect booster-specific immune refocusing or simply differences in starting immune state. The authors acknowledge this limitation, but it should be treated as a central design constraint rather than a secondary caveat. The authors are recommend to conduct exploratory subgroup analyses based on detailed infection and vaccination history (which should also be provided in detail) to compare immune responses across participants. Furthermore, causal language should be moderated throughout the manuscript.
2. The revised title is more appropriately cautious, which is a welcome change. However, portions of the abstract, results, and discussion still risk implying that the JN.1 booster itself drove the gate-shifting phenomenon. Given the observational design and the authors’ own acknowledgment that they cannot definitively distinguish booster-induced effects from prior exposure-driven maturation, the language throughout should remain consistently associative.
3. The scRNA-seq analysis was performed in only 7 participants selected as representative based on serum antibody titers

and neutralization capacity. However, representativeness in serology does not necessarily imply representativeness in exposure history or clonal immune architecture. The manuscript would be strengthened by a clearer justification of the sampling strategy and a more explicit discussion of how this selective subsampling may bias conclusions about the prevalence and biological relevance of gate-shifting clonotypes.
4. The manuscript notes that only a minority of cells from expanded clonotypes exhibited gate-shifting behavior. This raises an important interpretive question: is gate shifting a representative population-level mechanism, or a biologically interesting but relatively uncommon phenomenon? The authors should provide clearer quantitative reporting, including the proportion of expanded clonotypes showing gate shifting, their contribution to the total antigen-reactive MBC pool, and the consistency of this phenomenon across donors.
5. Broader conclusions about vaccine strategy are somewhat ahead of the data. The discussion extends toward implications for iterative vaccine updating, breadth, and correlates of protection. While this is reasonable to discuss, the study does not assess T-cell responses, which are also relevant to SARS-CoV-2 protection. In the absence of such data, claims about broader vaccine strategy or immune correlates should be moderated further.
6. The authors are advised to more carefully verify the data in Figure 1 and the manner of data presentation.

Reviewer #3

(Remarks to the Author)
The authors revised the manuscript in sufficient way. No further comments.

Reviewer #4

(Remarks to the Author)
The authors have revised the manuscript in accordance with our comments. In particular, they now use ED50 for all ELISA data instead of relying on a single point OD450. They have done BLI and trajectory analysis as recommended, and have also moderated their interpretations and avoided over-interpreting the data.

The manuscript has been substantially improved.

One remaining minor issue is that assessing IgM responses using pooled sera is understandable but not ideal, as it may obscure the true variability and responses of individual samples. However, this is a minor point and does not preclude inclusion of those data.

Additionally, in Figure S2, the gating strategy contains incorrect labels on the top, which should be corrected.

Reviewer #5

(Remarks to the Author)
I co-reviewed this manuscript with one of the reviewers who provided the listed reports. This is part of the Nature Communications initiative to facilitate training in peer review and to provide appropriate recognition for Early Career Researchers who co-review manuscripts.

Version 2:

Reviewer comments:

Reviewer #2

(Remarks to the Author)
The authors have addressed all the raised comments accordingly.No further comments.

Reviewer #4

(Remarks to the Author)
I appreciate the additional revisions.

Reviewer #5

(Remarks to the Author)
I co-reviewed this manuscript with one of the reviewers who provided the listed reports. This is part of the Nature Communications initiative to facilitate training in peer review and to provide appropriate recognition for Early Career Researchers who co-review manuscripts.

Open Access This Peer Review File is licensed under a Creative Commons Attribution 4.0 International License, which permits use, sharing, adaptation, distribution and reproduction in any medium or format, as long as you give appropriate credit to the original author(s) and the source, provide a link to the Creative Commons license, and indicate if changes were made.
In cases where reviewers are anonymous, credit should be given to 'Anonymous Referee' and the source.
The images or other third party material in this Peer Review File are included in the article's Creative Commons license, unless indicated otherwise in a credit line to the material. If material is not included in the article's Creative Commons license and your intended use is not permitted by statutory regulation or exceeds the permitted use, you will need to obtain permission directly from the copyright holder.
To view a copy of this license, visit https://creativecommons.org/licenses/by/4.0/

Point-by-Point Reply

JN.1-adapted vaccination is associated with readjustment of ancestral memory B cells toward neutralization within the JN.1 antigenic space

Reviewer #1

The manuscript titled "Somatic hypermutation after JN.1-adapted vaccination repurposes ancestral memory B cells for future virus variant neutralization" presents crucial insights into the role of somatic hypermutation (SHM) in recalibrating memory B cells (MBCs) to improve neutralization against emerging SARS-CoV-2 variants following vaccination with a JN.1-adapted mRNA vaccine. The manuscript is well-structured and methodologically robust, providing interesting implications for vaccine development in the face of rapidly evolving pathogens like SARS-CoV-2. Below, I provide major and minor critiques to improve the quality, clarity, and accessibility of the work.

We thank Reviewer #1 for the encouraging summary of our work and for the valuable suggestions.

Major Critiques

1. Clarity on "Gate Shifter" Mechanism: While the concept of "gate shifters" is intriguing, the mechanisms underlying their SHM-driven specificity changes require more explicit elaboration. The manuscript provides strong evidence supporting this phenomenon and lists current limitations, but it does not delve sufficiently into whether vaccination, previous exposures, or stochastic SHM primarily induce the observed changes. Greater emphasis on experimental findings or context addressing anticipatory maturation or vaccination-induced SHM would strengthen the discussion.

We thank Reviewer #1 for highlighting the incomplete discussion of the interplay between anticipatory maturation and vaccination-induced SHM. We have revised and expanded this section to further elaborate on the potential roles of virus variant-adapted vaccination and breakthrough infections. In addition, we have placed our findings into a broader framework in which stochastic/untargeted SHM, followed by selection upon variant re-exposure, likely drives the continued evolution of immunodominant antibodies in the context of SARS-CoV-2. Along with other sections (see below), the discussion has been refined accordingly (lines 333–341).

2. Donor Heterogeneity and Potential Confounders: The cohort's heterogeneity introduces potential confounding factors (prior infections, vaccination history). While this variability is acknowledged as a limitation, the manuscript could benefit from an in-depth discussion regarding its potential impact on the reproducibility of findings and implications for broader generalizability.

We agree that cohort heterogeneity arising from prior infections and multiple vaccinations, including virus variant-adapted vaccine formulations, represents a potential confounder. We have therefore expanded the limitations section to explicitly address how heterogeneous exposure histories may have influenced baseline immunity and subsequent booster responses, and how these factors limit attribution of the observed maturation effects to the JN.1-adapted vaccine alone (Discussion/Limitations, lines 368–380). At the same time, we noted that this exposure pattern reflects the current real-world context in which updated boosters are deployed, supporting the practical relevance of our observations. As the cohort size does not permit adequately powered stratified analyses, we did not perform subgroup stratification.
To clarify how this issue could be addressed experimentally, we have added a forward-looking statement noting that prospective studies in antigen-naïve individuals and/or controlled prime–boost schedules (with defined infection status and variant exposures) will be required to more

clearly disentangle the relative contributions of vaccination versus infection to gate-shifting and SHM-driven repertoire refinement.

3. Visualization of SHM Effects: Figures illustrating "gate shifting profiles" and SHM contributions to antigen-binding affinity (e.g., Figure 3) could be further refined. For example, highlight significant differences and functional outcomes of SHM.

We thank Reviewer #1 for suggesting that we should further investigate the impact of SHM on the antigen-binding affinity. In response, we expanded the analyses presented in Figure 3 by including ELISA data for CT2-germline and CT5-germline variants. All ELISA titrations were repeated in 2 additional independent experiments, each including 2 technical replicates (e.g., Fig. 3E). However, the differences observed in ELISA were not statistically significant. For clarity and more appropriate visualization of these results, we redesigned Fig. 3G and now present the data as heatmaps rather than dot plots. To further substantiate our findings, we additionally assessed the functional consequences of SHM using bio-layer interferometry measurements (see Fig. 4D, Fig. S5, and Supplementary Data 1). These new experiments demonstrate that binding affinity to JN.1 strongly depends on the presence of the specific SHM identified in our study (Fig. 4D). The results of these new analyses are described in the revised Results section (lines 227-284).

4. Role of Antigenic Breadth vs Neutralization Potency: The manuscript discusses binding breadth versus neutralization potency (e.g., Figure 1H). While it is noted that loss of binding does not imply loss of neutralization, this distinction could be expanded further, especially regarding the implications for vaccine design and evaluations of antibody efficacy.

We thank Reviewer #1 for following up on the notion that loss of binding does not necessarily imply loss of neutralization. To further clarify this distinction and for better visualization, we substantially revised Fig. 1 (see Fig. 1E+F vs. Fig. 1J in the revised manuscript). In addition, monoclonal antibodies such as CT5-2 display a similar pattern of reduced binding to JN.1 in ELISA and bio-layer interferometry (BLI), while still fully neutralizing JN.1. Notably, the gate-shifting mAbs CT2-7 and CT5-2 even showed enhanced neutralization of JN.1. We have highlighted this aspect in the revised title (“readjustment … towards neutralization”) and added a new paragraph in the Discussion (lines 391-397).

Minor Critiques

1. Terminology Standardization: Some terminology (e.g., "gate shifters," "type-specific," and "exclusively JN.1-reactive") could benefit from standardization throughout the manuscript to maintain consistency and avoid ambiguity.

We implemented terminology standardization throughout the manuscript.

2. Elaboration on Neutralizing mAbs: The manuscript highlights interesting neutralization findings, particularly for mAbs like CT5-2. However, the implications for monoclonal antibody (mAb) development and clinical applications are touched upon briefly but warrant further elaboration.

We thank Reviewer #1 for highlighting the importance of mAb development for clinical applications. Tixagevimab is a mAb that was used in clinics and had to be taken off the market due to its inability to neutralize virus variants. Importantly, the SHM we detected in CT5-2 informs on how to “revive” Tixagevimab to neutralize all virus variants that we tested. We think our concept of identifying “gate shifters” and using their SHM information to adapt mAbs to immune escape variants can have important clinical implications for the design of future anti-infective antibodies. This aspect is highlighted in the revised Fig. 4, and in the revised Discussion (lines 351-366).

3. Additional Statistical Insights: While the manuscript includes robust statistical analyses, additional information on key metrics (e.g., effect sizes, statistical power of observed changes) in some sections could bolster quantitative understanding. Figure 1K (neutralization efficiency) is shown for the post-vaccination time point, but a comparison to the pre-vaccination time point would be critical for a complete assessment. Show an example for a relative binding units (RU) standard curve in Figure S1.

We thank Reviewer #1 for highlighting the need for additional statistical assessment. In response, we have substantially revised and strengthened our data and improved the statistical descriptions throughout the manuscript. Most importantly, we now present ED50 titrations of all serum samples instead of the OD450 measurements shown in the original submission. Consequently, the RU standard curves have been removed from the supplementary information, as they are no longer required. Additionally, Fig. 1K has been removed in response to the comment 1 of Reviewer #2 (see below). For comparison, pre- and post-vaccination NT50 values are now shown side-by-side in Fig. 1J.

Conclusion
The manuscript delivers significant contributions to our understanding of memory B cell adaptation and vaccine-induced immunity in response to SARS-CoV-2 evolution. By addressing the critiques outlined above, the authors could further strengthen the impact and clarity of their findings. Specifically, providing greater mechanistic depth on SHM-driven adaptability, improving visual representations, and integrating a more comprehensive discussion of implications will enhance this already impressive study. I suggest major revisions before reconsideration for publication.

We thank Reviewer #1 for the helpful criticism.

Reviewer #2

Overall Summary:

This manuscript by Stankov et al. presents an investigation into the B-cell responses following JN.1 mRNA vaccination. The study demonstrates that vaccination can repurpose pre-existing memory B cells (MBCs) through somatic hypermutation (SHM), leading to enhanced neutralization of JN.1 and subsequent variants. The identification of the "gate shifter" is a novel finding. The manuscript could be improved by incorporating more rigorous quantitative analyses, clearer justification of the statistical methods, and more cautious language when discussing causal inferences.

We thank Reviewer #2 for carefully summarizing our study and pointing out the novelty of the "gate shifting" concept.

Major Points for Revision:

1. The concept of "neutralization efficiency " (NT50/OD450) in Figure 1K is potentially misleading. The OD450 from a single serum dilution is not a quantitative measure of antibody concentration or titer. To make this metric more interpretable, it is recommended to use quantitative binding data, such as the relative units (RU) derived from standard curves (mentioned in Figure S1) or endpoint titer from titration curves. Similarly, the data presentation in Figures 1E-G, 3D-F, and 4C should be improved.

We thank Reviewer #2 for this valuable suggestion. After careful consideration of the experimental limitations, we agree that the concept proposed in the original Fig. 1K could be misleading and therefore removed it from the revised version of the manuscript. We also agree with Reviewer #2 that OD450 values derived from a single serum dilution are not sufficiently informative due to potential ELISA saturation effects. During the revision, we therefore re-measured all serum samples using full ELISA titration curves to determine ED50 values. Each sample was analyzed in two independent experiments with two technical replicates per experiment. The newly derived data (see Fig. 1E, F and G) confirm the conclusions drawn from our initial ELISA experiments. Notably, the differences between variants were even more pronounced when assessed by ED50 titers, necessitating presentation on a logarithmic scale (see Fig. 1 below). The Results section has been substantially revised to reflect these chances (lines 118-131).

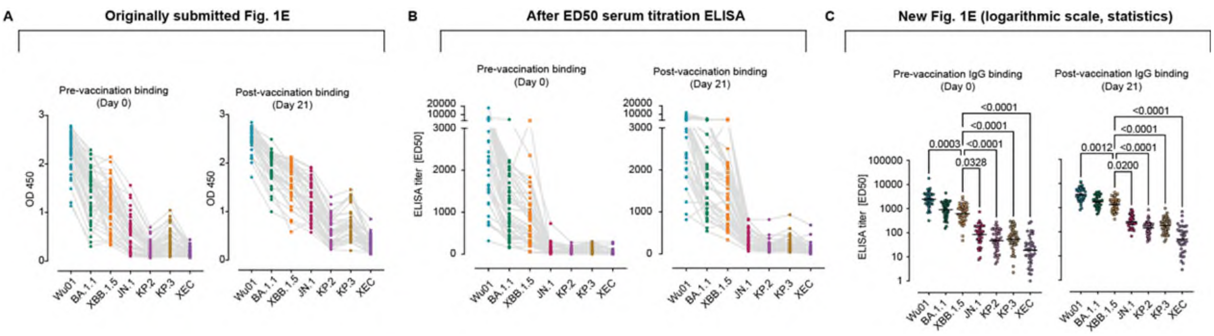

Figure 1: Changes applied to Fig. 1E of the manuscript. (A) The originally submitted figure, comparing single-dilution OD450 values pre- and post-vaccination. **(B)** After ED50 titration, the immune escape effect was too pronounced to be plotted on linear scale, because it was insufficiently resolved to allow discrimination of differences between JN.1, KP.2, KP.3 and XEC. **(C)** After logarithmic plotting and statistical testing, we included this data representation in the revised manuscript.

2. Figure 1J indicates high pre-existing neutralization titers (NT50~10^3) at Day 0, consistent with the cohort's high rate of prior infection (87.8%). The authors should discuss how this robust baseline immunity might influence the observed fold-increases post-vaccination. A subgroup analysis (e.g., comparing individuals with recent vs. remote infections) or a discussion on whether the fold-change might be underestimated due to high starting titers would provide a more nuanced interpretation of the serum antibody boosting data.

We thank Reviewer #2 for this suggestion. We followed up on this question and found that higher baseline ED50 and NT50 titers inversely correlated with the magnitude of the booster responses regarding binding and neutralization, respectively (see Fig. S1). This pattern suggests a ceiling effect that limits the magnitude of responses after boosting, which we now describe in the revised manuscript (lines 131-135). We refrained from stratifying the cohort into subgroups, because the resulting sample sizes would have been too small to provide adequate statistical power.

3. As the "gate shifter" concept is fundamental to the manuscript's conclusions, quantitative evidence would strengthen it. Quantitative assessments are needed to demonstrate its prevalence in Figure 2J and 3B. The authors may report the proportion of expanded clonotypes that exhibit gate-shifting behavior and the relative contribution of these clonotypes to the total antigen-reactive MBC pool post-vaccination. This would solidify the claim that this is a representative response pattern.

We thank Reviewer #2 for this suggestion and included this information in the revised Figure 3 (see Fig. 3C in the revised manuscript). Importantly, peripheral blood sampling likely underestimates the actual frequency of gate-shifting MBC due to relatively small sampling depth in comparison to the total blood volume. But this is a natural limitation in all observational human vaccine studies. We describe these results and their imitations in the revised manuscript (lines 221-223).

4. The functional data for the recombinant mAbs in Figures 3D-F and 4C lack statistical analysis. When comparing gate-shifters to their non-shifter clonotype members (Figure 3G), appropriate statistical tests (e.g., paired t-test or Mann-Whitney U test) should be applied to confirm the significance of the observed differences in Wu01 binding and JN.1 neutralization. The figure legends should explicitly state the statistical tests used, the number of experimental replicates (technical/biological), and exact p-values.

We repeated the ELISA in 2 additional independent experiments, each with 2 technical replicates. The differences in mAb binding described in the original manuscript were fully confirmed, i.e., the gate-shifting mAbs exhibited the lowest EC50 values against Wu01 and also neutralized the ancestral B.1 virus less potently than JN.1. Nevertheless, pairwise statistical testing to determine whether the gate-shifting mAbs showed “significantly” reduced binding of Wu01 did not reach formal significance. This outcome is primarily attributable to (i) the moderate effect size and (ii) the large number of tests required due to the number of mAbs analyzed, which increased p-values after correction for multiple hypothesis testing.

Rather than repeating the experiment until statistical significance was achieved, we instead chose to present the averaged data as a heatmap in the revised Fig. 3G. Furthermore, we refrained from implying statistical significance in the description of these results. This visualization approach is consistent with common practice in literature[1]. The conclusions drawn from these data are supported by convergent evidence from multiple independent assays, including KD differences determined by BLI, ELISA EC50 measurements, pseudovirus neutralization assays and germline reversion studies. Together, these complementary lines of evidence support the robustness of the described findings despite the absence of formal statistical significance in this specific comparison.

5. The nature of CT3 (non-Spike binding) and CT4 (non-RBD binding) mAbs, which were isolated via antigen baiting, requires discussion. Are these genuine, low-affinity MBCs, or do they target non-RBD epitopes (e.g., NTD)? A brief analysis of their heavy-chain sequences and a discussion of their potential targets or classification as background noise would improve the transparency of the screening process and the interpretation of the MBC repertoire.

We thank Reviewer #2 for raising this point, which at first glance could suggest contamination of our baiting procedure with unspecific clones. In our revision experiments, we used bio-layer interferometry and found that the ELISA-negative clonotype CT3 is in fact a low-affinity binder. As expected for a "gate-shifting" MBC, CT3-4 showed higher affinity for the JN.1 RBD than for the Wu01 RBD (Fig. S5 and Supplementary Data 1). We speculate that even very low-affinity MBCs may be detected through avidity effects, which we discussed in the revised version of the manuscript (lines 385-391). For CT4, the ELISA binding to trimeric spike and S1 was strong, making non-specific binding very unlikely. However, we could not detect binding to RBD. This finding is addressed in the Results section of the revised manuscript (lines 230-232). Since our downstream analyses were based on RBD ELISA results, CT3 and CT4 were not further studied, as stated in the Results section.

6. While the "Limitations" section appropriately notes the potential influence of prior infections, the language in the abstract, results, and discussion often implies a direct causal effect of the JN.1 vaccine on inducing gate-shifting (e.g., "repurposes" in the title). The language should be moderated throughout to reflect the observational nature of the study, using phrases like "associated with JN.1-adapted vaccination" rather than definitive causal statements.

We agree with this criticism, and have accordingly moderated our causal language (e.g., removing "repurposes" from the title and instead added "is associated with"). At the same time, we note that all gate-shifting antibodies were identified after vaccination (as discussed in lines 368-372), which is at least suggestive of a potential contribution of the JN.1-adapted vaccination. However, future studies involving larger cohorts and greater numbers of antibodies will be required to more definitively clarify this relationship.

7.The discussion is strong on mechanistic insights but could be enhanced by directly addressing the implications for vaccine strategy. For example, how do the findings on "forward-recognition" and SHM-driven "pre-adaptation" inform the timing of future vaccine updates, the design of broad-spectrum vaccines, or booster strategies for high-risk populations?

We have strengthened the Discussion (lines 330-344) to more thoroughly address the aspect of forward recognition and SHM-driven pre-adaptation. In addition, we have included a paragraph outlining the potential implications for mAb design (line 351-366). Concerning the timing of vaccine updates and booster strategies for high-risk populations, our study did not evaluate different vaccination schedules and did not focus on individuals from high-risk groups (e.g., elderly individuals or those with comorbidities). Nevertheless, our findings demonstrate that immune imprinting can be overcome through repurposing of pre-existing MBC. We hope that these insights will inform future considerations regarding the regular variant-adaptation of mRNA vaccines. However, to keep the report closely aligned with the primary data, we did not place strong emphasis on these broader implications.

8. Relevant background information of the study subjects should be included in the abstract to enable readers to quickly assess the reliability of the study design. To more concisely highlight the central mechanistic discovery in the abstract, the authors could state: "Single-cell RNA-sequencing revealed that this refinement was driven by somatic hypermutation in pre-existing cross-reactive memory B cells, causing a shift in their specificity towards the novel variant ('gate-shifting'), rather than the expansion of naive-derived, JN.1-specific clones."

We thank Reviewer #2 for this suggestion, and in response included key study subject information (pre-exposed, n=42) and also modified in the Abstract (lines 43-44).

9. Since T-cell responses play a crucial role in defense against viral infection, it is advisable to include corresponding evidence or discussion regarding this aspect.

We appreciate the suggestion to include T cells in the analysis. However, our study has a clear focus on B cells and antibody responses. Moreover, the emergence of immune-escape variants is primarily driven by antibody-mediated selection, and our study specifically examines how B cells adapt to this challenge through SHM. For these reasons, we did not investigate T cells responses in the present study. We now discuss in the Limitations section (lines 398-401) of the revised manuscript that paired B and T cell analyses would provide a more comprehensive picture in future studies.

Improve the annotation of the phylogenetic trees to indicate what the branch lengths represent in Figure 3B. Corresponding methodological descriptions should be included.

The branch lengths in the clonotype dendrograms quantitatively represent the SHM load, expressed as the fraction of nucleotide deviations from the germline sequence (e.g., 0.05 corresponds to a 5% deviation from germline). This information has now been included in the Materials and Methods section (lines 626).

Several minor issues should be corrected:

line 111, superscript "8" in the caption should be verified.

This was a reference, which is now removed because it is sufficiently covered elsewhere.

"The cohort received a median of 4.5 previous COVID-19 vaccinations, and 87.8% of the included individuals reported at least one prior SARS-CoV-2 infection (Table S2)." Table S2 should be Table S1.

Corrected as suggested.

vacccination in Table S1should be corrected to vaccination.

Corrected as suggested.

Reviewer #3

The manuscript by Stankov et al., entitled "Somatic hypermutation after JN.1-adapted vaccination repurposes ancestral memory B cells for future virus variant neutralization" characterized the antibody specificity of circulating neutralizing antibodies and memory B cells followed by JN.1-adapted mRNA vaccine. The authors aimed to dissect how immunological imprinting by ancestral mRNA vaccines is overridden by the JN.1 booster. Serological analysis confirmed the robust induction of antibody responses to JN.1 and its descendent strains, such as KP.2, KP.3, KP.3.1.1, and XEC, in the analyzed cohort. Longitudinal tracking of memory B cell specificity demonstrated the gain of JN.-1-reactivity over time. In-depth analysis by scRNA-seq failed to show the phenotypic changes of memory B cells, but revealed the several BCR clonotypes showing the specificity shift from Wuhan to JN.1 by introducing SHMs. As cited, SHM-driven modification of memory B cell specificity is shown to override immunological imprinting by ancestral mRNA vaccines after repeated Omicron BA.5 exposure, but not by more recent JN.1 vaccine. The detailed analysis of JN.1-boosted memory B cell responses at monoclonal levels remains limited in my view, making the topics interesting and timely for the readership of this journal. The followings are the list of my suggestions.

We thank Reviewer #3 for the excellent summary and for highlighting that the literature lacks detailed investigations of JN.1-boosted MBC response.

Comments:

1) The phrase "future virus variant neutralization" in the title seems overreaching to me based on the data presented in the current manuscript. Whereas JN.1-adapted vaccination shifted the antibody and memory B cell specificity to JN.1 and its descendent variants, the data do not demonstrate the reactivity to unrelated variants which may emerge in the future. I suggest the revision of the title.

We thank Reviewer #3 for this notification. We fully agree that the term "future virus variant" may be an overstatement and therefore moderated our conclusion to the JN.1 antigenic space. The title was reformulated accordingly.

2) *There is a mismatch in the virus strains used in Figure 1. The authors used KP.2, KP.3, and XEC variants for detecting RBD-binding antibody titers (Figure 1E-G), and KP.3, KP.3.1.1, and XEC (not KP.2) for NT titers (Figure 1J). However, the ratios (NT/binding titers) in Figure 1K were somehow calculated using KP.2, KP.3, and XEC. I do not understand how this is possible.*

We thank Reviewer #3 for highlighting this inconsistency, which escaped our attention. The ratio was calculated using existing KP.2 neutralization titers, which we measured, but initially we did not include these values in Fig. 1J. We have now included the neutralization of KP.2 in the revised Fig. 1. On the other hand, we removed the "neutralization efficiency" ratio as requested by Reviewer #2 (see comment 1 above).

3) KP.3.1.1 appears to be less immune evasive than KP.3, XEC, and its parent JN.1 strain based on NT titers in Figure 1J. Please explain why.

We thank Reviewer #3 for highlighting this important observation. Reviewer #3 is correct in noting that KP.3.1.1 would be expected to exhibit greater immune evasion than JN.1 due to additional mutations in neutralization-relevant epitopes of the spike protein, as supported by recent literature[2,3]. To address this point and rule out batch effects, we repeated the entire set of experiments. The newly generated data confirm that KP.3.1.1 is indeed slightly more immune evasive than JN.1 and KP.3, although somewhat less so than XEC. Considering these results, we revised Figure 1J accordingly.

4) BCR clonotype analysis in Figure 3 is one of core data in this manuscript for supporting the presence of "gate shifter" at BCR gene levels. However, there are not sufficient information in sequence data of the analyzed BCR in the current format, making it hard to interpretate the results as claimed by the authors. The authors need to present the BCR sequence data of all clones belonging to CT1, CT2, CT3, CT4, and CT5 (Figure 3B) and clarify the positions of SHM, the names of VDJ/VJ gene template, and CDR3 sequences. These BCR sequencing data need to be deposited in public database with the specific clone names as well.

We agree that the sequence data provided in our original submission were insufficient. We now provide complete BCR sequence annotations for all single cells from our dataset that were expressed as monoclonal antibodies. In addition, all VH and VL SHM, as annotated by IgBlast, are now explicitly highlighted. These sequences are compiled in Supplementary Data 2. Furthermore, to ensure reproducibility of our bioinformatic analyses, we have deposited the complete set of V(D)J raw data in the SRA under accession number PRJNA1431958. We have also submitted the sequences of newly identified mAbs to GenBank under accession numbers PZ121777-PZ121840, making them fully accessible for reuse, e.g., through BLAST-based BCR sequence comparisons. The Data availability statement has been updated accordingly (lines 690-695).

5) *Related to comment 4, I cannot understand which clones are CT2-1/-2/-3/-4/-5/-6/-7 in clonal tree of Figure 3B. The same is true for CT5 clones. I would like to see the aligned sequence data with information on SHM, V gene templates, and CDR3 sequences.*

We thank Reviewer #3 for highlighting the insufficient labelling of the clonal trees, which was now corrected in Fig. 3B. The aligned sequences of CT5 are shown in Fig. 4A, in which all SHM, V gene segments, and CDR3 sequences are indicated. Since the purpose of this alignment was the comparison of CT5-2 to Tixagevimab, COV2-3025, R259-1B9, C043, and 17T2, we did not perform such an alignment for the other clonotypes. Nevertheless, for full transparency all sequences are now provided in Supplementary Data 2 and were also deposited in GenBank (accession numbers PZ121777-PZ121840).

6) *CT3 clones failed to bind spike, S1 and RBD. I assume that they are not neutralizers. CT4 clones bound to spike and S1, but not RBD, and they were non-neutralizers (Figure 3). Because the authors cannot exclude the possibility that the CT3 and CT4 are non-specific clones which are contaminated in FACS-sorting B cells at certain levels, I strongly suggest that these clones should be excluded from the analysis. I also suggest to reconsider the following description in line 178 "For the RBD, CT4 did not exhibit any binding, presumably because it was unable to bind to RBD alone in the absence of other non-RBD domains", since CT4-3 clone was isolated as JN.1 RBD binder in flow cytometry. Without showing the RBD binding affinity above the detectable levels using more sensitive methods, such as biolayer interferometry, the authors cannot conclude that CT3 and CT4 clones are indeed vaccine-induced clones (they are more likely non-specific clones).*

We thank Reviewer #3 for the detailed discussion of CT3 and CT4. A similar point was raised by Reviewer #2 (comment 5, see above). In response, we followed the reviewers' suggestion to apply more sensitive methods. Using an Octet R8e biolayer interferometry (BLI) system, we detected that CT3 indeed binds the RBD with low affinity (see revised Fig. S5). Despite this low affinity, the corresponding MBC can still be labeled with fluorescent tetramers because multivalent antigen can cross-link several BCRs on the cell surface, thereby increasing avidity. For CT4, our BLI measurements did not detect measurable binding to the isolated RBD (data not shown). However, all CT4 clones strongly reacted with the spike trimer and the S1 monomer in ELISA. We therefore concluded that CT4 likely represents a high affinity clonotype that recognizes an epitope within the spike protein

that is not sufficiently represented by recombinant, monomeric RBD alone. In this case again, labelling of MBC with a multivalent RBD-tetramer may still occur due to avidity effects. Taken together, our newly generated data indicate that neither CT3 nor CT4 represent background signals, and we therefore kept them in the dataset. Our ELISA-based follow-up experiments (comparing binding to seven recombinant RBD variants) were limited to CT1, CT2 and CT5. We believe that the heterogeneous binding behavior observed across clonotypes reflects the biological diversity that can be expected among memory B cells. Rather than excluding such cases, we chose to present them transparently to the readership, including examples of low-affinity binding (as CT3) and unexpected binding patterns (as for CT4).

7) The authors did not prepare the germline-reverted mAbs from CT2 and CT5. However, an additional importance piece of data supporting the gate shifter phenomenon is to show Wuhan-specific binding and/or NT activity in the ancestral antibody clones encoded by germline VH/VL genes.

We thank Reviewer #3 for this important question. We generated the corresponding germline reverted antibodies and included their ELISA binding profiles in the revised Fig. 3H. In both cases, the germline variants bound only to the Wu01 RBD. This observation strongly supports the idea that these antibodies originally emerged as close-to-germline sequences recognizing the ancestral antigen, e.g., during the initial vaccine response against the Wuhan strain. Their binding and neutralization breadth (as visualized in Fig. 3E+F) appears to have been acquired only after accumulation of SHM. While not directly demonstrated, our results strongly suggest that the JN.1-adapted mRNA vaccination and/or previous vaccinations and breakthrough infections may have contributed to the selection of these SHM.

Reviewer #4

The manuscript by Stankov et. al. investigated how JN.1-adapted COVID-19 vaccination modulates antibody and memory B cell immunity in response to ongoing SARS-CoV-2 antigenic drift. The authors showed that the adapted vaccine boosts IgG binding and neutralization against JN.1 and related variants. Longitudinal analyses seemed to reveal a progressive shift in memory B cell specificity, with an increase in exclusively JN.1-reactive cells and a decline in Wu01-cross-reactive populations. The authors also used scRNAseq data to support their observations.

The experiments appear carefully performed and the results were mostly clear. Understanding how variant-adapted vaccines repurpose pre-existing memory B cells has significant implications for developing next-generation COVID-19 vaccines capable of countering ongoing SARS-CoV-2 evolution. However, there are numerous points that impact the data analysis and presentation.

We thank Reviewer #4 for the careful evaluation of our manuscript and for the constructive comments regarding data interpretation, flow-cytometry rigor, and mechanistic clarity.

Specific points

1. *The presented data is only for IgG responses. Were IgM responses to JN.1 and other recent variants assessed? If IgM responses are weak, that would further support the interpretation that the B cells reacting exclusively to JN.1 originate from pre-existing MBC.*

We thank Reviewer #4 for the idea of using IgM responses to generate another level of evidence for the dominant recall of MBC rather than *de novo* recruitment of naïve B cells. Correspondingly, we experimentally assessed IgM responses. IgM binding to JN.1, KP.2, and KP.3 was measured in pooled sera (pool of n=42 at day 0; pool of n=39 at day 21). IgM titers were marginal at baseline and unchanged after vaccination, in contrast to enhanced IgG responses. The absence of IgM induction argues against substantial naïve B-cell recruitment within the 2–3-week window and supports recall and maturation of pre-existing, already class-switched memory B cells. Figure S3, Methods (line 471), Results (lines 200-202), and Discussion (line 344-350) were updated accordingly.

2. In Figure 1E, the authors compare raw OD450 values across ELISAs with different antigens (and/or plates). This is problematic, as OD450 is assay-, plate-, and antigen-dependent, and small differences in coating, incubation, or substrate development can substantially affect readings. It is unclear what methods were used to account for these confounders, such as standard curves or duplicate wells or if there was a normalization procedure. The data in Figure S1 showing calculated relative binding units based on standard curves appear more reasonable. Although the results show a trend, it is not as pronounced as presented in Figures 1E–G. This discrepancy suggests selective data presentation that does not seem justified. Full curve ELISA results that give ED50 or endpoint titers, with methods to normalize / standardize across plates would be appropriate; alternatively, comparisons can be restricted to the same assay/plate. Consider moving Figure S1 data to the main figures and relocating Figure 1E–G to the supplement. Do all figures focus exclusively on comparisons between JN.1 and other variants? For example, in Figure 1G, the differences between Wu01 and the other recent variants also appear substantial. The authors should clarify the statistical methods used for each comparison, ideally in the figure legends.

We agree that comparing single-point OD450 values across ELISAs with different antigens/plates is not appropriate and can be confounded by saturation, antigen-dependent signal scaling and plate-to-plate variation. A similar question was raised by Reviewer #2 (comment 1). We therefore re-performed the entire multi-variant ELISA dataset as full serum titration curves (≥8 dilution steps per sample/antigen, 4 separate, independent experiments) and reanalyzed binding using

standardized nonlinear regression. Fig. 1 was substantially revised, and changes were introduced in the Results section (lines 118-128). The statistical evaluation was also revised and in addition to optimized figure legends, we provided a source data file that includes all detail about the statistical testing for each figure panel.

3. In Figure 1K, this metric seems contrived, as NT50 and ELISA OD450 represent distinct and non-comparable units. The biological interpretation of the ratio is of unclear value, and the index does not appear to be standardized across assays. And as mentioned above, presenting the data solely as raw OD450 values is problematic, let alone deriving a ratio based on those measurements.

We fully agree, and this point was also raised by Reviewer #2 (comment 1). Correspondingly, we removed this kind of data representation.

4. *In figure S2, there are two concerns regarding the gating strategy.:*
A) In the first gating step, it is unclear why not all cells are included. After B-cell proliferation, cells become larger and more granular (FSC/SSC increase), so excluding cells at this stage may omit relevant populations.

We thank Reviewer #4 for the detailed inspection of the gating strategy. We agree and have broadened the FSC/SSC gate to include activated/blastoid lymphocytes. Re-analysis recovered a small additional fraction of tetramer-positive B cells, but did not alter overall frequencies, trends, or statistical conclusions. Revised gates are shown in Figure S2 and documented accordingly in the Methods.

It is unclear why antibody-secreting cells (ASCs) are excluded prior to gating B cells. If ASCs refer to CD27+CD38high cells, the commonly used term would be plasmablasts. The gating shown for CD27 and CD38 plot appears inappropriate, excluding CD27+CD38high cells before gating B cells. Plasmablasts express surface BCR capable of recognizing the baits and represent an important component of antigen-specific B cells that should be included in the analysis. The current gating strategy raises concerns about the reliability of the results. I recommend revisiting the gating strategy to ensure that all relevant B cell populations, including plasmablasts, are properly captured.

We agree. The gating strategy was revised to explicitly include plasmablasts ($CD27^{+}CD38^{hi}$ $CD20^{low/-}$) alongside IgD^{-} memory B cells. Tetramer staining is now analyzed in both compartments. The inclusion of plasmablasts reinforced rather than contradicted the observed trends. Updated gating is shown in Figure S2 of the revised manuscript.

5. Page 6, lines 129-132: Overall, Wu01-specific B cells still constitute the majority, while single JN.1-reactive and cross-reactive populations remain much smaller. This does not appear to represent a qualitative shift in preferred antigen specificity. I also have a concern regarding the tetramer staining: have the authors tested negative control samples (e.g., PBMCs from healthy donors prior to the COVID-19 pandemic) to assess background staining? Non-specific binding is an important consideration, especially since cross-reactivity between SARS-CoV-2 and common human coronaviruses has been reported.

We agree that Wu01-reactive memory B cells remain the dominant population at all time points. Correspondingly, we do not claim a population-wide specificity switch. In response, we revised the text to describe a modest, subset-restricted rebalancing toward JN.1 binding. For tetramer specificity, we are glad to report that due to our unique study design, we had cryopreserved pre-pandemic PBMCs from a subset of the analyzed donors, which allowed us to define background labeling and to set fixed Wu01/JN.1 thresholds, which were then held constant across all samples (see revised Fig. 2B). Of note, background signals were minimal.

6. Lines 132-134: This may represent an overstatement of the data. It is also possible that some of the observed responses reflect de novo induction of new B-cell clones. Notably, the percentage of Wu01 single-reactive cells on day 12 shows a declining trend, and the absolute numbers of these cells are much higher than the other two populations. The frequencies of JN.1-reactive cells, whether single-antigen or cross-reactive, are not constant over time. Moreover, the lack of absolute B-cell counts limits the ability to draw definitive conclusions about shifts in population size or antigen specificity.

We agree with this point and have tempered our interpretation throughout the manuscript. We now report normalized event counts in addition to frequencies, based on highly standardized acquisition conditions with consistent singlet numbers across all time points. Although these measurements do not represent bead-calibrated absolute counts, paired analyses yield concordant results. We now explicitly state that a modest *de novo* contribution cannot be excluded. However, the data overall favor maturation of pre-existing MBC. This interpretation is supported by high and stable SHM levels without evidence of near-germline influx, improved JN.1-specific monoclonal antibody kinetics, and minimal IgM induction (now shown in newly added Fig. S3C).

7. Lines 154-155: Have the authors performed a pseudotime/cell trajectory analysis for these B-cell populations? Such an analysis could provide insights into the developmental relationships between Wu01-specific, JN.1-specific, and cross-reactive B cells, and clarify whether observed shifts in frequency reflect maturation, clonal expansion, or recruitment of new clones over time.

We thank Reviewer #4 for the suggestion to perform pseudotime analysis. In response, we conducted an scVelo trajectory analysis (see Methods, lines 629-634). Interestingly, clusters 3 and 4 represent less differentiated states, and clusters 1 and 2 correspond to more differentiated states along the inferred pseudotime trajectory. When annotating the cell barcodes used for mAb expression based on their membership in expanded clonotypes containing gate-shifting antibodies (see Fig. 3B), we observed that all gate shifters were located within the more differentiated clusters, which were advanced in pseudotime. This pattern suggests that the gate shift may occur during, or towards the end of, a differentiation process. These new results have been added to Figure S4J and are presented there alongside the other transcriptomic analyses.

8. Lines 159-163: If both processes, maturation of pre-existing clones and de novo induction of new B cell clones, occur simultaneously and at similar levels, could this also produce the observed patterns?

Theoretically, in such a scenario the mean SHM load could stay constant, but importantly we would expect the distribution to be split into a bimodal distribution, which we did not observe in Fig. 2G. Furthermore, we now assessed IgM responses against JN.1 and could not detect induction upon vaccination (for reference see comment 1 above), strongly suggesting that the *de novo* induction of naïve B cells clones is very limited (see Fig. S3C).

9. In Figure 3D, as noted earlier, OD450 values can be influenced by many factors. I recommend presenting these data using EC50 or endpoint titers. And just to clarify, does 'WT' in Figures 3 and 4 refer to Wu01? The labeling should be kept consistent throughout the figures.

Agreed. All OD450-based readouts were replaced with EC50/ED50 values derived from full titrations. "WT" has been standardized to Wu01 throughout figures and text.

10. In Figure 3, it is possible that the JN.1-reactive clones captured in this gate have higher avidity, which could make them more likely to be recognized by the bait. This is an interesting possibility, and measuring BCR avidity could provide valuable insight to support your observations.

We thank Reviewer #4 for suggesting the aspect of affinity vs. avidity, which is an important consideration when comparing (cellular) MBC responses with monoclonal antibody binding

measurements. During revision, we addressed this by measuring Fab-level binding kinetics by BLI, which eliminates multivalent avidity. Gate-shifting antibodies show lower KD to JN.1, driven by faster k_{off} and often faster k_{on}, compared with clonally related cross-reactive antibodies (see Fig. 4D, Fig. S5, and Supplementary Data 1). These intrinsic kinetic advantages explain preferential tetramer capture. Importantly, the binding behavior of tetramerized streptavidin-based antigen probes will be influenced again by avidity effects (tetramers are able to interact with multiple BCR on the surface of the same MBC simultaneously).

11. Figure 3G, how was the cutoff for this gate shifting zone determined? These cutoffs appear to be arbitrary to suit the story. If it is a real metric, it should be consistent across different clones, or at least have a rationale for the shaded areas.

We agree that this could be misinterpreted. The shaded area was purely illustrative and not used for classification or statistical analysis. We restructured the representation of Fig. 3G, and in this process we eliminated also the concept of a gate-shifting zone.

Minor comments:

1. In Table S2, the authors should specify the types of vaccines received by the participants to provide clarity and context for the reported responses.

Participants received an updated monovalent mRNA Omicron JN.1 vaccine (Bretovameran/Comirnaty Omicron JN.1, BioNTech-Pfizer; 30 µg) as the study vaccination. The information was added to Table S2, as suggested.

2. The authors should include a dedicated Statistical Analysis section in the Methods to describe all analyses performed. Additionally, the statistical tests used for each figure should be clearly indicated in the corresponding figure legends.

We followed this advice and described the statistical analysis in a separate section (lines 654-681).

3. Page 5, lines 96-97: "Consequently, a pronounced…the COVID-19 pandemic was observed." was repeated, which also mentioned in lines 91-93.

We apologize for the sentence duplication. The section was rewritten due to improved experimental data (ED50 titration instead of OD450 values), and the duplication was removed in this process.

4. Line 109: The statement 'all tested variants increased' is inaccurate, as XBB.1.5 did not show a statistically significant increase. Additionally, in Figure 1J, it is unclear whether the titers and fold changes shown above the figure represent mean/median values; the authors should clarify this and ensure precise reporting.

We thank Reviewer #4 for highlighting this aspect. Fig. 1, and in particular Fig. 1J, was restructured in response to comments of Reviewer #2 (comment 1) and Reviewer #3 (comments 2+3). We also ensured that this information is included in the figure legends of the revised manuscript.

5. In Figure 1J, I noticed that the fold changes for XEC in the cited study do not match what is reported here. I'm just curious why this is the case, especially since it appears to be from the same CoCo cohort.

We thank the reviewer for carefully cross-checking the values. We would like to clarify that there was no discrepancy in the originally submitted manuscript. The fold change for XEC reported in the cited study (PMID: 39522531; Supplementary Fig. F)[4] was 11.2-fold, which was identical to the value presented in our original Figure 1J. As both analyses derive from the same CoCo cohort, this concordance confirms consistency between the two reports.

Nevertheless, Figure 1J has been revised in the revised manuscript. It has been reframed to display fold changes in neutralization titers, separately for pre- and post-vaccination timepoints, using XBB.1.5 as the designated reference comparator. Fold changes are now displayed in Figure 1K and depict median fold changes on the participant level, as opposed to geometric mean titers in the cited study.

6. The results sections lack concise summaries, and the logical transitions between experiments are not fully supported. For example, at the end of the first results section (lines 115–120), the text is wordy and could be more clearly and succinctly presented.

We thank Reviewer #4 for highlighting this aspect, which is important feedback for us. Since substantial parts of the Results section were reformulated, this aspect should be resolved in the revised version of the manuscript.

7.Lines 125-126: This may leave readers uncertain whether the first two populations are exclusive to a single antigen or could include cross-reactive cells. I recommend explicitly stating that the first two populations are reactive only to a single antigen, for example: We classified B cells as Wu01 S-specific or JN.1 RBD-specific, representing cells reactive exclusively to a single antigen, and also identified cross-reactive B cells that were labeled by both antigens.

We have followed the advice and stated this point more clearly in the results section (lines 151-153).

8. Page 6, lines 126-128: The current wording implies that B-cell frequency inevitably increases after vaccination. This seems overly absolute.

We agree and have reformulated the section accordingly (lines 159-160).

9. Line 148: It should be day 13 instead of 14. The same in Figure S4B.

We thank Reviewer #4 for highlighting these errors and corrected them accordingly.

Reviewer #5

I co-reviewed this manuscript with one of the reviewers who provided the listed reports. This is part of the Nature Communications initiative to facilitate training in peer review and to provide appropriate recognition for Early Career Researchers who co-review manuscripts.

We thank Reviewer #5 for their contribution and participation in the co-review process.

References

1. Korenkov, M., Zehner, M., Cohen-Dvashi, H., Borenstein-Katz, A., Kottege, L., Janicki, H., Vanshylla, K., Weber, T., Gruell, H., Koch, M., et al. (2023). Somatic hypermutation introduces bystander mutations that prepare SARS-CoV-2 antibodies for emerging variants. Immunity *56*, 2803-2815.e2806. 10.1016/j.immuni.2023.11.004.
2. Feng, Z., Huang, J., Baboo, S., Diedrich, J.K., Bangaru, S., Paulson, J.C., Yates, J.R., 3rd, Yuan, M., Wilson, I.A., and Ward, A.B. (2025). Structural and functional insights into the evolution of SARS-CoV-2 KP.3.1.1 spike protein. Cell Rep *44*, 115941. 10.1016/j.celrep.2025.115941.
3. Kaku, Y., Uriu, K., Okumura, K., Ito, J., and Sato, K. (2024). Virological characteristics of the SARS-CoV-2 KP.3.1.1 variant. The Lancet Infectious Diseases *24*, e609. 10.1016/S1473-3099(24)00505-X.
4. Arora, P., Happle, C., Kempf, A., Nehlmeier, I., Stankov, M.V., Dopfer-Jablonka, A., Behrens, G.M.N., Pöhlmann, S., and Hoffmann, M. (2024). Impact of JN.1 booster vaccination on neutralisation of SARS-CoV-2 variants KP.3.1.1 and XEC. The Lancet Infectious Diseases *24*, e732-e733. 10.1016/S1473-3099(24)00688-1.

Point by Point Reply

JN.1-adapted vaccination is associated with readjustment of ancestral memory B cells toward neutralization within the JN.1 antigenic space

Reviewer #1

The authors have satisfactorily addressed all of my concerns. I recommend that the manuscript be accepted for publication.

We thank Reviewer 1 for the positive assessment and the recommendation for acceptance.

Reviewer #2

The revised manuscript is significantly improved and addresses most prior concerns raised in the previous round. The authors also appropriately softened some of the causal language and now acknowledge several limitations more explicitly. However, the study still has substantial design and interpretation issues that limit the strength and generalizability of its conclusions.

We thank Reviewer 2 for the thorough and constructive evaluation of the revised manuscript. We have addressed all six major comments in detail below. The revisions include new multivariable linear regression (ANCOVA), comprehensive quantitative reporting, systematic language moderation, and additional contextualizing discussion. We believe that the revised manuscript is now consistent with the observational nature of the study design.

1. Cohort heterogeneity is a fundamental design limitation that directly affects the main conclusion. The study cohort is highly pre-exposed, with a median of 4.5 prior COVID-19 vaccinations and 87.8% of participants reporting at least one prior SARS-CoV-2 infection. In such a cohort, the observed B-cell repertoire features, including the proposed gate-shifting phenomenon, are difficult to attribute specifically to the current JN.1-adapted booster rather than to prior infection, prior variant-adapted vaccination, or cumulative antigenic exposure. The manuscript reports inverse correlations between baseline binding or neutralization titers and post-vaccination fold changes, which the authors interpret as ceiling-limited boosting in this highly pre-exposed cohort. This is a reasonable interpretation, but it also means that pooled fold-change analyses are difficult to interpret biologically unless the cohort is stratified by baseline immunity, recent infection, or last exposure type. Without such analyses, it remains unclear whether observed boosting patterns reflect booster-specific immune refocusing or simply differences in starting immune state. The authors acknowledge this limitation, but it should be treated as a central design constraint rather than a secondary caveat. The authors are recommend to conduct exploratory subgroup analyses based on detailed infection and vaccination history (which should also be provided in detail) to compare immune responses across participants. Furthermore, causal language should be moderated throughout the manuscript.

We thank the reviewer for this important comment and address it at two levels: 1. the inevitability of cohort pre-exposure by the time the JN.1 vaccine became available, and 2. the new multivariable regression analyses (ANCOVA) in which post-vaccination antibody levels were modelled as the outcome with log-transformed baseline titer as a covariate alongside number of prior vaccinations, months since last vaccination, and number of prior SARS-CoV-2 infections, allowing the independent contribution of each exposure variable to be assessed while holding the others constant, across all

immunological outcome measures, to directly test whether prior immune history confounds our conclusions.

We agree with the reviewer that cohort heterogeneity is a challenge. Although the reviewer considers this a major limitation, we would like to emphasize that the primary aim of our study was to assess the impact of Omicron variant-adapted vaccinations. This inevitably requires cohorts with complex antigen exposure histories due to infection or vaccination after the Omicron wave. The Comirnaty JN.1 vaccine received EMA authorization in July 2024, more than four years into the pandemic. By this time, the global population carried heterogeneous immune histories derived from various exposures to infection, viral variants, and vaccination. Constructing a comparator cohort with minimal prior exposure at this time point would have been practically impossible.

An earlier study time point was also not scientifically feasible for the question we ask. The JN.1 lineage descends from BA.2.86, which harbors more than 30 additional spike mutations compared with XBB subvariants[1]. Antigenic cartography established a five- to six-fold antigenic difference between JN.1 and XBB.1.5[2]. Our central question, namely whether the JN.1-adapted booster is associated with re-focusing of memory B cell responses within the JN.1 antigenic space, is only biologically meaningful in the context of this substantial antigenic step and could not have been investigated before the JN.1 vaccine existed.

However, such cohort heterogeneity also demands careful evaluation of the potential impact of confounders on our results. The reviewer requested a more cautious statistical consideration of baseline immunity, recent infection, and last exposure type when comparing immune responses across participants. Rather than performing an exploratory univariable subgroup analysis, which would further reduce statistical power by splitting an already modest cohort, we conducted an analysis of covariance (ANCOVA). Specifically, we assessed the impact of baseline antibody levels, number of prior vaccinations, months since last vaccination, and number of SARS-CoV-2 infections on post-vaccination antibody levels, using matched baseline titers as a covariate - day 0 ED50 for ED50 outcomes and day 0 NT50 for NT50 outcomes - thereby ensuring that the post-vaccination response for each assay was evaluated against its own pre-vaccination baseline within the same assay system. Antibody responses were measured by ELISA (ED50) and neutralizing antibody titers (NT50) against the XBB.1.5, JN.1, and XEC variants.

This ANCOVA confirmed our previously described correlation between baseline binding or neutralization titers and post-vaccination titers. In addition, time since last vaccination showed a statistically significant association with post-vaccination ED50 and NT50 against XBB.1.5, as well as NT50 against JN.1.

In summary, the ANCOVA provides additional evidence that baseline antibody levels predominantly determine post-vaccination responses, and that time since last vaccination, which strongly influences baseline antibody levels, has an additional effect. Although we cannot exclude potential effects of the number of prior vaccinations and/or SARS-CoV-2 infections, the ANCOVA did not provide evidence that these were relevant factors in our cohort.

Detailed participant characteristics including subgroup assignments have been added to Supplementary Data 3. The ANCOVA results are provided in the Results section (line 142-150) and Supplementary Data 3. Cohort pre-exposure is now explicitly framed as a central design constraint in the Discussion (line 385-394). Causal language has been moderated throughout as detailed in the response to Major Comment 2.

2. The revised title is more appropriately cautious, which is a welcome change. However, portions

of the abstract, results, and discussion still risk implying that the JN.1 booster itself drove the gate-shifting phenomenon. Given the observational design and the authors' own acknowledgment that they cannot definitively distinguish booster-induced effects from prior exposure-driven maturation, the language throughout should remain consistently associative.

We thank Reviewer #2 for this comment and agree that consistent associative framing of the JN.1-adapted vaccination is more appropriate, given the observational study design. We have reviewed the manuscript and identified the following instances where causal language remained, replacing each with appropriately associative phrasing. The specific changes are as follows.

In the Abstract, the concluding sentence "JN.1-adapted booster vaccination refines pre-existing MBC repertoires toward the JN.1 antigenic space, enhancing neutralization" has been changed to "JN.1-adapted booster vaccination is associated with refinement of pre-existing MBC repertoires toward the JN.1 antigenic space and with enhanced neutralization".

In the Results section, "this was primarily driven by an increasing proportion of predominantly JN.1-only B cells" has been changed to "this was primarily reflected by an increasing proportion of predominantly JN.1-only B cells". The phrase "mechanistically explain the emergence of JN.1-only cells" has been changed to "are consistent with the emergence of JN.1-only cells". The phrase "SHM that leads to a transition of certain clones, effectively updating their specificity" has been changed to "SHM in a subset of clones, consistent with progressive adaptation of their specificity." The phrase "supporting the theory that they occur as a result of a differentiation process" has been changed to "consistent with their occurrence through a differentiation process".

In the Discussion section, "the vaccine induced serum antibodies that specifically neutralized the JN.1 variant" has been changed to "vaccination was associated with serum antibodies that specifically neutralized the JN.1 variant." The phrase "SHM in pre-existing MBC is, at least in part, responsible for the improved neutralization" has been changed to "SHM in pre-existing MBC is, at least in part, associated with the improved neutralization observed following JN.1-adapted booster vaccination". The phrase "SHM-driven repertoire refinement" has been changed to "SHM-associated repertoire refinement".

3. The scRNA-seq analysis was performed in only 7 participants selected as representative based on serum antibody titers and neutralization capacity. However, representativeness in serology does not necessarily imply representativeness in exposure history or clonal immune architecture. The manuscript would be strengthened by a clearer justification of the sampling strategy and a more explicit discussion of how this selective subsampling may bias conclusions about the prevalence and biological relevance of gate-shifting clonotypes.

We thank the reviewer for this important methodological point. The downsampling to seven donors for the scRNA-seq analysis was a necessary step to control the sorting time (sorting of the rare antigen-specific B cells took several hours and by including more donors the cell viability would have been at risk). Furthermore, the experimental costs of the 10X Genomics kits and the NGS sequencing necessitated the selection of a smaller number of donors.

The selection criteria included pre-exposure variables. At the time of enrolment, the seven donors selected for scRNA-seq analysis were not statistically different from the rest of the cohort on all pre-exposure variables: number of prior vaccinations (median 4 vs. 5, Mann-Whitney U p = 0.906), number of prior infections (median 1 vs. 1, p = 0.358), months since last vaccination (median 10.8 vs. 10.8, p = 0.905), and Omicron infection proportion (85.7% vs. 86.7%). Obviously, neither serology

data nor the clonal B cell architecture were available before vaccination, so these were not considered as inclusion or exclusion criteria for cell sorting.

Critically, to assess whether our selection introduced a bias concerning the main conclusions, we assessed retrospectively whether there are meaningful differences in the serology data between the selected individuals and the remainder of the cohort, as correctly pointed out by Reviewer #2. In response to comment 3, we also compared the antigen-reactive MBC distributions of the 7 sorted donors against the remaining participants directly. The proportions of Wu01-only, JN.1-only, and Wu01/JN.1 cross-reactive MBCs at day 0, day 13, and day 21 followed a very similar pattern between the sorted donors and the rest of the cohort at all three time points (see Fig. 1 below). This confirms that the 7 selected donors were not only representative in terms of prior exposure history but also in terms of the very B cell response phenotype that the scRNA-seq was designed to characterize. The concern that the selection might introduce systematic bias is therefore not supported by the data. Detailed participant characteristics including vaccination and infection history for all 39 participants are provided in Supplementary Data 3.

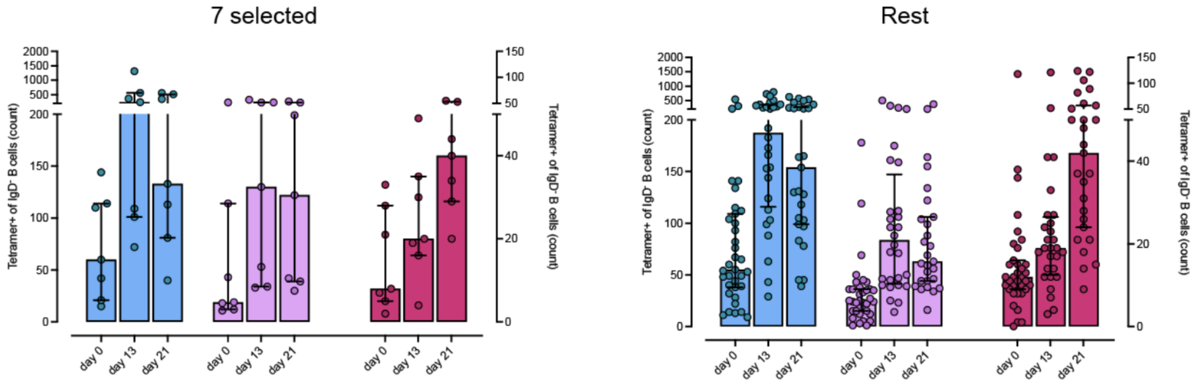

Fig. 1: Quantification of antigen-specific B cells in the seven donors selected for scRNA-seq. Left side: the sub-cohort (n=7) that was studied using scRNA-seq. Right side: The remaining donors. The figure was added to Fig. S3 of the revised manuscript.

Furthermore, the gate-shifting phenomenon is not a donor-specific artefact, because multiple gate shifters were identified in multiple donors (see response to comment 4 below). The convergent public IGHV1-58 clonotype was independently identified across multiple studies, which we compared in Figure 4 of the originally submitted manuscript. The detection of the same clonotype undergoing progressive specificity shifts in independent donors provides the strongest available evidence that gate-shifting reflects a reproducible biological process.

A paragraph describing the scRNA-seq sampling strategy has been added to the Methods section. The comparison of MBC distribution between sorted and non-sorted donors across all three time points has been added to Figure S3.

4. The manuscript notes that only a minority of cells from expanded clonotypes exhibited gate-shifting behavior. This raises an important interpretive question: is gate shifting a representative population-level mechanism, or a biologically interesting but relatively uncommon phenomenon? The authors should provide clearer quantitative reporting, including the proportion of expanded clonotypes showing gate shifting, their contribution to the total antigen-reactive MBC pool, and the consistency of this phenomenon across donors.

We thank reviewer #2 for raising the important question, whether gate-shifting is the rule or an exception. We agree that this is important information that was previously not addressed in our

analysis. We have included a more thorough quantification of the proportion of expanded clonotypes and the proportion of gate-shifting clones among the expanded clonotypes in the revised Fig. 3C (see below, Fig. 2 on the left). Furthermore, we have annotated which donors contribute to the clonally expanded clonotypes, and which donors contribute to the gate-shifting clones at what proportion. From 2049 total B cells that passed the quality filtering, 522 belonged to expanded clonotypes. Within these expanded clonotypes, 28 cells (5.36%) are classified as gate-shifters (see below, Fig. 2 on the right). There was evidence of gate-shifting in three of seven donors. Importantly, the same three donors (donors 1, 6 and 5) contributed the vast majority of clonally expanded and also the majority of all cells in the experiment. Due to the limited sampling depth in peripheral blood, their true number is likely underestimated, and it is very plausible that if more cells had been retrieved from the remaining donors, more clonally expanded B cells would have been detected, and then also gate-shifters could have been detected. This analysis is provided in the revised Supplementary Figure 4K of the manuscript. Therefore, our data are consistent with the concept that gate-shifting is a reproducible and biologically meaningful process, detectable even within the constraints of peripheral blood sampling.

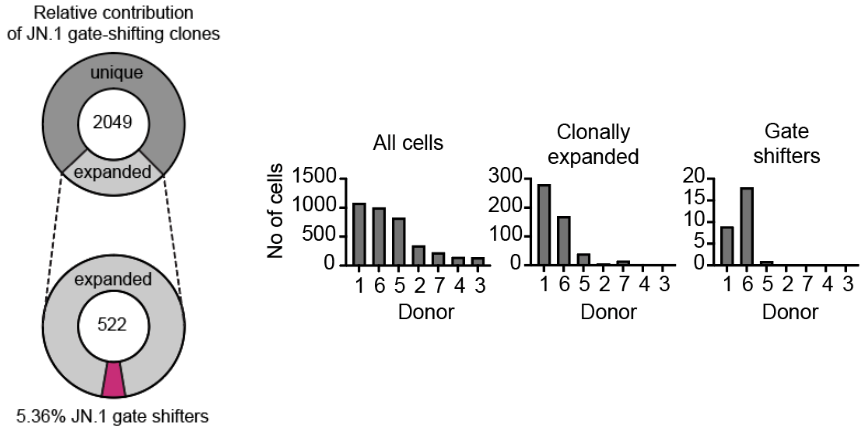

Fig. 2: Quantification of gate-shifters. Left: Quantification of expanded clonotypes among all clonotypes in the V(D)J dataset (upper chart) and contribution of gate shifters among all expanded clonotypes (lower chart). Right: The number of all cells, clonally expanded B cells and gate shifters resolved for donor numbers as determined by Souporcell.

5. Broader conclusions about vaccine strategy are somewhat ahead of the data. The discussion extends toward implications for iterative vaccine updating, breadth, and correlates of protection. While this is reasonable to discuss, the study does not assess T-cell responses, which are also relevant to SARS-CoV-2 protection. In the absence of such data, claims about broader vaccine strategy or immune correlates should be moderated further.

We thank the reviewer for this important point and agree that vaccine strategy conclusions must be qualified in the absence of T cell data. We have made the following specific changes to the Discussion:

The sentence supporting iterative vaccine updating has been revised by the amendment "although formal assessment of this strategy would require integration of T cell responses, clinical endpoints, and correlates of protection that were not determined in the present study".

The statement that SHM-driven refinement "indicates retained plasticity for clonotype adaptation" has been moderated to "suggesting that some degree of clonotype plasticity may be retained at the B cell level following exposure to variant-updated immunogens".

6. The authors are advised to more carefully verify the data in Figure 1 and the manner of data presentation.

We thank reviewer #2 for this comment and have carefully re-examined Figure 1 and all underlying source data. We have identified one mistake in Fig. 1F, in which due to the logarithmic scaling of the Y axis, some data points accidentally were outside the plotted range. We apologize for this oversight and have corrected Fig. 1F in the revised version of the manuscript (see Fig. 3 of the point-reply below). The interpretation of the data remains identical. We are confident that all data in Figure 1 are now correctly presented.

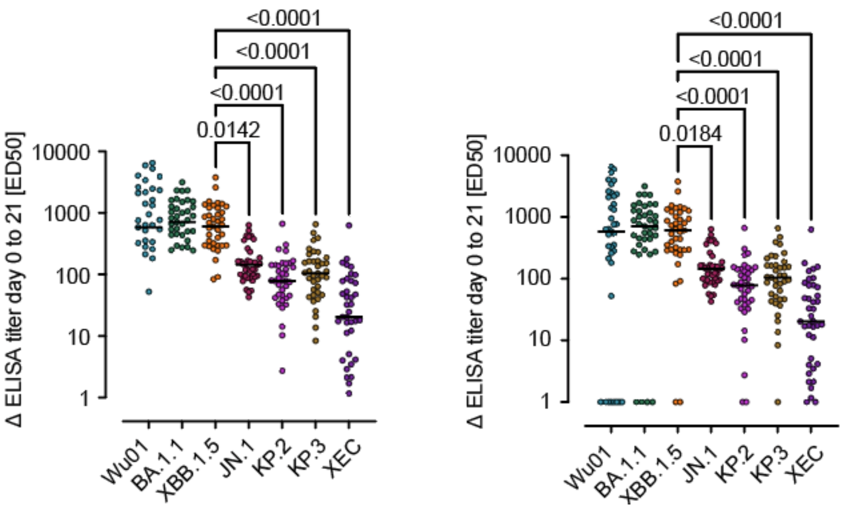

Fig. 3: Absolute increase in ED50 between day 0 and day 21 for each variant (n=39, paired). Negative values were plotted at y=1 in the logarithmic visualization. Left: Originally submitted Fig. 1F. Right: Revised Fig. 1F.

Reviewer #3

The authors revised the manuscript in sufficient way. No further comments.

We thank Reviewer #3 again for the valuable suggestions made earlier and are pleased that our revisions addressed the concerns sufficiently.

Reviewer #4

The authors have revised the manuscript in accordance with our comments. In particular, they now use ED50 for all ELISA data instead of relying on a single point OD450. They have done BLI and trajectory analysis as recommended, and have also moderated their interpretations and avoided over-interpreting the data. The manuscript has been substantially improved.

We thank Reviewer #4 for the evaluation of our manuscript and the improvements that were made possible through the suggestions.

One remaining minor issue is that assessing IgM responses using pooled sera is understandable but not ideal, as it may obscure the true variability and responses of individual samples. However, this is a minor point and does not preclude inclusion of those data.

We agree that in principle, pooling of sera obscures the variability of the individual samples in the pool. However, individual serum volumes were insufficient to generate full serial dilution curves for IgM quantification. Because IgM responses to booster vaccination in highly pre-exposed individuals are expected to be very low, reliable ED50 determination from individual titration curves would have required substantially larger serum volumes per donor than were available after allocation to the primary serology and neutralization assays.
Critically, the same pooled sera used for IgM detection were run in parallel with the IgG assay using an anti-IgG secondary antibody. The IgG signal in the pooled sera was robust and showed clear increases from day 0 to day 21, demonstrating that the pooled samples were immunologically representative of the cohort and that the assay format was sensitive to post-vaccination changes in this material. The absence of a detectable IgM response in the same pooled samples therefore most likely reflects a true biological result and the parallel IgG signal serves as an internal control for the validity of the pooled approach and is shown alongside the IgM results in Supplementary Figure S3. Furthermore, the IgG responses were analyzed on the level of individual samples in Fig. 1E-G.

Additionally, in Figure S2, the gating strategy contains incorrect labels on the top, which should be corrected.

We thank Reviewer #4 for drawing our attention to the unclear labels in the gating strategy in Figure S2. The labels in the red boxes above each plot identify the parental gate from which the displayed events were derived, following a standard flow cytometry convention. To eliminate any ambiguity, we have added the following clarifying sentence to the Figure S2 legend: "The label in the red boxes above each plot identify the parental gate from which the displayed events were derived; the gate boundary and percentage shown within each plot indicate the population selected for the next level of analysis."

References

1. Planas, D., Staropoli, I., Michel, V., Lemoine, F., Donati, F., Prot, M., Porrot, F., Guivel-Benhassine, F., Jeyarajah, B., Brisebarre, A., et al. (2024). Distinct evolution of SARS-CoV-2 Omicron XBB and BA.2.86/JN.1 lineages combining increased fitness and antibody evasion. Nature Communications *15*, 2254. 10.1038/s41467-024-46490-7.
2. Wang, W., Bhushan, G., Paz, S., Stauft, C.B., Selvaraj, P., Goguet, E., Bishop-Lilly, K.A., Subramanian, R., Vassell, R., Lusvarghi, S., et al. (2024). Human and hamster sera correlate well in identifying antigenic drift among SARS-CoV-2 variants, including JN.1. J Virol *98*, e0094824. 10.1128/jvi.00948-24.

We thank the reviewers once again for the careful evaluation of our manuscript "JN.1-adapted vaccination is associated with readjustment of ancestral memory B cells toward neutralization within the JN.1 antigenic space". We are delighted that Reviewers #2, #4 and #5 are satisfied with the revisions made in response to the previous round of comments and have no further concerns. We provide a brief point-by-point response below for completeness.

Reviewer #2 (Remarks to the Author):

The authors have addressed all the raised comments accordingly. No further comments.

Response: We thank Reviewer #2 for their time and careful evaluation of our manuscript across both rounds of review, and for confirming that all previously raised comments have been fully addressed. No further changes have been made in response to this comment.

Reviewer #4 (Remarks to the Author):

I appreciate the additional revisions.

Response: We thank Reviewer #4 for their constructive feedback throughout the review process and their positive assessment of the additional revisions made to the manuscript. No further changes have been made in response to this comment.

Reviewer #5 (Remarks to the Author):

I co-reviewed this manuscript with one of the reviewers who provided the listed reports. This is part of the Nature Communications initiative to facilitate training in peer review and to provide appropriate recognition for Early Career Researchers who co-review manuscripts.

Response: We thank Reviewer #5 for their contribution to the review of our manuscript as part of the co-review training initiative. We note that this comment is administrative in nature and does not raise any concerns requiring a response or manuscript revision.

We thank the reviewers once again for their time and constructive input throughout the review process, which has substantially strengthened the manuscript.
